# Meta-CF_3_-Substituted Analogues of the GFP Chromophore with Remarkable Solvatochromism

**DOI:** 10.3390/ijms24129923

**Published:** 2023-06-08

**Authors:** Maxim M. Perfilov, Elvira R. Zaitseva, Nadezhda S. Baleeva, Vadim S. Kublitski, Alexander Yu. Smirnov, Yulia A. Bogdanova, Svetlana A. Krasnova, Ivan N. Myasnyanko, Alexander S. Mishin, Mikhail S. Baranov

**Affiliations:** 1 Institute of Bioorganic Chemistry, Russian Academy of Sciences, Miklukho-Maklaya 16/10, Moscow 117997, Russiaconzbutcher@gmail.com (I.N.M.);; 2 Laboratory of Medicinal Substances Chemistry, Institute of Translational Medicine, Pirogov Russian National Research Medical University, Ostrovitianov 1, Moscow 117997, Russia; 3 Center of Molecular and Cellular Biology, Skolkovo Institute of Science and Technology, Bolshoi Blvd. 30, Bld. 1, Moscow 121205, Russia

**Keywords:** GFP, Kaede, fluorogen, fluorescence, endoplasmic reticulum

## Abstract

In this work, we have shown that the introduction of a trifluoromethyl group into the me-ta-position of arylidene imidazolones (GFP chromophore core) leads to a dramatic increase in their fluorescence in nonpolar and aprotic media. The presence of a pronounced solvent-dependent gradation of fluorescence intensity makes it possible to use these substances as fluorescent polarity sensors. In particular, we showed that one of the created compounds could be used for selective labeling of the endoplasmic reticulum of living cells.

## 1. Introduction

Fluorescent labeling is a crucial technology of modern biological science [1,2,3,4,5]. In the last decade, a new class of compounds called fluorogens has emerged as an alternative to conventional fluorescent labels [6]. Unlike conventional labels, fluorogens do not have their own pronounced fluorescence in a free form in an aqueous medium. However, they are capable of acquiring it upon binding to various target structures such as proteins [7,8,9,10,11,12,13], nucleotides [14,15,16,17], or even whole organelles [18,19,20,21,22]. Various methine dyes [20,23], including diverse arylidene-azolones [14,15] and derivatives of fluorescent protein chromophores [7,8,9,11,12,18,19,22], are widely used as such labels. Free rotation or arylidene moiety allows these substances to nonradiatively release excitation energy in solutions [24]. However, this energy release can be blocked in the cavity of the protein or nucleotide, as well as by other micro-environment factors [7,8,9,10,11,12,13,14,15,16,17,18,19,20,21,22].

**Scheme 1:**
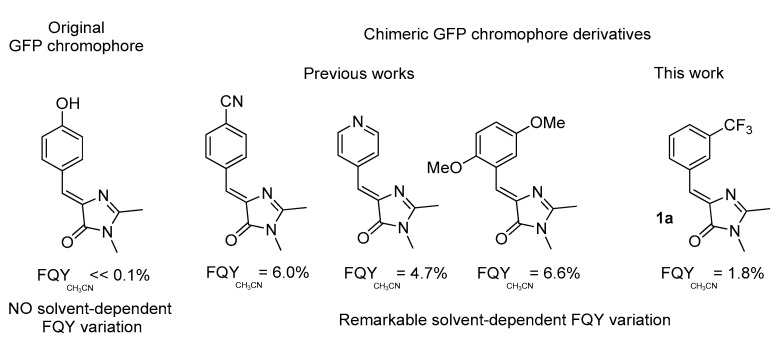
Analogues of GFP chromophore with remarkable solvent-dependent FQY variations.

During our systematic study focused on the development of novel fluorogenic analogs of the GFP chromophore (Scheme 1), we created a derivative containing a trifluoromethyl group at the meta-position of the arylidene moiety—compound **1a** (Scheme 1). Thorough examination of the optical properties of this compound unveiled a distinct property; unlike the majority of arylidene-imidazolones, which are characterized by a low fluorescence quantum yield (FQY) across all solvents, compound **1a** is characterized by noticeable emission in aprotic and non-polar media.

Previously, this behavior had only been observed in a limited range of compounds that either contained electron-acceptor groups in the para-position (e.g., nitro, nitrile, or carboxyl groups [25], as well as 4-pyridile derivatives [18,19]), or simultaneously possessed two electron-donating substituents in the opposite ortho- and meta-positions (Scheme 1) [25,26]. These compounds were used as “polarity sensors” in fluorescent microscopy for labelling of the endoplasmic reticulum (ER) [18,22,25], mitochondria [19], and lipid droplets of live cells [27]. The presence of such pi-electron-withdrawing or pi-electron-donating groups should obviously change the nature of the frontier orbitals, thereby affecting fluorescence intensity. At the same time, the similar effect of the trifluoromethyl group is less expected, which prompted us to study this phenomenon in more detail.

Thus, in the present work, we created a series of derivatives with the meta-CF_3_ group containing an extended conjugated pi-system along with electron-donating substituents. It allowed us to reveal the area in which the fluorogenic effect of the CF_3_ group persists, and also to successfully use one of the obtained compounds for selective staining of the endoplasmic reticulum (ER) in live cells.

The endoplasmic reticulum is an important organelle that forms a network of tubules across the entire cell. The ER assumes a pivotal role in secretory pathways, [28] protein folding, and also lipid biosynthesis [29]. The overload of unfolded proteins within the ER lumen, leading to the formation of aggregates, gives rise to a condition known as ER stress [30]. ER stress is frequently associated with pathological conditions of cells, such as hypoxia or hypoglycemia [31]. Therefore, the ER is an important target for the investigation of cell pathologies. However, existing ER trackers suffer from several limitations, such as location of absorption maxima in the phototoxic UV region [32] or binding to proteins instead of directly labeling ER membranes (differences in expression of such proteins among different cell types can lead to variations in labeling) [33]. Thus, a membrane-localized label with optimal spectral properties is needed.

## 2. Results

**Scheme 2:**
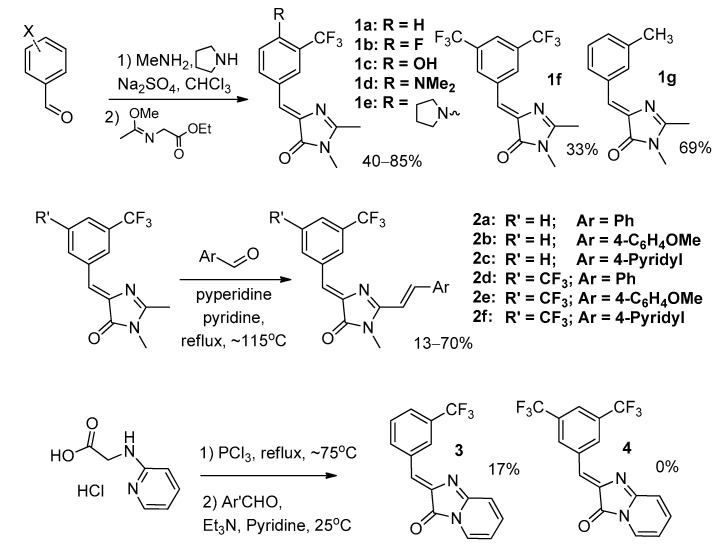
Synthesis of all compounds presented in this work.

The synthesis of the main compound **1a** and its analogues was carried out according to classical technology, starting from corresponding aldehydes (Scheme 2) [34].

Compounds **2** with an extended conjugated pi-system were created through the condensation of compounds **1** with aldehydes in basic media [18,22,35]. Compound **3** was created using 2-(pyridin-2-ylamino)acetic acid as the starting material [36]. However, unlike previous works, the yield of the target product in this case was much lower. Moreover, we were unable to obtain derivative **4** with two trifluoromethyl groups at all.

Next, we studied the optical properties of all compounds in various solvents. The results are presented in Table 1 and Table 2. The spectra are shown in Figure 1, Figure 2 and Figures S1–S13.

The parent compound **1a**, as many other similar GFP chromophore analogs, was characterized by very-short-wavelength positions of absorption and emission maxima. This property renders it unsuitable for fluorescent labeling in living systems due to the high phototoxicity of corresponding excitation light.

One of the most effective approaches to significantly shift the maxima of benzylidene-imidazolones to the long-wave region is to introduce the EDG into the para-position of the benzylidene moiety. However, all compounds **1** with such substituents were characterized by low FQY in all solvents. Even the compound **1b**, where only the fluorine atom was introduced at the para-position, also showed no solvent-dependent FQY variations and demonstrated low emission across all media. The control substance **1g**, which contained an isosteric methyl group in the meta-position, was also completely non-fluorescent. The only successful modification that retained pronounced emission in non-polar solvents was the introduction of a second trifluoromethyl group at the other meta-position—compound **1f**. However, the substance was characterized by similar short-wavelength spectral positions and was also very lipophilic and insoluble in water.

**Figure 2:**
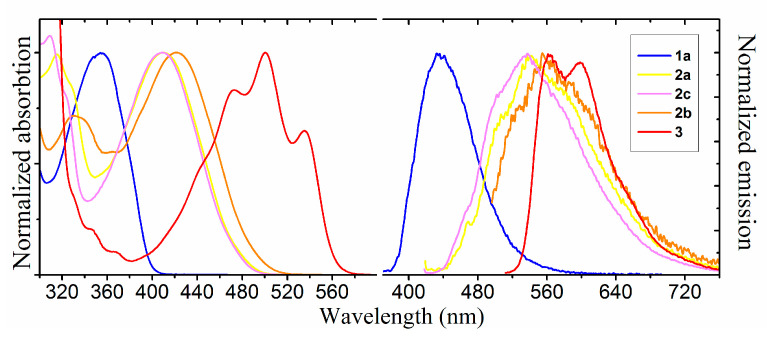
Fluorescence and absorption spectra of compounds **1a**, **2a**–**c** and **3** in acetonitrile.

Another modification that can lead to the red-shift of absorption and emission maxima is the expansion of the conjugated pi-system. As we have previously shown [18,22,25], this modification allows the solvent-dependent FQY variation to remain for other chromophores. We obtained similar results for compounds **1a** and **1f**—all expanded analogues **2** and **3** also demonstrate high FQY in aprotic and non-polar media and relatively low FQY in water (Table 2, Figure 2).

The introduction of a more electron-donating aryl substituent with a methoxy group into the styrene fragment (compounds **2b** and **2f**) led to a more significant red-shift; however, a noticeable decrease in the fluorescence quantum yield was observed. This observation aligns well with the synthesis results. Both substances **2b** and **2f** were obtained as a mixture of E and Z isomers at the styrene part, which indicates a lower isomerization barrier and, hence, easier release of excitation energy. The most substantial red-shift was observed for derivative **3**, which correlates well with previously published data [18,19,25]. At the same time, compounds **2** were characterized by a very large Stokes-shift (more than 100 nm), due to which their emission spectra were close to those of derivative **3**, while the absorption spectra lay in the shorter wavelength region (Figure 2).

In addition to compounds with high fluorescence intensity in solutions, compounds with pronounced fluorescence in solid or aggregated form are also used in fluorescent labeling [37,38]. Chromophore aggregation generally quenches light emission. However, in some cases, the opposite effect has been also observed. This phenomenon, known as aggregation-induced emission (AIE), has garnered considerable interest in recent years, not only as a property of individual substances but also as a whole area of research encompassing the development of new fluorescent labels for living systems and various new materials [39,40,41,42,43]. We found that majority of the compounds described in the article are characterized by pronounced fluorescence in a solid crystalline form (Supplementary Materials—Solid-State and Aggregation-Induced Emission, Figures S14 and S15). This fluorescence was particularly strong for amino-containing compounds **1d** and **1e**, while only derivatives **1c** and **3** were non-fluoresent in the solid state at all. Furthermore, we examined the behavior of the proposed fluorogens when their solutions in DMSO were added to various mixtures of water and acetone (Supplementary Materials—Solid-State and Aggregation-Induced Emission, Figures S16–S28). Compounds **1a** and **3**, which are fluorescent in solution, were less fluorescent at a higher water concentration, which indicates aggregation-induced emission quenching. In contrast, compounds **1d** and **1e** were more fluorescent in such mixtures and non-fluorescent at higher acetone concentrations, which indicates aggregation-induced emission enhancement. Notably, compound **1f** and all compounds **2** were fluorescent across all mixtures, which is less typical among various fluorophores and dyes.

**Figure 3:**
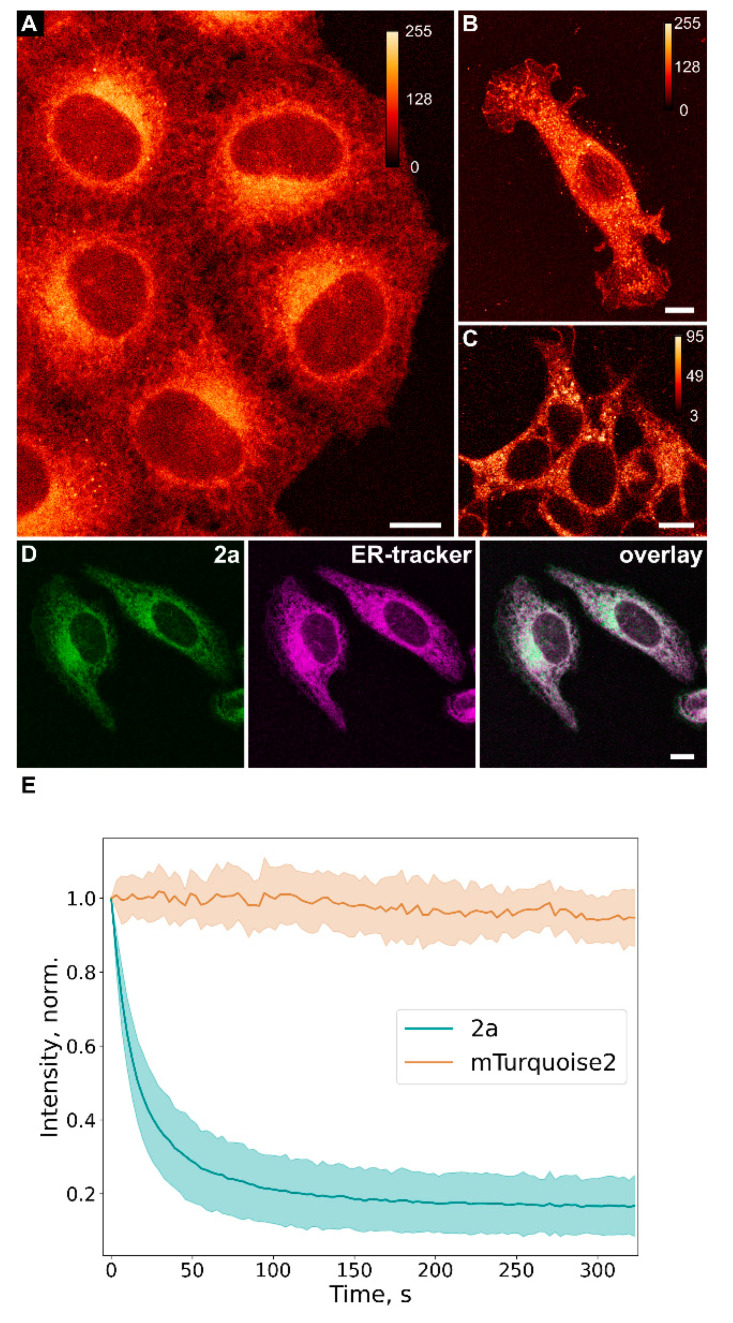
Live-cell imaging with fluorogenic dye **2a** using confocal microscopy. ER staining of HeLa Kyoto (**A**), CT26 (**B**), and HEK293T (**C**) cells using 5 μM of **2a** (added from 5 mM DMSO stock solution, incubation 1 min). Scale bars are 10 μm. Images were processed with Fiji Lookup Tables “NanoJ-Orange”. (**D**) Co-localization analysis of 5 μM of **2a** dye in HeLa Kyoto cells compared to 1 μM ER tracker Red (Invitrogen, added from 1 mM stock in DMSO, incubation 5 min). The scale bar is 10 μm. Pearson co-localization analysis followed by a Costses randomized test resulted in 0.72. (**E**) Photostability analysis in HeLa Kyoto cells stained with 5 μM **2a** in comparison with ER-localized fluorescent protein (mTurquoise2). The time-lapse regime with 9 μW of the 458 nm laser line was used. Data are shown as mean ± SD.

Finally, we tested the behavior of the created substances in HeLa Kyoto cell cultures. As observed with many other substances with high solvent-dependent fluorescence quantum yield variation, in most cases, we observed off-target staining of a wide variety of cell structures simultaneously. However, in the case of compound **2a**, we observed highly selective ER localization of fluorescence (Figure 3A). The tubular network was clearly distinguishable as well as the structures around the nuclei. We also showed ER-staining of CT26 and HEK293T cells (Figure 3B,C). Staining with compound **2a** demonstrated a high level of co-localization with commercially available ER tracker Red (Invitrogen, Waltham, MA, USA): the corrected Pearson coefficient was about 0.72 for HeLa Kyoto cells (Figure 3D). The compound **2a** ER staining demonstrated relatively fast photobleaching compared to a fluorescent protein with a similar absorption spectrum (mTurquoise2) (Figure 3E). Despite such rapid bleaching, we spotted no visible changes in cell morphology throughout the experiment, which indicates the low phototoxicity of the proposed dye.

## 3. Discussion

The solvent-dependent variation of fluorescence quantum yield, which appeared in meta-trifluoromethyl arylidene-imidazolones, became a unique phenomenon among related substances. We synthesized a series of derivatives with this substituent and showed that the introduction of additional groups into the arylidene moiety eliminates the effect, while the imidazolone fragment can be modified while maintaining FQY variation. A noticeable increase in the FQY variation effect can be achieved through introducing a second trifluoromethyl group; however, the obtained substances turn out to be very hydrophobic and practically insoluble in water. The parent substance was characterized by a very-short-wavelength position of the absorption and emission spectra, which makes it difficult to use it as a label for living systems. As in many other cases, an increase in the size of the system of conjugated bonds leads to a noticeable spectral red-shift, and the best effect can be achieved for auron-like structures (compound **3**). Interestingly, the largest red-shift was observed for substances with more electron-donating substituents in the imidazolone moiety, which is not typical for arylidene imidazolones. Apparently, this is caused by polarity reversal due to the presence of an acceptor trifluoromethyl group in the arylidene part of the molecule.

The remarkable solvent-dependent variation of the fluorescence quantum yield often enables the use of dyes as fluorogenic “polarity sensors” for staining some components within living cells. Most often, the simple addition of dyes to the cell medium leads to non-targeted and heterogeneous fluorescent staining of various cell membranes. However, in certain cases, this staining is more selective. The same scenario was observed for the trifluoromethyl derivatives proposed in this article. Most of them stained cells in an untargeted way, but one of the dyes (compound **2a** with styrene moiety) selectively stained the ER of live cells. We have shown that this ER staining is maintained across a wide variety of cell lines, and selectivity is confirmed via co-localization with a commercial ER tracker. Nevertheless, labeling stability in comparison to a fluorescent protein with similar photo properties was very low, not allowing long-term image acquisition. However, the proposed dye did not affect the cell morphology when added to the medium or when irradiated, which indicates its low toxicity and phototoxicity under the conditions we used.

**Figure 4:**
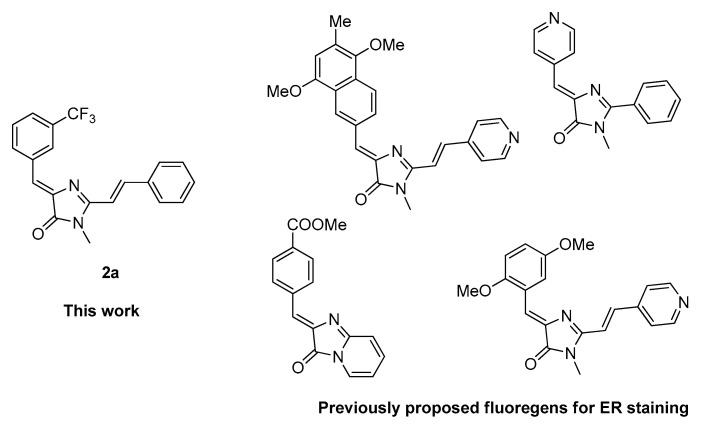
Compound **2a** in comparison with previously proposed fluorogenic ER dyes [18,22,25].

An analysis of the structures of the obtained substances does not reveal any regularity between the structure and the ability of ER-selective staining (Figure 4). Apparently, as in other cases, this selectivity is an accidental result of a successful combination of solvatochromic properties and a certain degree of polarity of the created substance.

The study of the solid or precipitated forms of the proposed compounds has shown the bright fluorescence of many of them (Supplementary Materials—Solid-State and Aggregation-Induced Emission). Such solid-state fluorescence is a common phenomenon for various fluorophores, including arilidene-imidazolones, especially with amino substituents [44]. This effect, known as aggregation-induced emission (AIE), can be observed both in crystalline form and for compounds sedimented through high water concentration. It is known that substances with a pronounced AIE tend to be non-fluorescent in solution, while soluble fluorophores, on the contrary, are not fluorescent in solid form [41,42,43]. Thus, the solid-state fluorescent properties of presented compounds **1a**–**e** and **3** are quite typical. Nevertheless, the high brightness of the emission of amino derivative **1d** and **1e** crystals makes them promising candidates as solid-state emitters for future studies. In contrast, substances that exhibit good fluorescence both in solution and in solid form are particularly intriguing. This behavior was observed for all compounds **2** and the derivative **1f**. It is likely that the presence of two trifluoromethyl groups or a styrene substituent hinders pi–pi stacking, which usually leads to fluorescence quenching in the aggregated state. In particular, we found that derivative **2a**, which stains ER selectively, was especially fluorescent both in solution and in solid or aggregated states, which may somehow explain its selectivity in living cells. However, other non-selective fluorogens **2** also exhibited such emission, which does not allow us to draw any definitive conclusions regarding the relationship between these properties.

## 4. Materials and Methods

### 4.1. Synthesis

Reagents available from commercial sources (Merck (Darmstadt, Germany), ABCR (Karlsruhe, Germany), BLDPharm (Shanghai, China), Fluorochem (Hadfield, UK), TCI (Tokyo, Japan)) were used as is without further purification. Ethyl((methoxy)amino)acetate used in the synthesis of compounds **1** was prepared from glycine ester and acetonitrile according to the approach described earlier [45]. 2-(pyridin-2-ylamino)acetic acid hydrochloride, necessary for the synthesis of compound **3**, was synthesized from 2-amino pyridine and glyoxal according to previously proposed procedures [46].

Flash chromatography was performed with E. Merck Kieselgel 60.

Silica gel 60 F254 glass-backed and aluminum-backed plates (Merck, #cat 105554 and #cat 100390) were used for thin layer chromatography (TLC). Visualization of TLC plates was carried out using UV light (254 or 312 nm) and staining with potassium permanganate solution in water.

700 MHz Bruker Avance III NMR and Avance III 800 (with a 5 mm CPTXI cryoprobe) were used for NMR analysis (at 303 K both). Chemical shifts in the NMR spectra are given relative to the residual peaks of the used solvents (7.27 ppm and 77.0 ppm in CDCl_3_ and 2.51 ppm and 39.5 ppm in DMSO-d_6_ for ^1^H and ^13^C, respectively).

SMP 30 were used for melting point analysis.

Bruker micrOTOF II with electrospray ionization (ESI) was used for high-resolution mass spectra (HRMS) analysis. Analyses were performed in a positive ion mode (4500 V interface capillary voltage) in 50–3000 *m*/*z* mass range. Calibration was performed with ESI Tuning Mix (Agilent, #cat G1969-85000). All samples were dissolved in acetonitrile, methanol, or water and injected via syringe (3 mL/min). Nitrogen was used as a dry gas (interface temperature—180 °C).

Description of the obtained NMR and HRMS spectra, as well as the appearance and melting points of the obtained compounds are presented in Supplementary Materials—Synthesis Results. ^1^H and ^13^C spectra are presented in Supplementary Materials—Copies of ^1^H and ^13^C NMR Spectra.

#### 4.1.1. Synthesis of 4-Amino-3-(Trifluoromethyl)benzaldehydes

2.8 g (15 mmol) of 4-fluoro-3-(trifluoromethyl)benzaldehyde, corresponding amine (20 mmol), and 3 g of potassium carbonate (22.5 mmol) were dissolved in 40 mL of dry DMF and the resulting mixture was stirred for 6 h at 150 °C. Next, the solution was cooled to room temperature and diluted with EtOAc (150 mL). The obtained mixture was washed with saturated KCl solution (3 × 50 mL), dried over Na_2_SO_4,_ and evaporated. The product was purified via flash chromatography (Hexane and EtOAc mixture, *v*/*v* 9:1was used as the eluent).

#### 4.1.2. Synthesis of Compounds **1**

The corresponding starting benzaldehyde (5 mmol) was mixed with methylamine solution (40% in water, 1.75 mL) in CHCl_3_ (25 mL). Pyrrolidine (3 mg, 0.05 mmol) and anhydrous Na_2_SO_4_ (5 g) were added and the mixture was stirred for two days at room temperature. Next, the solids were removed through filtration. The residual solution was dried over additional Na_2_SO_4_ and evaporated. Ethyl((methoxy)amino)acetate (1.59 g, 10 mmol) was added to the residue and mixed. If no complete dissolution was observed during the mixing, then 5–20 mL of dry methanol was added to the mixture. The resulting solution was stored for one day at room temperature. The resulting mixture was then dried under reduced pressure. Finally, the product was purified via flash chromatography (CHCl_3_ and EtOH mixture, *v*/*v* 100:1 was used as the eluent).

#### 4.1.3. Synthesis of Compounds **2**

The corresponding imidazolone **1** (1 mmol) was dissolved in 5 mL of dry pyridine. Corresponding aldehyde (5 mmol) and pyperidine (10 mg) were added and the resulting mixture was refluxed for 4–24 h (controlled via TLC). Next, the mixture was evaporated to dryness under reduced pressure. Finally, the product was purified via flash chromatography (CHCl_3_ and EtOH mixture, *v*/*v* 100:1 was used as the eluent).

#### 4.1.4. Synthesis of Compound **3**

Imidazo [1,2-a]pyridin-3(2H)-one hydrochloride (0.5 g, 2.7 mmol) was suspended in phosphorus trichloride (5 mL) and refluxed for four hours. Next, the mixture was evaporated to dryness under reduced pressure. Dry pyridine (4 mL), triethylamine (0.5 mL) and 2 mmol of corresponding aldehyde were added to the residue, and the mixture was stirred at room temperature for two hours. All these transformations should be performed in an inert atmosphere to avoid hydrolysis. Next, the mixture was evaporated to dryness under reduced pressure and dissolved in CHCl_3_ (100 mL). The obtained solution was washed with potassium carbonate solution (10% in water, 2 × 50 mL), water (2 × 50 mL) and saturated KCl solution (2 × 50 mL), dried over Na_2_SO_4,_ and evaporated. Finally, the product was purified via flash chromatography (CHCl_3_ was used as the eluent). To completely remove impurities, the product was additionally washed with diethyl ether (5 mL) and dried in vacuo.

### 4.2. Spectra of Chromophores

Varian Cary 100 spectrophotometer (Spectralab Scientific Inc., Markham, ON, Canada) and Agilent Cary Eclipse fluorescence spectrophotometer (Agilent Technologies, Santa Clara, CA, USA) were used to record UV-VIS and emission spectra.

The extinction coefficient was calculated using the formula:

(1) $$ɛ=\frac{A}{cl}$$

where c is the molar concentration of the complexes, A is the absorbance intensity at maxima, and l is the pathlength (for all our measurements is 1 cm).

Fluorescence quantum yields of fluorogens were calculated in accordance with a previously proposed protocol [47]. Quinine sulfate, Coumarin 153, and Fluorescein were used as the standards for compounds **1**, **2**, and **3**, respectively. All measurements were performed using 3–4 various concentrations (3–20 µM).

The FQY was calculated using the formula:

(2) $${FQY}_{x}={FQY}_{st}\times \frac{F_{x}}{F_{st}}\times \frac{1-10^{-A_{st}^{}}}{1-10^{-A_{x}^{}}}\times \frac{n_{x}^{2}}{n_{st}^{2}}$$

where F is the area under the emission peak, A is absorbance at the excitation wavelength, n is the refractive index of the solvent, the subscript x corresponds to the studied compounds, and the subscript st is used for standards.

All absorption and emission spectra are presented in Supplementary Materials—Solvatochromic Properties. All graphs were processed using Origin v8.6 software.

### 4.3. Solid-State and Aggregation-Induced Emission

Solid-state and aggregation-induced emission were investigated using a 365 nm diode lamp (Evoluchem, LG/CREE, HCK1012-01-011, 9 mW/cm^2^). For AIE experiments, acetone and water were mixed in various ratios. Next, 1 mL of these solutions was placed in a fluorometric cuvette, and 20 µL of fluorogens 5 mM solution from in DMSO was added. The mixtures were stirred rapidly, placed under the diode lamp and photographed. All photos are presented in Supplementary Materials—Solid-State and Aggregation-Induced Emission.

### 4.4. Fluorescent Imaging in Living Cells

For living cell staining experiments, all fluorogens were dissolved in Dimethylsulfoxide (Sigma Aldrich, St. Louis, MO, USA, “for molecular biology” grade. #cat D8418) in 5 mM concentration and stored at −20 °C in a dark place for no more than 3 months.

In the present study, we used HeLa Kyoto, CT26 (colon carcinoma), and HEK293T (human embryonic kidney 293T) cells. These cell lines were obtained from frozen stocks in our laboratory. Before imaging, cells were seeded into 35 mm glass-bottom dishes (SPL Life Sciences, Pocheon-si, Gyeonggi-do, Republic of Korea) and grown in DMEM medium (PanEco, Singapore) with 10% (*v*/*v*) of fetal bovine serum (HyClone, ThermoScientific, Waltham, MA, USA), 2 mM of glutamine, 4.5 g/L of glucose (PanEco), 50 U/mL of Streptomycin (PanEco), and 50 μg/mL of Penicillin (PanEco) at 37 °C and 5% CO_2_ for 24 h.

After that, cells were transferred in 1 mL Hank’s Balanced Salt Solution with calcium and magnesium (PanEco), containing 20 mM of HEPES (pH 7.1, Sigma-Aldrich) and imaged at room temperature.

For fluorogen staining, cells were incubated with 1.0–10 μM of a dye for one minute before imaging. For co-localization analysis, cells were also incubated with 1 μM of ER-tracker Red (added from the 1 mM stock solution in DMSO) for 5 min before imaging.

A TSC SP2 confocal system (Leica Microsystems, Wetzlar, Germany) installed on inverted fluorescent microscope Leica DM IRE with HCX PL APO Lbd.BL 63 × 1.40 oil immersion lens and argon (458/488 nm) laser was used for confocal microscopy. A 458 nm line of the argon laser (≈6.5 μW) and emission in the 470–530 nm range for mTurquoise2 imaging and ≈9.0 μW of 458 nm line with a 490–590 nm emission range for **2a** imaging were used.

Photobleaching analysis of compound **2a** was performed in HeLa Kyoto cells. Fluorescent protein mTurquoise2 was introduced in similar cells and used as a reference. To introduce such a reference, 24 h after seeding on a dish, HeLa Kyoto cells were transfected using a mixture of corresponding DNA (1.5–2 ng) and 5 μL FuGENE HD transfection reagent (Promega, Madison, WI, USA) in 100 μL OptiMEM (Gibco, Billings, MT, USA) per dish and incubated in a humidified atmosphere at 37 °C and 5% CO_2_ for 24 h. The other group of dishes with HeLa Kyoto cells passed through the same procedure without the transfection step and were incubated with 5 μM of compound **2a** for one minute. Images were acquired with the abovementioned TSC SP2 confocal system with argon (458/488 nm) laser. The 14 × 10^3^ μm^2^ region was scanned at 0.3 fps with 9 μW of the 458 nm line to obtain bleaching curves.

Image processing and co-localization analysis were performed using Fiji ImageJ software (ver. 1.52n) [48] and NanoJ plug-in (ver. 1.14stable1) [49]. Bleaching graphs were processed using custom scripts in Python 3.5.

## 5. Conclusions

In this work, we have shown that the introduction of a trifluoromethyl group into the meta-position of arylidene imidazolones leads to the appearance of pronounced fluorescence in aprotic and nonpolar media. We synthesized a group of various derivatives and showed that the effect of solvent-dependent variation of FQY is retained for substances with diverse substituents in the imidazolone moiety and disappears when new groups are introduced into the arylidene part. It is absolutely not typical for other arylidene imidazolones, with the exception of several groups of substances with a special structure. Thus, we have created a new group of fluorescent polarity sensors based on this popular core.

We have also found that most of the created compounds have pronounced fluorescence in solid crystalline form. This fluorescence turned out to be especially bright for amino derivatives **1d** and **1e**, which makes them promising for further studies. Compound **2a**, which was found to be selective for ER staining, was highly emissive both in the solid state and in solution. This may be a clue for effective ER selectivity, but the data are too thin to make such a deep conclusion in the present study.

We have also shown that one of the proposed substances can be used for selective fluorescence labeling of the endoplasmic reticulum of living cells of various types in short-term experiments. Staining selectivity was confirmed via co-localization with a commercial ER tracker.

Unlike commercially available ER trackers, which rely on specific protein binding, the dye proposed in this work stains the ER solely through a unique combination of lipophilic and fluorogenic properties. The dye proposed by us is completely devoid of a number of the disadvantages of existing trackers, such as incorrect labeling of ER structures due to the absence of these proteins or variation in its expression in any specific cells.

## Figures and Tables

**Figure 1:**
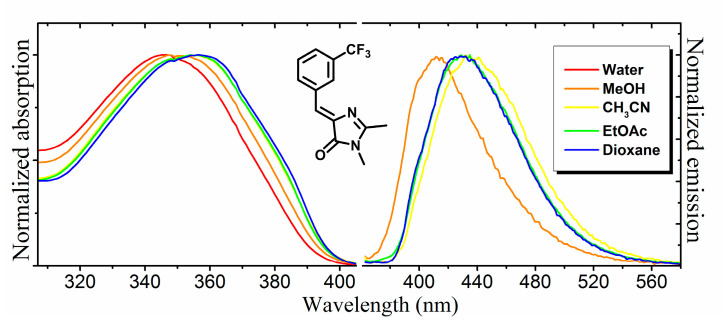
Fluorescence and absorption spectra of compound **1a** in various solvents.

**Table 1:** Optical properties of compounds **1** in various solvents at 1–10 µM concentration.

		1a	1b	1c	1d	1e	1f	1g
Water	Abs ^a^	346 (12,000)	346 (- ^c^)	361 (24,000)	372 (- ^c^)	425 (- ^c^)	346 (- ^c^)	348 (21,000)
	Em ^b^	- ^d^	- ^d^	- ^d^	- ^d^	- ^d^	- ^c^	- ^d^
MeOH	Abs	349 (12,000)	350 (18,500)	367 (23,500)	388 (30,000)	413 (47,000)	355 (17,500)	348 (19,500)
	Em	414 (1.4)	410	410	- ^d^	- ^d^	443 (5)	~430 (- ^d^)
CH_3_CN	Abs	353 (12,000)	354 (18,000)	364 (24,000)	384 (33,500)	405 (44,500)	359 (18,500)	354 (19,000)
	Em	435 (1.8)	425	405	- ^d^	- ^d^	446 (15)	~430 (- ^d^)
EtOAc	Abs	355 (12,500)	354 (18,000)	365 (27,500)	382 (34,500)	400 (44,000)	359 (17,500)	354 (20,000)
	Em	430 (5.5)	410	400	- ^d^	~450 (- ^d^)	441 (30)	~420 (- ^d^)
Dioxane	Abs	356 (11,500)	354 (18,500)	367 (23,500)	386 (32,000)	402 (44,000)	361 (17,500)	355 (20,000)
	Em	429 (3.0)	420	400	~450 (- ^d^)	~460 (- ^d^)	437 (37)	~420 (- ^d^)

a—peak maximum in nm (extinction coefficient in (M cm^−1^)); b—peak maximum in nm (FQY in %); c—not soluble enough; d—nonfluorescent.

**Table 2:** Optical properties of compounds **2**–**3** in various solvents at 1–10 µM concentration.

		2a	2b	2c	2d	2e	2f	3
Water	Abs ^a^	413 (18,000)	428 (9000)	402 (17,000)	- ^c^	- ^c^	- ^c^	485 (17,000)
	Em ^b^	~555 (0.7)	~570 (0.3)	534 (4.0)	- ^c^	- ^c^	- ^c^	592 (0.1)
MeOH	Abs	407 (18,000)	422 (9000)	407 (18,000)	411 (21,500)	425 (14,000)	408 (13,500)	492 (18,000)
	Em	536 (1.8)	~550 (0.2)	543 (9.6)	~545 (0.9)	~565 (0.3)	530 (5.8)	563 (2.9)
CH_3_CN	Abs	410 (22,500)	422 (11,000)	408 (18,500)	414 (20,500)	425 (16,000)	409 (14,000)	500 (21,000)
	Em	542 (2.3)	~560 (0.3)	535 (10.2)	~545 (1.1)	~575 (0.4)	530 (5.9)	564 (5.7)
EtOAc	Abs	410 (23,000)	419 (12,000)	410 (21,000)	413 (21,000)	421 (14,000)	410 (13,500)	501 (21,500)
	Em	541 (4.7)	~555 (0.6)	534 (12.4)	540 (1.7)	~565 (0.6)	530 (6.7)	555 (5.7)
Dioxane	Abs	412 (25,000)	421 (14,500)	412 (22,500)	415 (20,000)	423 (14,500)	413 (13,500)	504 (21,000)
	Em	537 (7.7)	~555 (0.7)	534 (15.2)	538 (5.5)	~565 (0.8)	5519 (9.2)	554 (4.2)

a—peak maximum in nm (extinction coefficient in (M cm^−1^)); b—peak maximum in nm (FQY in %); c—not soluble enough.

## Data Availability

Data is contained within the article or Supplementary Materials.

## Supplementary Materials

The following supporting information can be downloaded at: https://www.mdpi.com/article/10.3390/ijms24129923/s1.

## References

[1] Gonçalves M.S.T. (2009). Fluorescent Labeling of Biomolecules with Organic Probes. Chem. Rev..

[2] Chudakov D.M., Matz M.V., Lukyanov S., Lukyanov K.A. (2010). Fluorescent proteins and their applications in imaging living cells and tissues. Physiol. Rev..

[3] Thorn K. (2017). Genetically encoded fluorescent tags. Mol. Biol. Cell.

[4] Specht E.A., Braselmann E., Palmer A.E. (2017). A Critical and Comparative Review of Fluorescent Tools for Live-Cell Imaging. Annu. Rev. Physiol..

[5] Sahoo H. (2012). Fluorescent labeling techniques in biomolecules: A flashback. RSC Adv..

[6] Klymchenko A.S. (2017). Solvatochromic and Fluorogenic Dyes as Environment-Sensitive Probes: Design and Biological Applications. Acc. Chem. Res..

[7] Povarova N.V., Bozhanova N.G., Sarkisyan K.S., Gritcenko R., Baranov M.S., Yampolsky I.V., Lukyanov K.A., Mishin M.S. (2016). Docking-guided identification of protein hosts for GFP chromophore-like ligands. J. Mater. Chem..

[8] Plamont M.A., Billon-Denis R., Maurin S., Gauron C., Pimenta F.M., Specht C.G., Shi J., Quérard J., Pan B., Rossignol J. (2016). Small fluorescence-activating and absorption-shifting tag for tunable protein imaging in vivo. Proc. Natl. Acad. Sci. USA.

[9] Péresse T., Gautier A. (2019). Next-Generation Fluorogen-Based Reporters and Biosensors for Advanced Bioimaging. Int. J. Mol. Sci..

[10] Dou J., Vorobieva A.A., Sheffler W., Doyle L.A., Park H., Bick M.J., Mao B., Foight G.W., Lee M.Y., Gagnon L.A. (2018). De novo design of a fluorescence-activating β-barrel. Nature.

[11] Yampolsky I.V., Zagaynova E.V., Lukyanov S., Lukyanov K.A., Mishin A.S. (2017). Protein labeling for live cell fluorescence microscopy with a highly photostable renewable signal. Chem. Sci..

[12] Povarova N.V., Zaitseva S.O., Baleeva N.S., Smirnov A.Y., Myasnyanko I.N., Zagudaylova M.B., Bozhanova N.G., Gorbachev D.A., Malyshevskaya K.K., Gavrikov A.S. (2019). Red-shifted substrates for FAST Fluorogen-activating protein based on the GFP-like chromophores. Chem. A Eur. J..

[13] Szent-Gyorgyi C., Schmidt B.F., Schmidt B.S., Creeger Y., Fisher G.W., Zakel K.L., Adler S., Fitzpatrick J.A.J., Woolford C.A., Yan Q. (2008). Fluorogen-activating single-chain antibodies for imaging cell surface proteins. Nat. Biotechnol..

[14] Paige J.S., Wu K.Y., Jaffrey S.R. (2011). RNA mimics of green fluorescent protein. Science.

[15] Filonov G.S., Moon J.D., Svensen N., Jaffrey S.R. (2014). Broccoli: Rapid selection of an RNA mimic of green fluorescent protein by fluorescence-based selection and directed evolution. J. Am. Chem. Soc..

[16] Song W., Filonov G.S., Kim H., Hirsch M., Li X., Moon J.D., Jaffrey S.R. (2017). Imaging RNA polymerase III transcription using a photostable RNA–fluorophore complex. Nat. Chem. Biol..

[17] Schoen I., Ries J., Klotzsch E., Ewers H., Vogel V. (2011). Binding-activated localization microscopy of DNA structures. Nano Lett..

[18] Ermakova Y.G., Sen T., Bogdanova Y.A., Smirnov A.Y., Baleeva N.S., Krylov A.I., Baranov M.S. (2018). Pyridinium Analogues of Green Fluorescent Protein Chromophore: Fluorogenic Dyes with LargeSolvent-Dependent Stokes Shift. J. Phys. Chem. Lett..

[19] Ermakova Y.G., Bogdanova Y.A., Baleeva N.S., Zaitseva S.O., Guglya E.B., Smirnov A.Y., Zagudaylova M.B., Baranov M.S. (2019). Pyridine analogue of fluorescent protein chromophore: Fluorogenic dye suitable for mitochondria staining. Dye. Pigment..

[20] Collot M., Kreder R., Tatarets A.L., Patsenker L.D., Melya Y., Klymchenko A.S. (2015). Bright fluorogenic squaraines with tuned cell entry for selective imaging of plasma membrane vs. endoplasmic reticulum. Chem. Commun..

[21] Hua F., Liu B. (2016). Organelle-specific bioprobes based on fluorogens with aggregation-induced emission (AIE) characteristics. Org. Biomol. Chem..

[22] Perfilov M.M., Zaitseva E.R., Smirnov A.Y., Mikhaylov A.A., Baleeva N.S., Myasnyanko I.N., Mishin A.S., Baranov M.S. (2022). Environment-sensitive fluorogens based on a GFP chromophore structural motif. Dye Pigment..

[23] Xu S., Hu H. (2018). Fluorogen-activating proteins: Beyond classical fluorescent proteins. Acta Pharm. Sinica B.

[24] Baranov M.S., Lukyanov K.A., Borissova A.O., Jhamir J., Kosenkov D., Slipchenko L.V., Tolbert L.M., Yampolsky I.V., Solntsev K.M. (2012). Conformationally Locked Chromophores as Models of Excited-State Proton Transfer in Fluorescent Proteins. J. Am. Chem. Soc..

[25] Smirnov A.Y., Perfilov M.M., Zaitseva E.R., Zagudaylova M.B., Zaitseva S.O., Mishin A.S., Baranov M.S. (2020). Design of red-shifted and environment-sensitive fluorogens based on GFP chromophore core. Dye Pigment..

[26] Deng H., Yu C., Gong L., Zhu X. (2016). Self-Restricted Green Fluorescent Protein Chromophore Analogues: Dramatic Emission Enhancement and Remarkable Solvatofluorochromism. J. Phys. Chem. Lett..

[27] Baranov M.S., Solntsev K.M., Baleeva N.S., Mishin A.S., Lukyanov S.A., Lukyanov K.A., Yampolsky I.V. (2014). Red-Shifted Fluorescent Aminated Derivatives of a Conformationally Locked GFP Chromophore. Chem. Eur. J..

[28] Schröder M., Kaufman R.J. (2005). The mammalian unfolded protein response. Annu. Rev. Biochem..

[29] Fagone P., Jackowski S. (2009). Membrane phospholipid synthesis and endoplasmic reticulum function. J. Lipid Res..

[30] Zeeshan H.M.A., Lee G.H., Kim H.-R., Chae H.-J. (2016). Endoplasmic Reticulum Stress and Associated ROS. Int. J. Mol. Sci..

[31] Harding H.P., Ron D. (2002). Endoplasmic reticulum stress and the development of diabetes: A review. Diabetes.

[32] McDonald L., Liu B., Taraboletti A., Whiddon K., Shriver L.P., Konopka M., Liu Q., Pang Y. (2016). Fluorescent flavonoids for endoplasmic reticulum cell imaging. J. Mater. Chem. B.

[33] Hambrock A., Löffler-Walz C., Quast U. (2002). Glibenclamide binding to sulphonylurea receptor subtypes: Dependence on adenine nucleotides. Br. J. Pharmacol..

[34] Baleeva N.S., Baranov M.S. (2016). Synthesis and properties of 5-methylidene-3,5-dihydro-4H-imidazol-4-ones (microreview). Chem. Heterocycl. Compd..

[35] Chuang W.T., Chen B.S., Chen K.Y., Hsieh C.C., Chou P.T. (2009). Fluorescent protein red Kaede chromophore; one-step, high-yield synthesis and potential application for solar cells. Chem. Commun..

[36] Baleeva N.S., Myannik K.A., Yampolsky I.V., Baranov M.S. (2015). Bioinspired fluorescent dyes based on a conformationally locked chromophore of the fluorescent protein kaede. Eur. J. Org. Chem..

[37] Qian J., Tan B.Z. (2017). AIE Luminogens for Bioimaging and Theranostics: From Organelles to Animals. Chem.

[38] Shellaiah M., Sun K.-W. (2022). Pyrene-Based AIE Active Materials for Bioimaging and Theranostics Applications. Biosensors.

[39] Lu Q., Wu C.-J., Liu Z., Niu G., Yu X. (2020). Fluorescent AIE-Active Materials for Two-Photon Bioimaging Applications. Appl. Front. Chem..

[40] Ma J., Gu Y., Ma D., Lu W., Qiu J. (2022). Insights into AIE materials: A focus on biomedical applications of fluorescence. Front. Chem..

[41] Hong Y., Lama J.W.Y., Tang B.Z. (2009). Aggregation-induced emission: Phenomenon, mechanism and applications. Chem. Commun..

[42] Han T., Yan D., Wu Q., Song N., Zhang H., Wang D. (2021). Aggregation-Induced Emission: A Rising Star in Chemistry and Materials Science. Chin. J. Chem..

[43] Mei J., Leung N.L.C., Kwok R.T.K., Lam J.W.Y., Tang B.Z. (2015). Aggregation-Induced Emission: Together We Shine, United We Soar!. Chem. Rev..

[44] Xiang S., GuangXi H., Kan L., GuanXin Z., DeQing Z. (2013). Tuning the solid-state emission of the analogous GFP chromophore by varying alkyl chains in the imidazolinone ring. Sci. China Chem..

[45] Baldridge A., Amador A., Tolbert L.M. (2011). Fluorescence Turn On by Cholate Aggregates. Langmuir.

[46] Alcaide B., Plumet J., Sierra M.A. (1990). One-pot synthesis of N-(2-heteroaryl)-.alpha.-amino esters by the regiospecific 2-N-(.alpha.-alkoxycarbonyl)alkylation of 2-aminoazines and -azoles with glyoxals and alcohols promoted by perchloric acid. J. Org. Chem..

[47] Würth C., Grabolle M., Pauli J., Spieles M., Resch-Genger U. (2013). Relative and absolute determination of fluorescence quantum yields of transparent samples. Nat. Protoc..

[48] Schindelin J., Arganda-Carreras I., Frise E., Kaynig V., Longair M., Pietzsch T., Preibisch S., Rueden C., Saalfeld S., Schmid B. (2012). Fiji: An open-source platform for biological-image analysis. Nat. Methods.

[49] Laine R.F., Tosheva K.L., Gustafsson N., Gray R.D.M., Almada P., Albrecht D., Risa G.T., Hurtig F., Lindås A.-C., Baum B. (2019). NanoJ: A high-performance open-source super-resolution microscopy toolbox. J. Phys. D.

