# Supplementary material for: Meta-CF3-Substituted Analogues of the GFP Chromophore with Remarkable Solvatochromism
Source: Int J Mol Sci. 2023 Jun 8;24(12):9923. doi: 10.3390/ijms24129923 (PMC10298595; doi:10.3390/ijms24129923)
Supplement: Supplementary file 1 [file ijms-24-09923-s001.zip › ijms-2431212-supplementary.pdf]

Supporting Information

1. Synthesis results **S2**

2. Solvatochromic properties **S12**

3. Solid-state and aggregation-induced emission **S17**

4. Copies of ^{1}H and ^{13}C NMR spectra **S25**

1. Synthesis results

4-(dimethylamino)-3-(trifluoromethyl)benzaldehyde

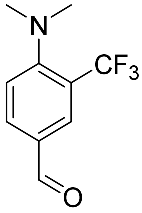

Yield 2.82 g (88%), colorless oil.

^{1}H NMR (700 MHz, DMSO-d_6), δ (ppm): 2.94 (s, 6H), 7.34 (d, *J*=8.6 Hz, 1H), 7.97 (dd, *J*=8.7, 2.0 Hz, 1H), 8.11 (d, *J*=1.9 Hz, 1H), 9.88 (s, 1H).

^{13}C NMR (176 MHz, DMSO-d_6), δ (ppm): 43.5 (2C), 117.9 (q, *J*=30.1Hz, 1C), 120.0 (1C), 124.1 (q, *J*=272.1 Hz, 1C), 127.8 (1C), 131.1 (q, *J*=5.7 Hz, 1C), 132.8 (1C), 155.8 (1C), 190.3 (1C).

HRMS found, *m/z*: 218.0780 $[M+H]^+$. $C_{10}H_{10}F_3NO$. Calculated, *m/z*: 218.0787.

4-(pyrrolidin-1-yl)-3-(trifluoromethyl)benzaldehyde

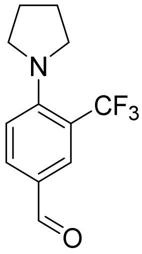

Yield 1.57 g (44%), yellowish solid, m.p. 48-51°C.

^{1}H NMR (700 MHz, DMSO-d_6), δ (ppm): 1.90 - 1.98 (m, 4H), 3.44 - 3.51 (m, 4H), 7.07 (d, *J*=9.0 Hz, 1H), 7.85 (dd, *J*=9.0, 1.9 Hz, 1H), 8.09 (d, *J*=1.9 Hz, 1H), 9.78 (s, 1H).

^{13}C NMR (176 MHz, DMSO-d_6), δ (ppm): 25.3 (2C), 51.1 (2C), 111.3 (q, *J*=31.1 Hz, 1C), 116.8 (1C), 124.3 (1C), 124.6 (q, *J*=271.4 Hz, 1C), 132.2 (2C), 150.5 (1C), 189.7 (1C).

HRMS found, *m/z*: 244.0938 $[M+H]^+$. $C_{12}H_{12}F_3NO$. Calculated, *m/z*: 244.0944.

(Z)-5-(3-(trifluoromethyl)benzylidene)-2,3-dimethyl-3,5-dihydro-4H-imidazol-4-one (1a)

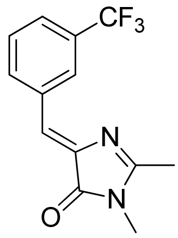

Yield 1150 mg (85%), yellowish solid.

Properties correspond to reported previously[1]

(Z)-5-(4-fluoro-3-(trifluoromethyl)benzylidene)-2,3-dimethyl-3,5-dihydro-4H-imidazol-4-one (1b)

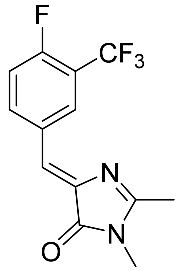

Yield 715 mg (50%), yellowish solid, m.p. 183-186°C.

^{1}H NMR (700 MHz, DMSO-*d*$_6$), δ (ppm): 2.38 (s, 3H), 3.11 (s, 3H), 7.09 (s, 1H), 7.61 (t, *J*=9.7 Hz, 1H), 8.53 - 8.62 (m, 1H), 8.71 (bd, *J*=6.3 Hz, 1H).

^{13}C NMR (176 MHz, DMSO-*d*$_6$), δ (ppm): 15.5 (1C), 26.2 (1C),

117.0 (q, *J*=32.8 Hz, 1C), 117.6 (d, *J*=21.1 Hz, 1C), 121.3 (1C), 122.4 (q, *J*=272.7 Hz, 1C), 130.2 (1C), 130.8 (dq, *J*=270 Hz, *J*=4.2 Hz, 1C), 138.1 (d, *J*=8.7 Hz, 1C), 139.9 (d, *J*=3.0 Hz, 1C), 158.9 (d, *J*=258.0 Hz, 1C), 165.9 (1C), 169.6 (1C).

HRMS found, *m/z*: 287.0800 [M+H]$^+$. $C_{13}H_{10}F_4N_2O$. Calculated, *m/z*: 287.0802.

[1] Anthony Baldridge, Adrian Amador, and Laren M. Tolbert *Langmuir* **2011** *27* (7), 3271-3274

(Z)-5-(4-hydroxy-3-(trifluoromethyl)benzylidene)-2,3-dimethyl-3,5-dihydro-4H-imidazol-4-one (1c)

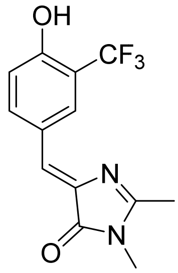

Yield 586 mg (40%), yellow solid, m.p. 216-219°C.

^{1}H NMR (700 MHz, DMSO-*d*$_6$), δ (ppm): 2.34 (s, 3H), 3.09 (s, 3H), 6.97 (s, 1H), 7.08 (d, *J*=8.6 Hz, 1H), 8.28 (dd, *J*=8.6, 1.5 Hz, 1H), 8.51 (d, *J*=1.3 Hz, 1H), 11.20 (br. s., 1H).

^{13}C NMR (176 MHz, DMSO-*d*$_6$), δ (ppm): 15.3 (1C), 26.2 (1C), 115.9 (q, *J*=30.5 Hz, 1C), 117.2 (1C), 123.6 (1C), 123.7 (q, *J*=272.4 Hz, 1C), 125.1 (1C), 130.5 (q, *J*=4.7 Hz, 1C), 137.2 (1C), 137.5 (1C), 157.2 (1C), 163.6 (1C), 169.7 (1C).

HRMS found, *m/z*: 285.0851 [M+H]$^+$. $C_{13}H_{11}F_3N_2O_2$. Calculated, *m/z*: 285.0845.

(Z)-5-(4-(dimethylamino)-3-(trifluoromethyl)benzylidene)-2,3-dimethyl-3,5-dihydro-4H-imidazol-4-one (1d)

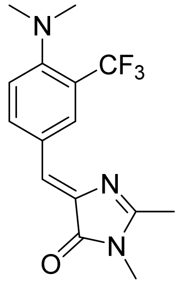

Yield 762 mg (49%), yellow solid, m.p. 152-155°C.

^{1}H NMR (700 MHz, DMSO-*d*$_6$), δ (ppm): 2.35 (s, 3H), 2.80 (s, 6H), 3.10 (s, 3H), 7.00 (s, 1H), 7.37 (d, *J*=8.8 Hz, 1H), 8.33 (dd, *J*=8.6, 1.7 Hz, 1H), 8.57 (d, *J*=1.9 Hz, 1H).

^{13}C NMR (176 MHz, DMSO-*d*$_6$), δ (ppm): 15.4 (1C), 26.2 (1C), 44.3 (2C), 120.5 (q, *J*=28.8 Hz, 1C), 121.3 (1C), 123.1 (1C), 124.2 (q, *J*=273.4 Hz, 1C), 127.5 (1C), 131.4 (q, *J*=5.7 Hz), 136.1 (1C), 138.2 (1C), 153.1 (1C), 164.0 (1C), 169.6 (1C).

HRMS found, *m/z*: 312.1327 [M+H]$^+$. $C_{15}H_{16}F_3N_3O$. Calculated, *m/z*: 312.1318.

(Z)-5-(4-(pyrrolidin-1-yl)-3-(trifluoromethyl)benzylidene)-2,3-dimethyl-3,5-dihydro-4H-imidazol-4-one (1e)

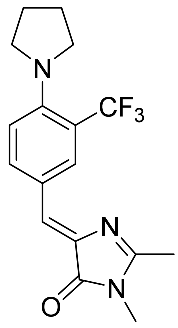

Yield 927 mg (55%), orange solid, m.p. 187-190°C.

Contain 10-15% of E isomer.

^{1}H NMR (700 MHz, DMSO-d_6), δ (ppm): 1.92 - 1.94 (m, 4H), 2.33 (s, 3H), 3.09 (s, 3H), 3.40 - 3.43 (m, 4H), 6.95 (s, 1H), 7.02 (d, *J*=9.0 Hz, 1H), 8.25 (dd, *J*=9.0, 1.9 Hz, 1H), 8.50 (d, *J*=1.9 Hz, 1H).

^{13}C NMR (176 MHz, DMSO-d_6), δ (ppm): 15.3 (1C), 25.2 (2C), 26.1 (1C), 50.9 (2C), 112.8 (q, *J*=29.8 Hz, 1C), 116.8 (1C), 122.2 (1C), 124.2 (1C), 124.7 (q, *J*=272.1 Hz, 1C), 132.6 (brs, 1C), 135.7 (1C), 136.4 (1C), 147.5 (1C), 162.2 (1C), 169.5 (1C).

HRMS found, *m/z*: 338.1481 $[M+H]^+$. $C_{17}H_{18}F_3N_3O$. Calculated, *m/z*: 338.1475.

(Z)-5-(3,5-bis(trifluoromethyl)benzylidene)-2,3-dimethyl-3,5-dihydro-4H-imidazol-4-one (1f)

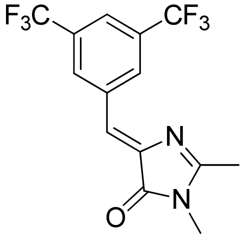

Yield 555 mg (33%), white solid, m.p. 173-176°C.

^{1}H NMR (700 MHz, DMSO-d_6), δ (ppm): 2.40 (s, 3H), 3.12 (s, 3H), 7.19 (s, 1H), 8.08 (s, 1H), 8.93 (s, 2H).

^{13}C NMR (176 MHz, DMSO-d_6), δ (ppm): 15.6 (1C), 26.3 (1C), 120.3 (1C), 122.1 (brs, 1C), 123.2 (q, *J*=272.7 Hz, 1C), 130.5 (q, *J*=32.5 Hz, 1C), 131.5 (1C), 136.7 (1C), 141.5 (1C), 167.3 (1C), 169.5 (1C).

HRMS found, *m/z*: 337.0779 $[M+H]^+$. $C_{13}H_{10}F_4N_2O$. Calculated, *m/z*: 337.0770.

(Z)-1,2-dimethyl-4-(3-methylbenzylidene)-1H-imidazol-5(4H)-one (1g)

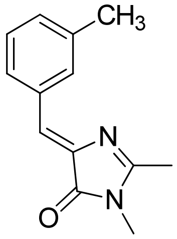

Yellow solid (442 mg, yield 69%).

Properties correspond to reported previously [2].

^{1}H NMR (700 MHz, $CDCl_3$) δ ppm 2.39 (s, 3H), 2.40 (s, 3H), 3.19 (s, 3H), 7.09 (s, 1H), 7.19 (d, J=7.5 Hz, 1H), 7.31 (t, J=7.7 Hz, 1H), 7.93 (s, 1H), 7.95 (d, J=7.7 Hz, 1H).

[2]Baldridge A., Kowalik J., Tolbert L. M. *Synthesis* **2010**, 14, 2424.

3-methyl-2-((E)-styryl)-5-((Z)-3-(trifluoromethyl)benzylidene)-3,5-dihydro-4H-imidazol-4-one (2a)

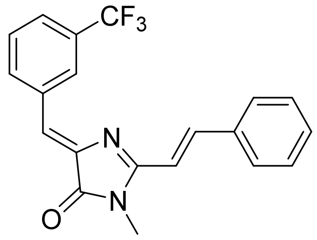

Yield 163 mg (46%), yellow solid, m.p. 138-140°C.

^{1}H NMR (700 MHz, DMSO-*d*$_6$), δ (ppm): 3.30 (s, 3H), 7.15 (s, 1H), 7.29 (d, *J*=15.6 Hz, 1H), 7.44 - 7.52 (m, 3H), 7.71 (t, *J*=7.7 Hz, 1H), 7.73 - 7.78 (m, 1H), 7.86 (d, *J*=7.2 Hz, 2H), 8.04 (d, *J*=15.8 Hz, 1H), 8.64 (d, *J*=7.8 Hz, 1H), 8.70 (s, 1H).

^{13}C NMR (201 MHz, DMSO-*d*$_6$), δ (ppm): 26.4 (1C),

113.8 (1C), 122.4 (1C), 124.0 (q, *J*=272.1 Hz, 1C), 125.6 (d, *J*=3.3 Hz, 1C), 128.0 (q, *J*=3.7 Hz, 1C), 128.4 (1C), 128.9 (1C), 129.4 (q, *J*=31.9, 1C), 129.6 (1C) 130.3 (1C), 134.8 (1C), 135.3 (1C), 135.4 (1C), 140.7 (1C), 141.1 (1C), 161.8 (1C) 169.8 (1C).

HRMS found, *m/z*: 357.1206 $[M+H]^+$. $C_{20}H_{16}F_3N_2O$. Calculated, *m/z*: 357.1209.

2-(4-methoxystyryl)-3-methyl-5-((Z)-3-(trifluoromethyl)benzylidene)-3,5-dihydro-4H-imidazol-4-one (2b)

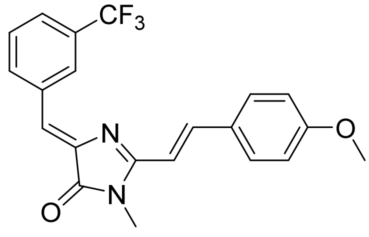

Yield 154 mg (40%), yellow solid, m.p. 153-155°C.

Mixture of E and Z-isomers (styrene fragment, 5:2).

E-isomer: ^{1}H NMR (700 MHz, DMSO-*d*$_6$), δ (ppm) 3.29 (s, 3H), 3.84 (s, 3H), 7.05 (d, *J*=8.8 Hz, 2H), 7.09 (s, 1H), 7.12 (d, *J*=15.6 Hz, 1H), 7.70 (t, *J*=7.7 Hz, 1H), 7.73 (m, 1H), 7.82 (d, *J*=8.8 Hz, 2H), 8.01 (d, *J*=15.6 Hz, 1H), 8.63 (d, *J*=7.6 Hz, 1H), 8.68 (s, 1H).

Z-isomer: ^{1}H NMR (700 MHz, DMSO-*d*$_6$), δ (ppm): 3.12 (s, 3H), 3.80 (s, 3H), 6.34 (d, *J*=13.0 Hz, 1H), 6.94 (d, *J*=9.0 Hz, 2H), 7.16 (d, *J*=13.0 Hz, 1H), 7.18 (s, 1H), 7.64 (t, *J*=7.8 Hz, 1H), 7.74 (m, 1H), 8.04 (d, *J*=8.8 Hz, 2H), 8.42 (d, *J*=8.0 Hz, 1H), 8.57 (s, 1H).

^{13}C NMR (176 MHz, DMSO-*d*$_6$), δ (ppm): Spectrum of isomers mixture described as is. 26.4, 55.3, 111.0, 113.4, 114.5, 121.5, 124.1 (d, *J*=272.4 Hz), 125.5 (d, *J*=4.0 Hz), 127.6, 128.0 (d, *J*=3.3 Hz), 129.4 (d, *J*=31.8 Hz), 129.6, 130.4, 132.5, 135.2, 135.6, 140.9, 141.2, 161.3, 162.1, 169.9. HRMS found, *m/z*: 387.1314 $[M+H]^+$. $C_{21}H_{18}F_3N_2O_2$. Calculated, *m/z*: 387.1315.

3-methyl-2-((E)-2-(pyridin-4-yl)vinyl)-5-((Z)-3-(trifluoromethyl)benzylidene)-3,5-dihydro-4H-imidazol-4-one (2c)

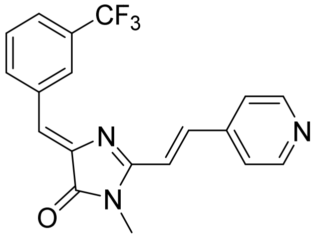

Yield 117 mg (33%), yellow solid, m.p. 170-172°C.

^{1}H NMR (700 MHz, DMSO-*d*6), δ (ppm): 7.22 (s, 1H), 7.53 (d, *J*=16.0 Hz, 1H), 7.71 (t, *J*=7.8 Hz, 1H), 7.75 - 7.78 (m, 1H), 7.80 (d, *J*=5.9 Hz, 2H), 7.96 (d, *J*=15.8 Hz, 1H), 8.62 - 8.71 (m, 4H).

^{13}C NMR (201 MHz, DMSO-*d*6), δ (ppm): 26.5 (1C),

118.6 (1C), 122.0 (1C), 123.8 (1C), 123.9 (q, J=272.5, 1C), 126.0 (q, *J*=3.7 Hz, 1C), 128.3 (q, *J*=4.0 Hz, 1C), 129.5 (q, *J*=31.9 Hz, 1C), 129.7 (1C), 135.2 (1C), 135.5 (1C), 138.2 (1C), 140.5 (1C), 141.7 (1C), 150.3 (1C), 161.3 (1C), 169.7 (1C).

HRMS found, *m/z*: 358.1161 $[M+H]^+$. $C_{19}H_{15}F_3N_3O$. Calculated, *m/z*: 358.1162.

5-((Z)-3,5-bis(trifluoromethyl)benzylidene)-3-methyl-2-((E)-styryl)-3,5-dihydro-4H-imidazol-4-one (2d)

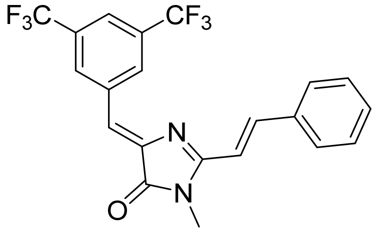

Yield 56 mg (44%), yellow solid, m.p. 221-223°C.

^{1}H NMR (700 MHz, DMSO-*d*6) δ ppm 3.32 (s, 3 H), 7.27 (s, 1 H), 7.33 (d, *J*=15.6 Hz, 1 H), 7.46 - 7.54 (m, 3 H), 7.82 - 7.85 (m, 2 H), 8.01 (d, *J*=15.6 Hz, 1 H), 8.11 (s, 1 H), 9.05 (s, 2 H).

^{13}C NMR (201 MHz, DMSO-*d*6) δ ppm 26.5 (1C), 113.6 (1C), 120.1 (1C), 123.2 (q, J=272.9, 2C), 121.9 (br.s, 1C), 128.5 (2C), 129.0 (2C), 130.5 (q, J=32.6, 2C), 130.6 (1C), 131.6 (br. 2 C), 134.6 (s, 1C), 137.1 (s, 1C), 141.7 (1C), 141.9 (s, 1C), 162.9 (1C), 169.7 (1C)

HRMS (ESI) m/z: 425.1079 found (calcd for $C_{21}H_{15}F_6N_2O^+$, $[M+H]^+$ 425.1083).

5-((Z)-3,5-bis(trifluoromethyl)benzylidene)-2-((E)-4-methoxystyryl)-3-methyl-3,5-dihydro-4H-imidazol-4-one (2e)

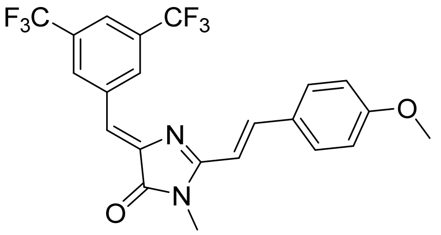

Yield 94 mg (70%), orange solid, m.p. 230-232°C

Mixture of E and Z-isomers (styrene fragment, 3:2).

(E-isomer) ^{1}H NMR (700 MHz, DMSO-d_6) δ ppm 3.28 (s, 3 H), 3.84 (s, 3 H), 7.05 (d, *J*=8.4 Hz, 2 H), 7.13 (d, *J*=15.6 Hz, 1 H), 7.17 (s, 1 H), 7.76 (d, *J*=8.4 Hz, 2 H), 7.95 (d, *J*=15.6 Hz, 1 H), 8.04 (br. s., 1 H), 9.01 (br. s., 2 H)

(Z-isomer)^{1}H NMR (700 MHz, DMSO-d_6) δ ppm 3.13 (s, 3H), 3.76 (s, 3H), 6.32 (d, *J*=13.0 Hz, 1H), 6.89 (m, *J*=8.6 Hz, 2H), 7.16 (d, *J*=13.0 Hz 1 H), 7.27 (s, 1H), 7.99 (m, *J*=8.8 Hz, 2 H), 8.04 (br. s., 1H), 8.83 (br. s., 2H)

(E-isomer) ^{13}C NMR (201 MHz, DMSO-d_6) δ ppm 26.8 (1C), 55.0 (1C), 111.8 (1C), 113.5 (2 C), 121.0 (1C), 123.1 (q, J=273.2, 2 C), 121.8 (br. s., 1C), 127.6 (1C), 130.6 (q, J=33.0, 2 C), 131.3 (br. s., 2 C), 132.4 (2 C), 136.8 (1C), 141.8 (br. s., 1C), 143.2 (1C), 160.6 (1C), 162.7(1C), 169.4 (1C)

(Z-isomer) ^{13}C NMR (201 MHz, DMSO-d_6) δ ppm 26.5 (1C), 55.4 (1C), 110.9 (s., 1C), 114.6 (2 C), 119.2 (s., 1C), 123.3 (q, J=273.3, 2 C), 122.4 (br. s., 1C), 127.4 (1C), 130.6 (q, J=33.0, 2 C), 131.5 (br. s., 2 C), 132.4 (2 C), 137.3 (1C), 141.7 (1C), 142.1 (1C), 161.5 (1C), 163.3 (s., 1C), 169.8 (1C)

HRMS (ESI) m/z: 455.1187 found (calcd for $C_{22}H_{17}F_6N_2O_2^+$, $[M+H]^+$ 455.1189).

5-((Z)-3,5-bis(trifluoromethyl)benzylidene)-3-methyl-2-((E)-2-(pyridin-4-yl)vinyl)-3,5-dihydro-4H-imidazol-4-one (2f)

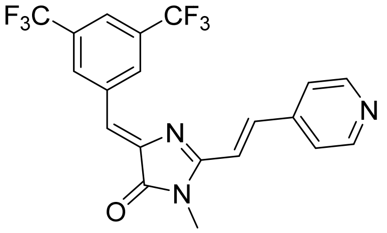

Yield 17 mg (13%), white solid, m.p. 245-247°C

^{1}H NMR (700 MHz, DMSO-d_6) δ ppm 3.32 (s, 3H), 7.35 (s, 1H), 7.58 (d, *J*=15.8 Hz, 1 H), 7.79 (dd, *J*=4.7, 1.2 Hz, 2 H), 7.91 (d, *J*=15.8 Hz, 1H), 8.13 (s, 1H), 8.70 (d, *J*=5.9 Hz, 2H), 9.04 (d, *J*=0.6 Hz, 2H)

^{13}C NMR (201 MHz, DMSO-d_6) δ ppm 26.7 (1C), 118.7 (1C), 123.2 (q, J=272.9, 2 C), 121.7 (1C), 122.1 (2 C), 122.4 (br. s., 1C), 130.7 (q, J=33.0, 2 C), 131.8 (br. s., 2C), 136.9 (1C), 138.7 (1C), 141.6 (1C), 141.7 (1C), 150.5 (2 C), 162.5 (1C), 169.7 (1C)

HRMS (ESI) m/z: 426.1036 found (calcd for $C_{20}H_{13}F_6N_3O^+$, $[M+H]^+$ 426.1035).

(Z)-2-(3-(trifluoromethyl)benzylidene)imidazo[1,2-a]pyridin-3(2H)-one (3)

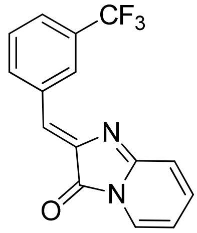

Yield 99 mg (17%), red solid, m.p. 175-178°C.

^{1}H NMR (700 MHz, DMSO-d_6), δ (ppm) :6.43 (t, *J*=6.6 Hz, 1H), 7.04 (d, *J*=9.3 Hz, 1H), 7.30 (s, 1H), 7.40 (ddd, *J*=9.3, 6.3, 1.1 Hz, 1H), 7.70 (t, *J*=7.8 Hz, 1H), 7.73 - 7.76 (m, 1H), 7.78 (d, *J*=6.9 Hz, 1H), 8.54 (d, *J*=7.8 Hz, 1H), 8.74 (s, 1H).

^{13}C NMR (176 MHz, DMSO-d_6), δ (ppm): 109.9 (1C), 118.5 (1C), 123.7 (1C), 124.0 (q, J=272.4, 1C), 125.9 (q, *J*=3.7 Hz, 1C), 126.6 (1C), 128.1 (q, *J*=3.3 Hz, 1C), 129.5 (q, *J*=31.5 Hz, 1C), 129.7 (1C), 135.6 (1C), 135.7 (1C), 139.6 (1C), 139.9 (1C), 157.5 (1C), 167.0 (1C).

HRMS found, *m/z*: 291.0741 $[M+H]^+$. $C_{15}H_{10}F_3N_2O$. Calculated, *m/z*: 291.0740.

2. Solvatochromic properties

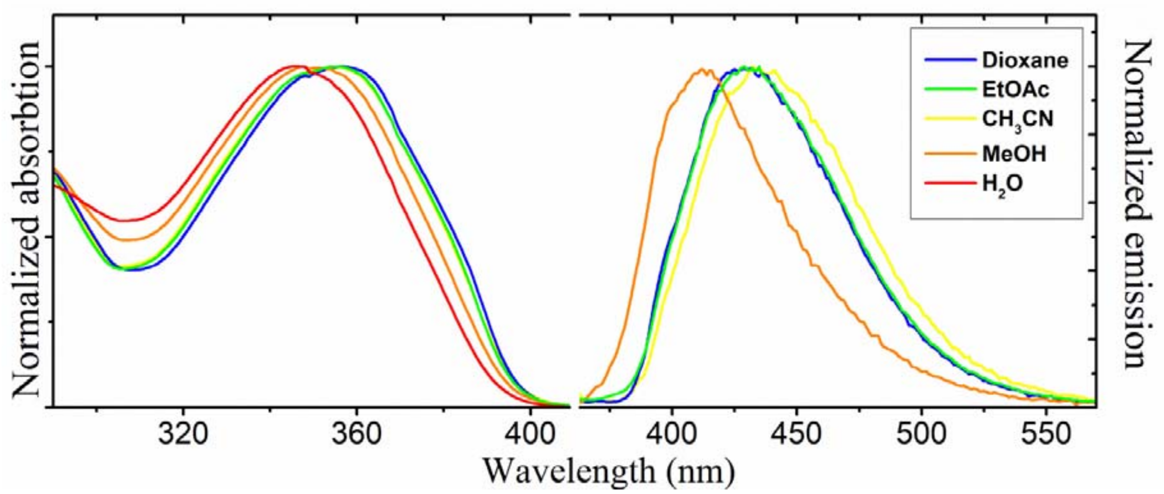

Fig. S1. Fluorescence and absorption spectra of **1a** in various solvents.

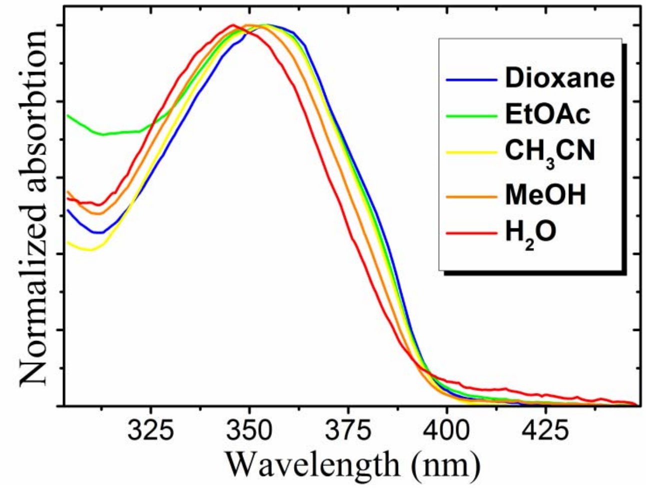

Fig. S2. Absorption spectra of **1b** in various solvents.

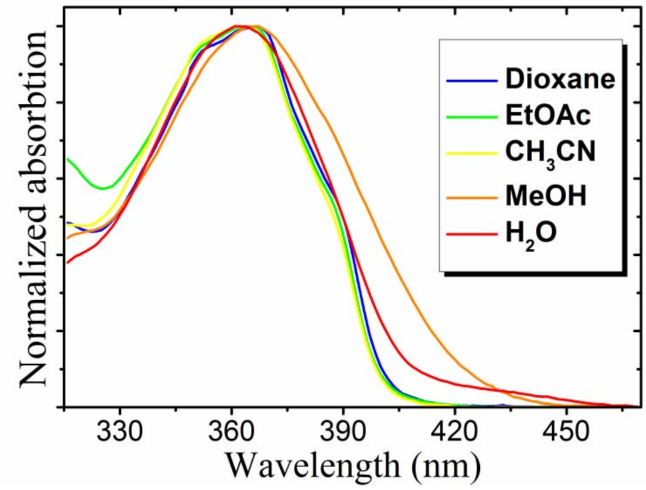

Fig. S3. Absorption spectra of **1c** in various solvents.

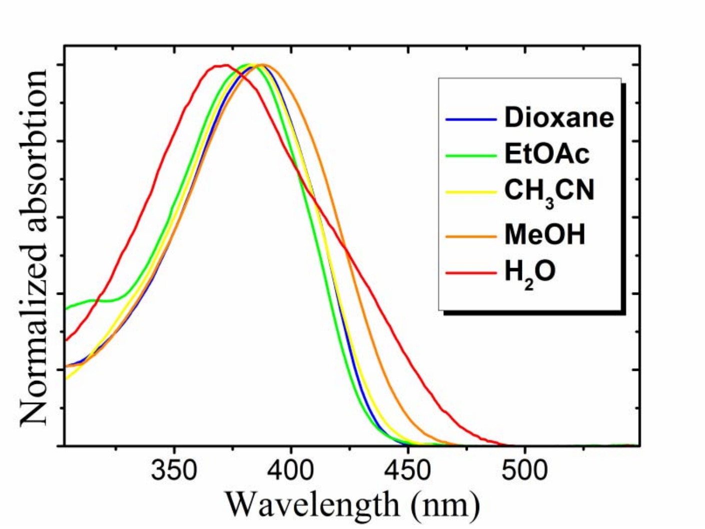

Fig. S4. Absorption spectra of **1d** in various solvents.

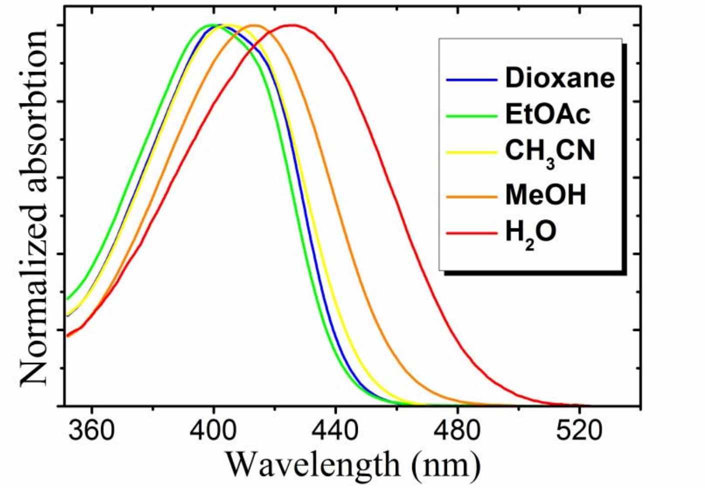

Fig. S5. Absorption spectra of **1e** in various solvents.

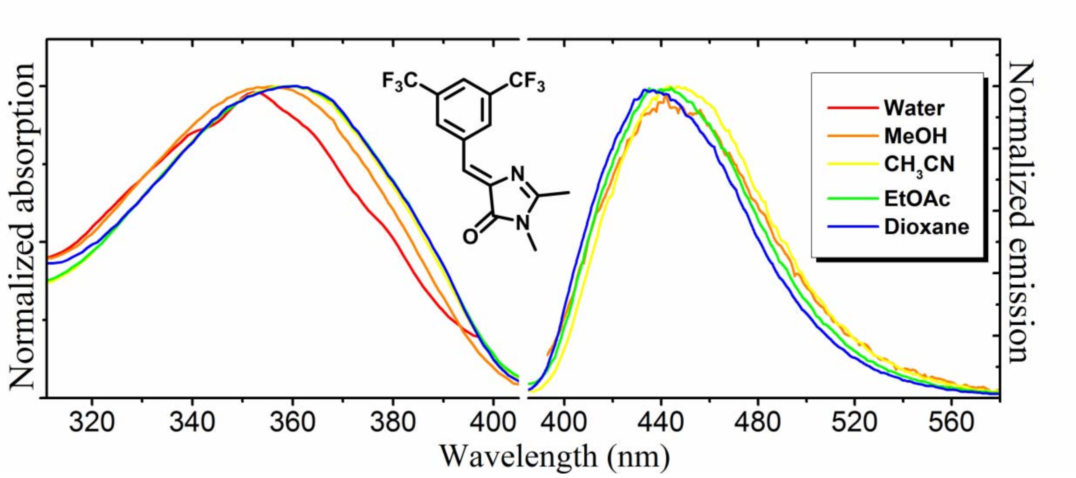

Fig. S6. Fluorescence and absorption spectra of **1f** in various solvents.

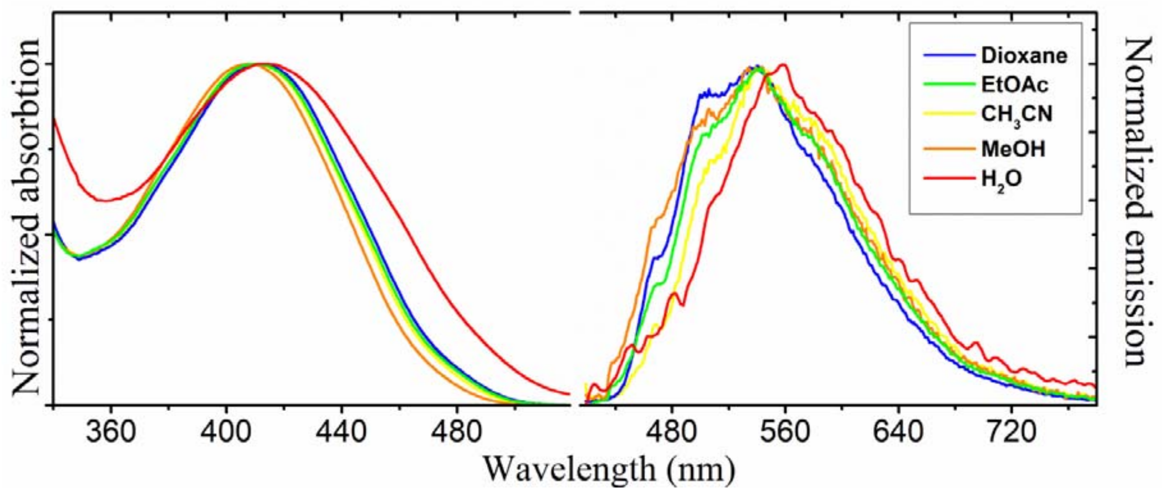

Fig. S7. Fluorescence and absorption spectra of **2a** in various solvents.

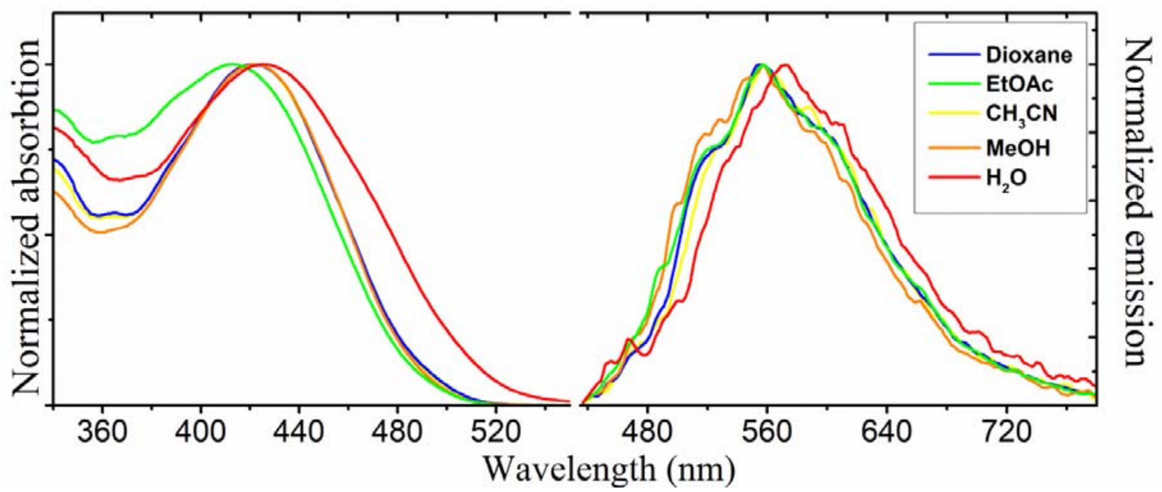

Fig. S8. Fluorescence and absorption spectra of **2b** in various solvents.

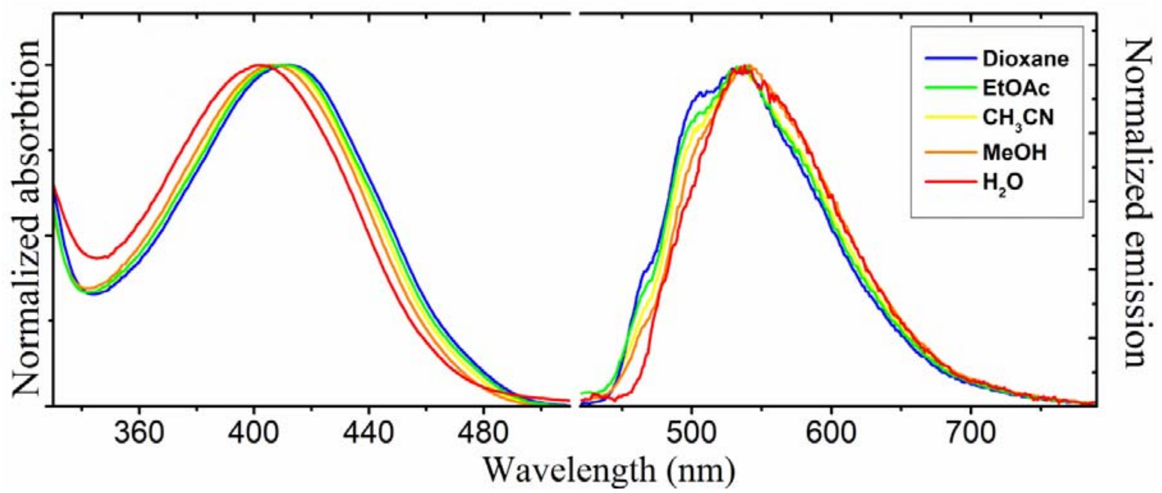

Fig. S9. Fluorescence and absorption spectra of **2c** in various solvents.

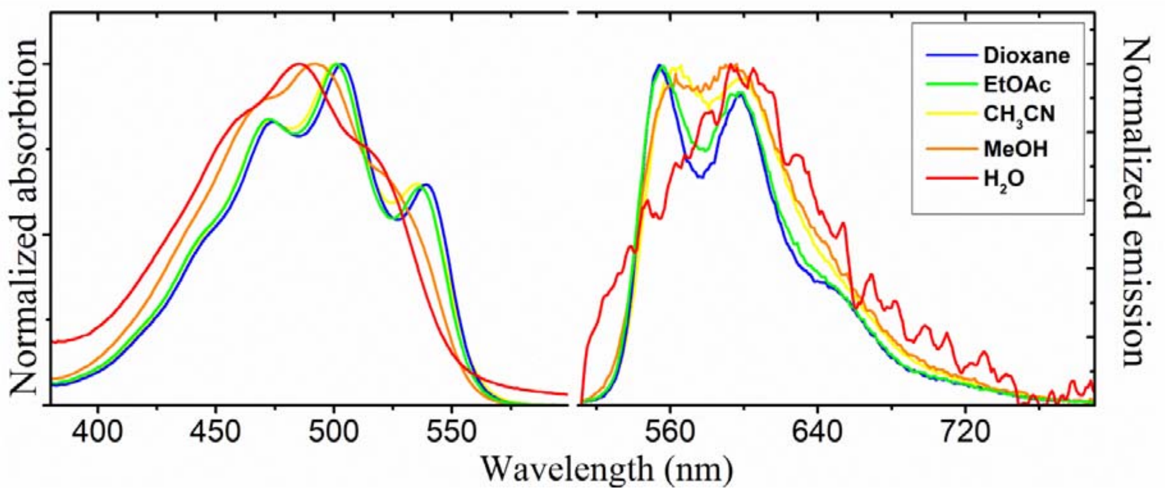

Fig. S10. Fluorescence and absorption spectra of **3** in various solvents.

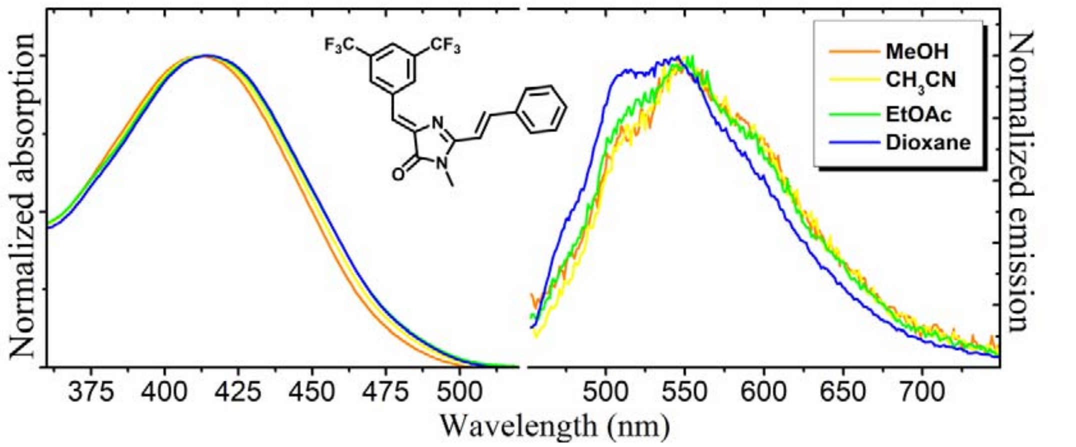

Figure S11. Fluorescence and absorption spectra of **2d** in various solvents.

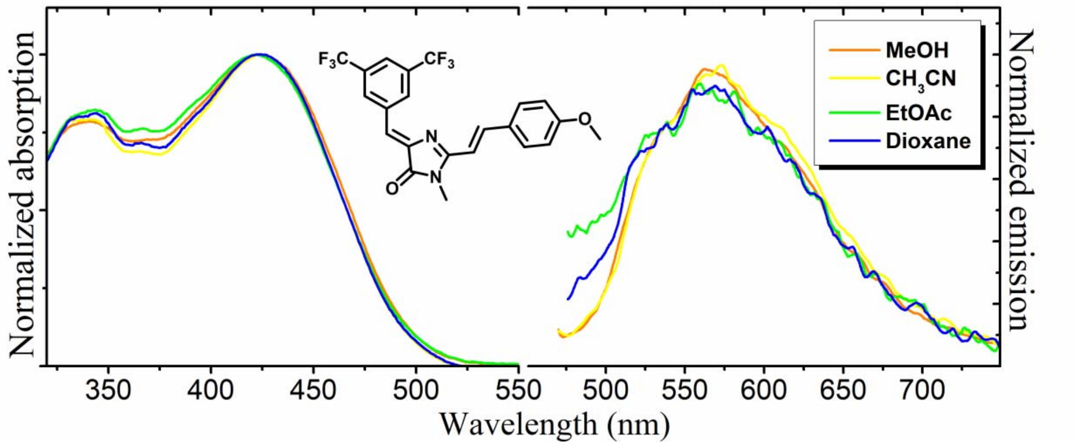

Figure S12. Fluorescence and absorption spectra of **2e** in various solvents.

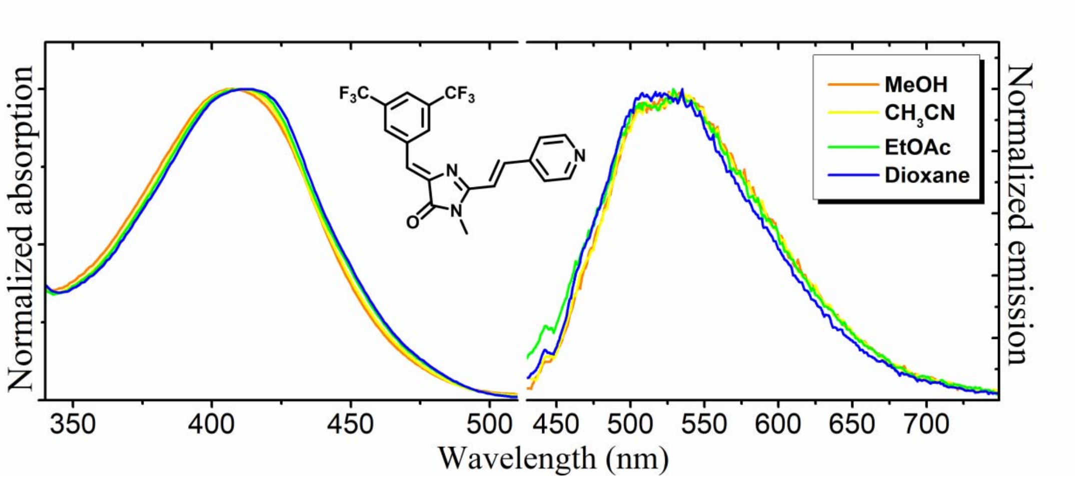

Figure S13. Fluorescence and absorption spectra of **2f** in various solvents.

3. Solid-state and aggregation-induced emission

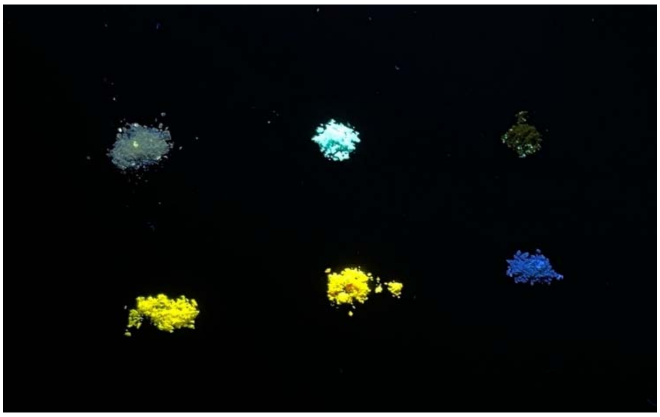

Figure S14. Photograph of samples of compounds **1** in solid form upon irradiation with 365 nm light. Top – **1a**, **1b**, and **1c**. Bottom –**1d**, **1e** and **1f**.

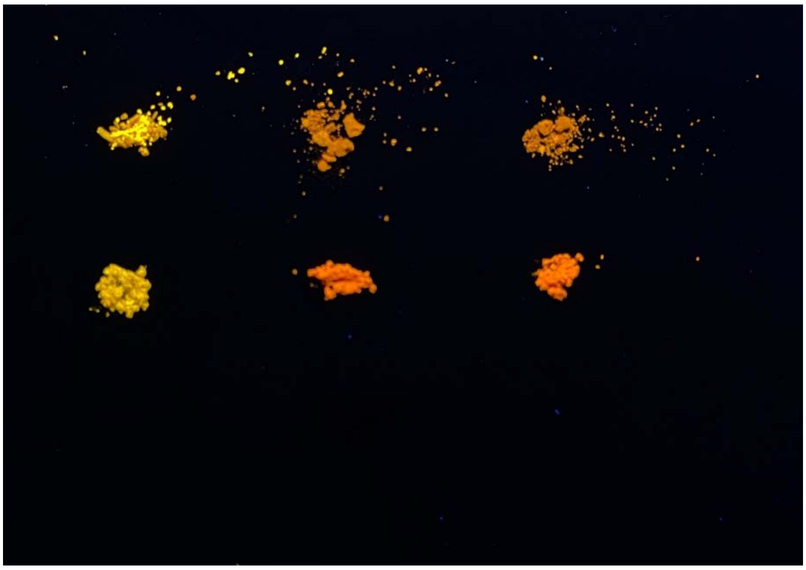

Figure S15. Photograph of samples of compounds **2** and **3** in solid form upon irradiation with 365 nm light. Top – **2a**, **2b**, and **2c**. Middle – **2e**, **2f**, and **2g**. Bottom – **3** (non fluorescent).

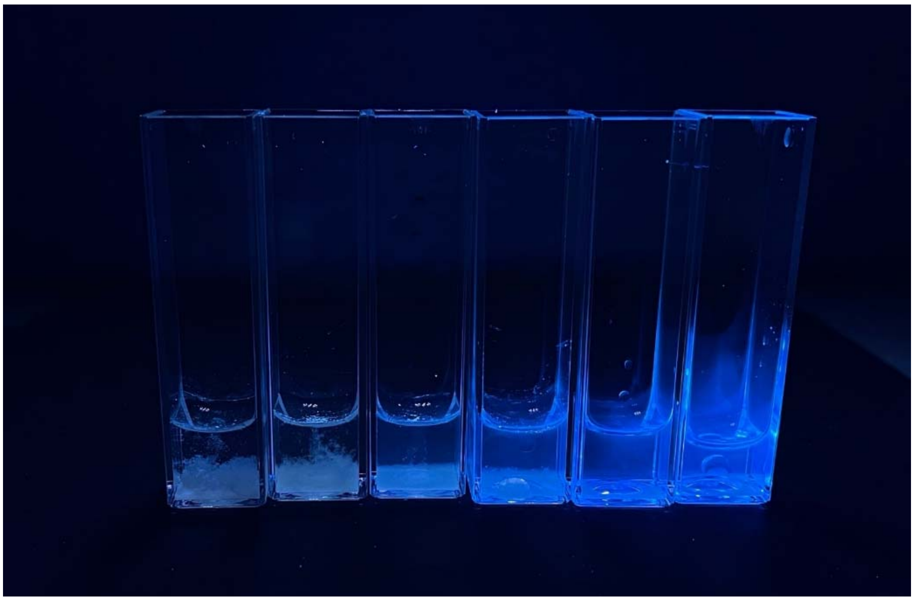

Figure S16. Photograph of compound **1a** dripped from DMSO stock solution to various acetone–water mixtures containing different volume fractions of water upon irradiation with 365 nm light. From left to right – 100, 90, 80, 70, 60 and 50% of water.

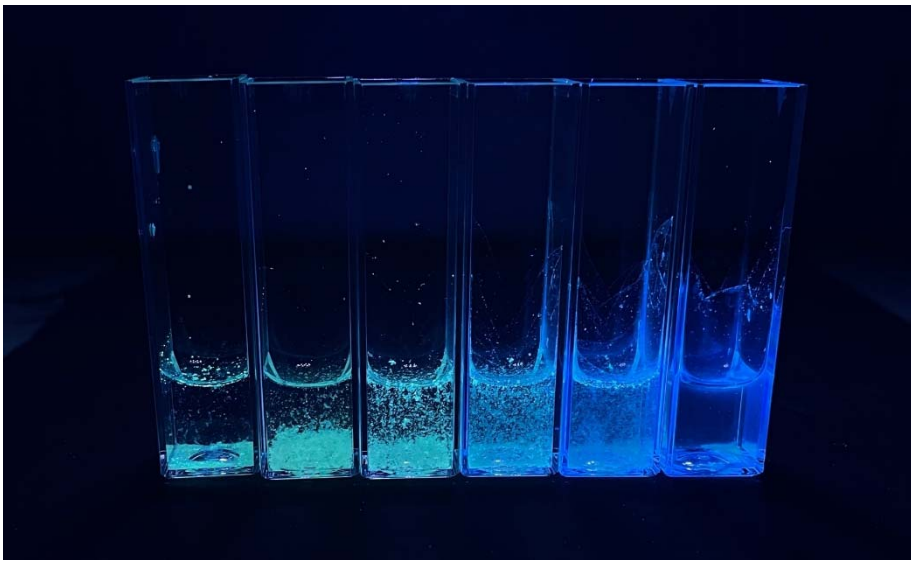

Figure S17. Photograph of compound **1b** dripped from DMSO stock solution to various acetone–water mixtures containing different volume fractions of water upon irradiation with 365 nm light. From left to right – 100, 90, 80, 70, 60 and 50% of water.

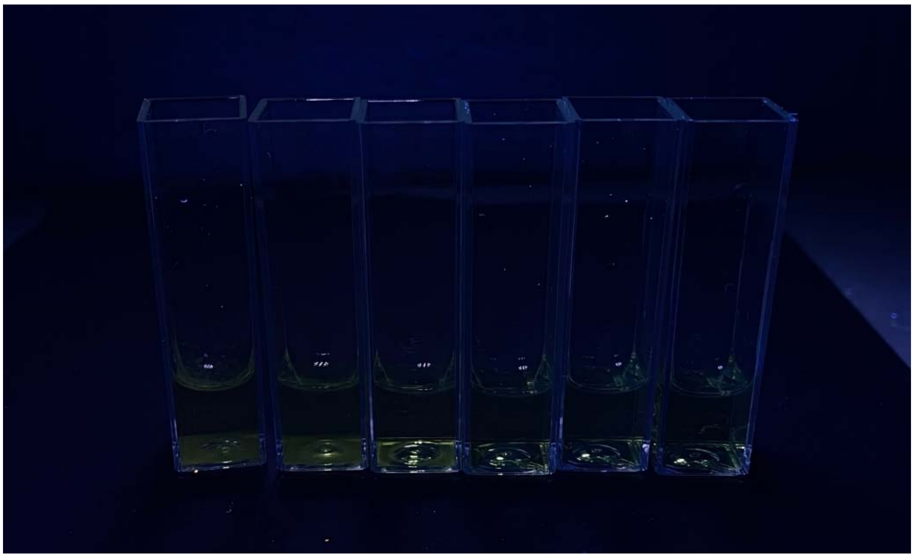

Figure S18. Photograph of compound **1c** dripped from DMSO stock solution to various acetone–water mixtures containing different volume fractions of water upon irradiation with 365 nm light. From left to right – 100, 90, 80, 70, 60 and 50% of water.

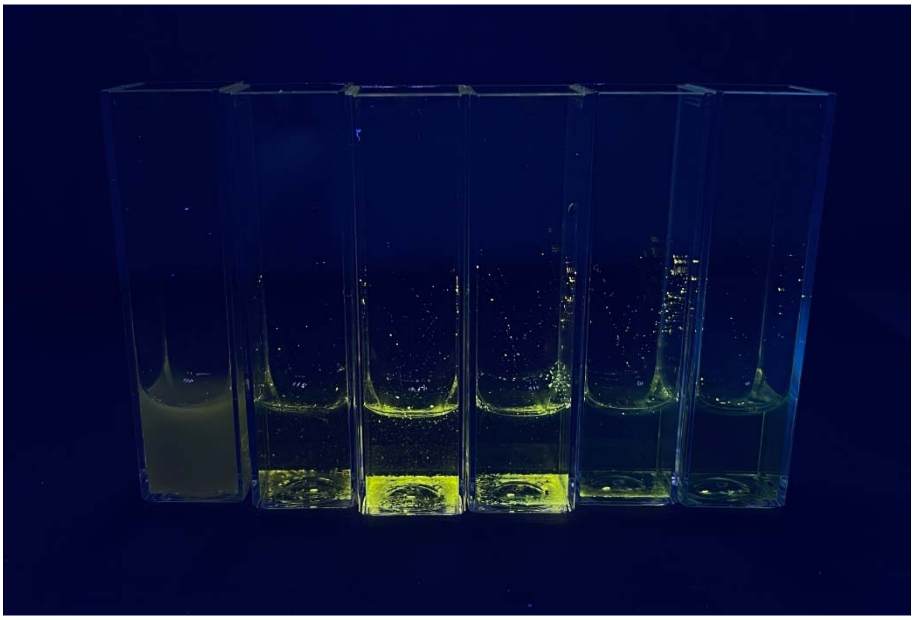

Figure S19. Photograph of compound **1d** dripped from DMSO stock solution to various acetone–water mixtures containing different volume fractions of water upon irradiation with 365 nm light. From left to right – 100, 90, 80, 70, 60 and 50% of water.

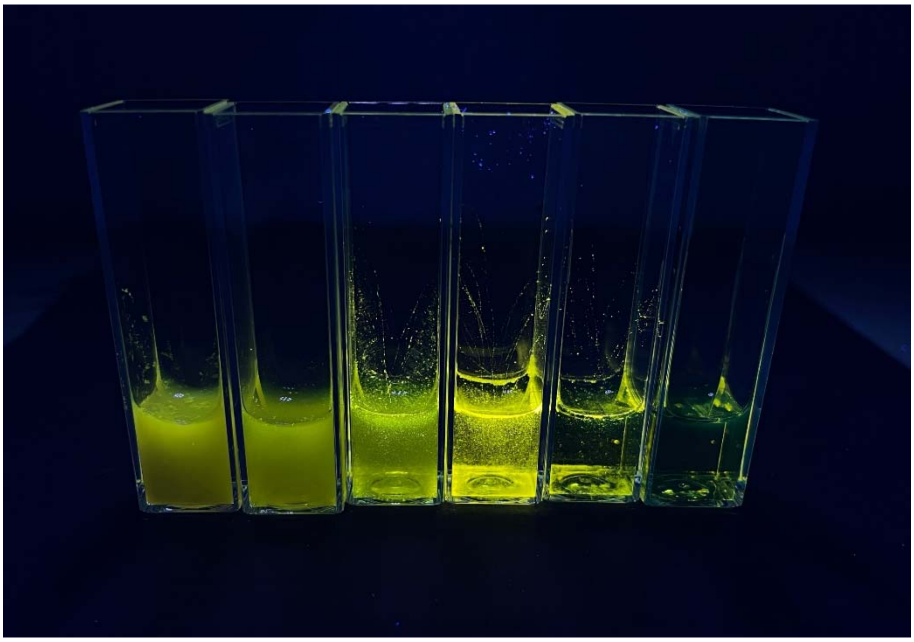

Figure S20. Photograph of compound **1e** dripped from DMSO stock solution to various acetone–water mixtures containing different volume fractions of water upon irradiation with 365 nm light. From left to right – 100, 90, 80, 70, 60 and 50% of water.

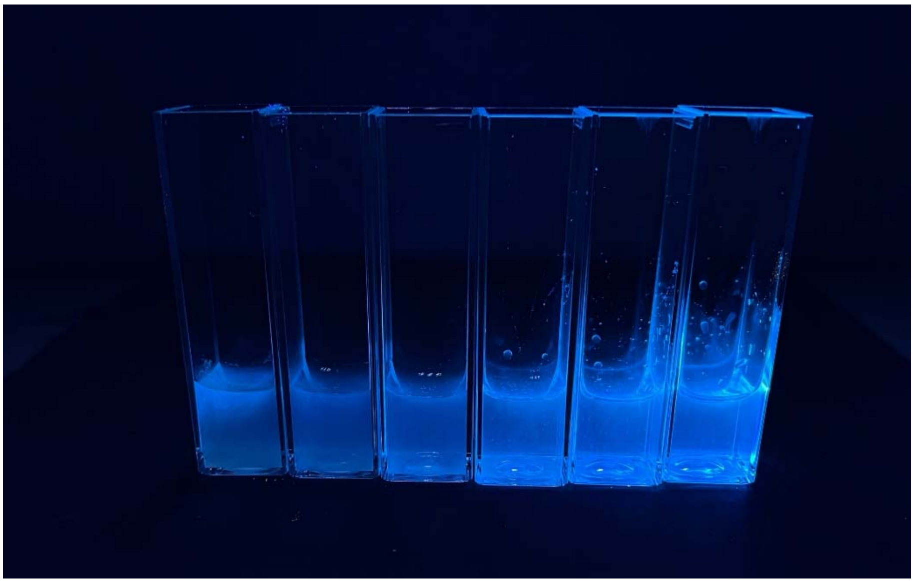

Figure S21. Photograph of compound **1f** dripped from DMSO stock solution to various acetone–water mixtures containing different volume fractions of water upon irradiation with 365 nm light. From left to right – 100, 90, 80, 70, 60 and 50% of water.

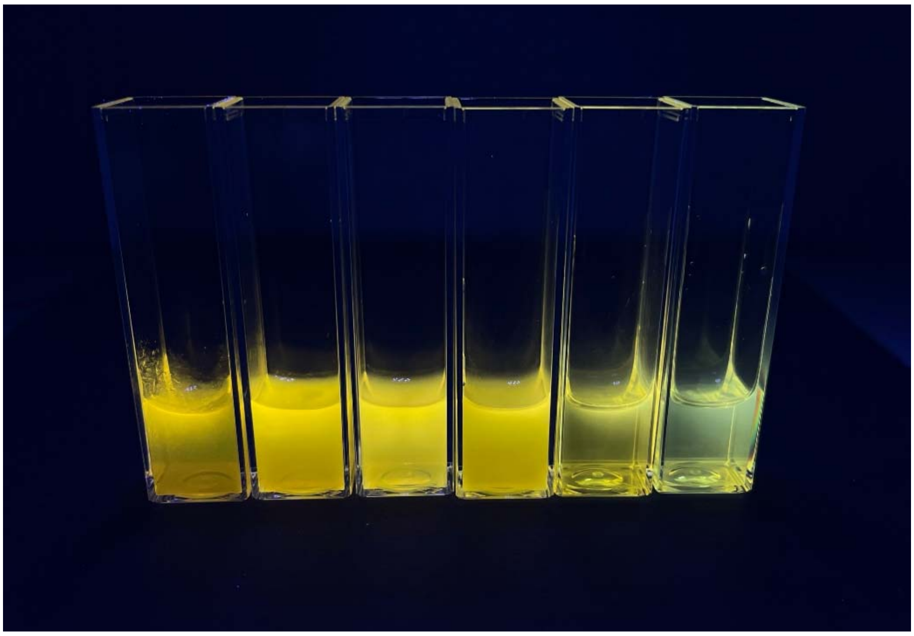

Figure S22. Photograph of compound **2a** dripped from DMSO stock solution to various acetone–water mixtures containing different volume fractions of water upon irradiation with 365 nm light. From left to right – 100, 90, 80, 70, 60 and 50% of water.

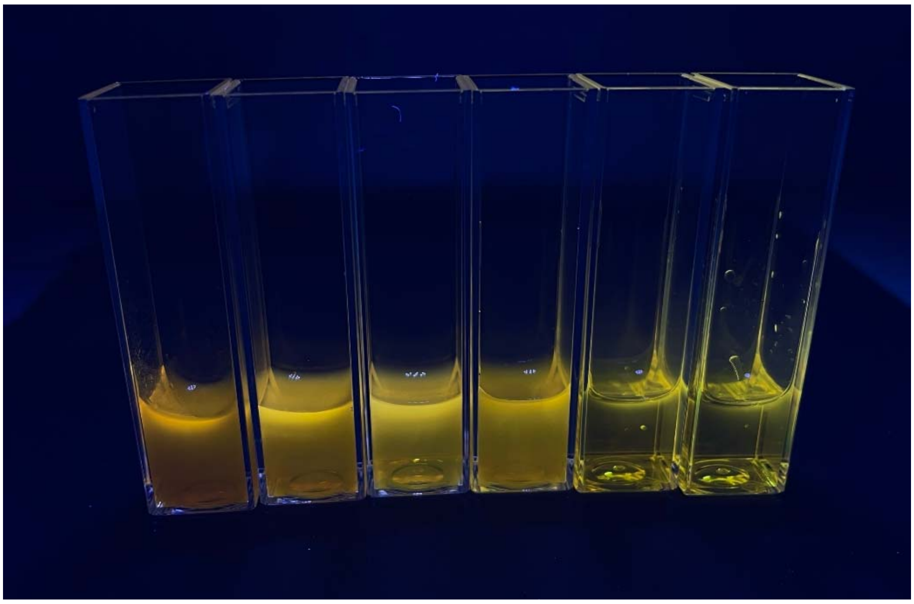

Figure S23. Photograph of compound **2b** dripped from DMSO stock solution to various acetone–water mixtures containing different volume fractions of water upon irradiation with 365 nm light. From left to right – 100, 90, 80, 70, 60 and 50% of water.

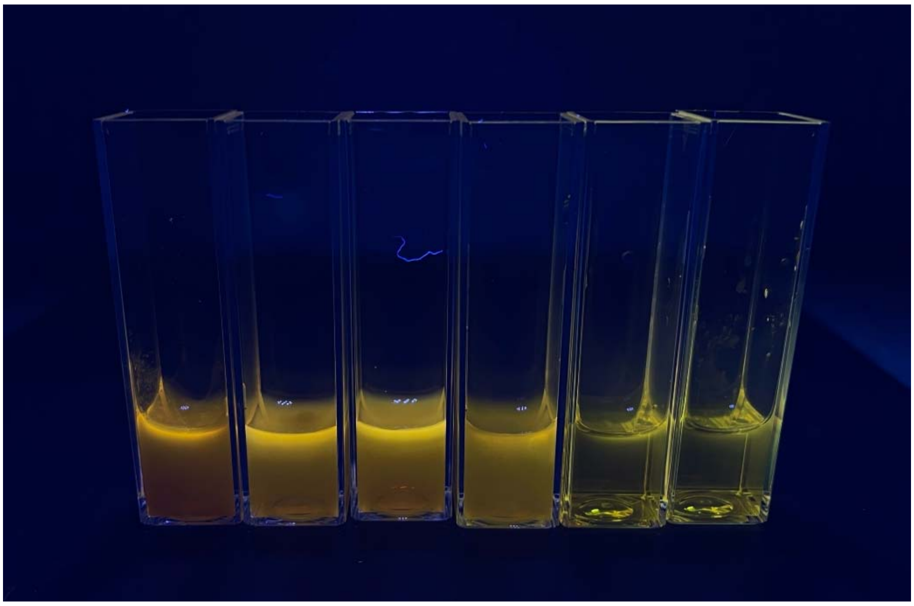

Figure S24. Photograph of compound **2c** dripped from DMSO stock solution to various acetone–water mixtures containing different volume fractions of water upon irradiation with 365 nm light. From left to right – 100, 90, 80, 70, 60 and 50% of water.

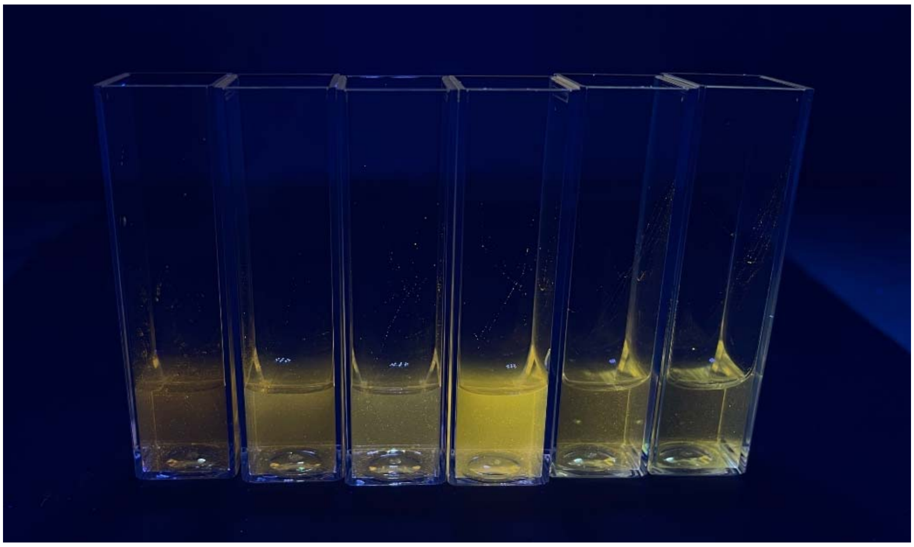

Figure S25. Photograph of compound **2d** dripped from DMSO stock solution to various acetone–water mixtures containing different volume fractions of water upon irradiation with 365 nm light. From left to right – 100, 90, 80, 70, 60 and 50% of water.

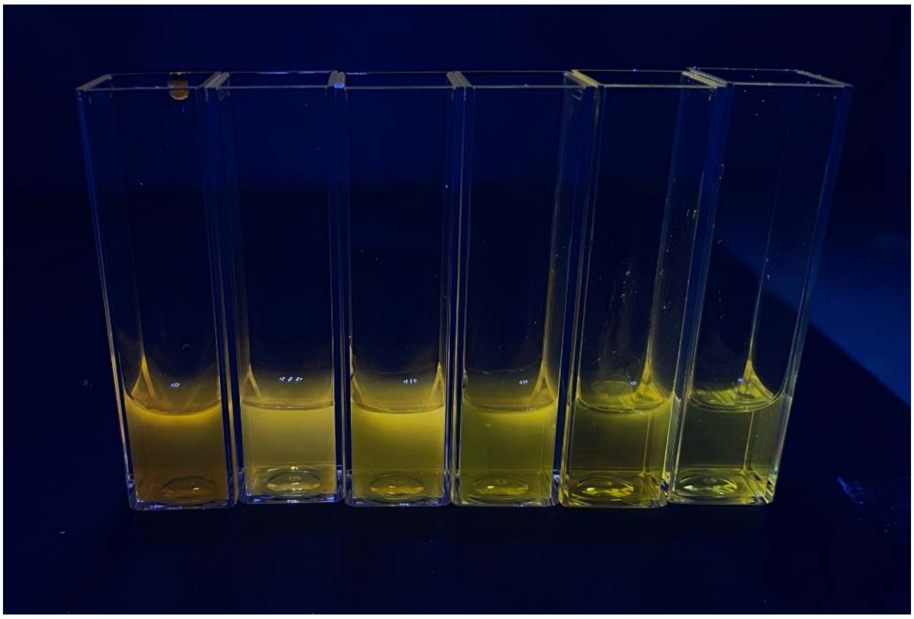

Figure S26. Photograph of compound **2e** dripped from DMSO stock solution to various acetone–water mixtures containing different volume fractions of water upon irradiation with 365 nm light. From left to right – 100, 90, 80, 70, 60 and 50% of water.

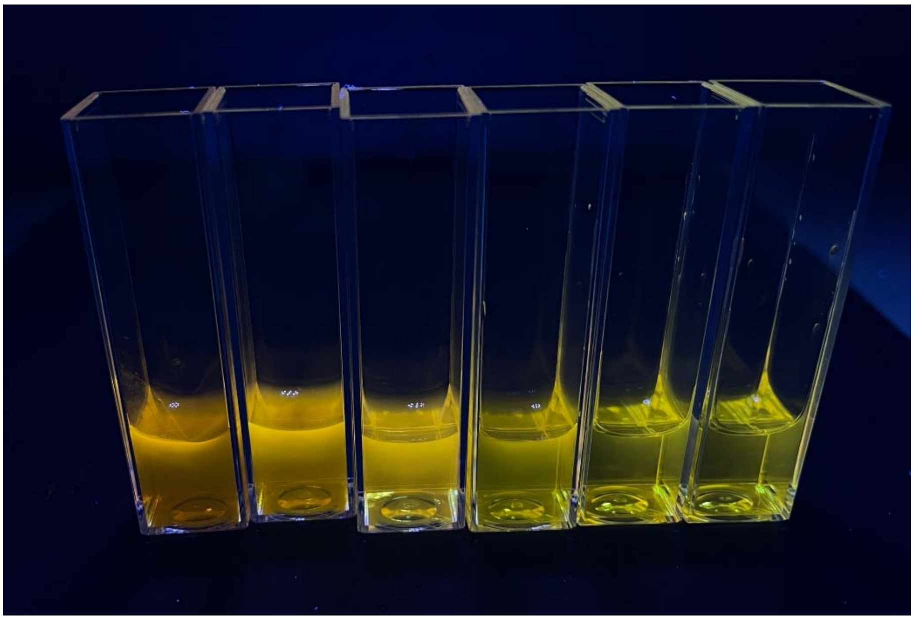

Figure S27. Photograph of compound **2f** dripped from DMSO stock solution to various acetone–water mixtures containing different volume fractions of water upon irradiation with 365 nm light. From left to right – 100, 90, 80, 70, 60 and 50% of water.

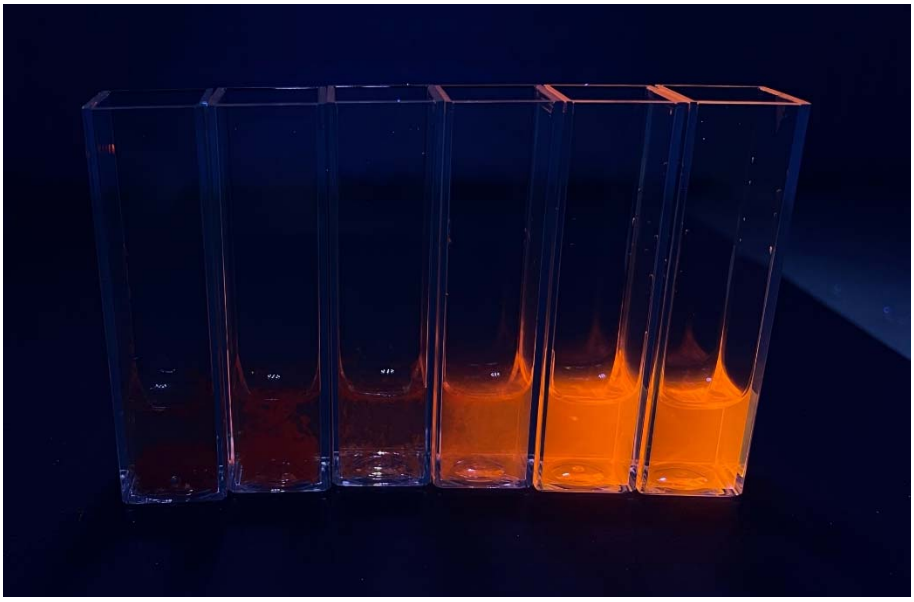

Figure S28. Photograph of compound **3** dripped from DMSO stock solution to various acetone–water mixtures containing different volume fractions of water upon irradiation with 365 nm light. From left to right – 100, 90, 80, 70, 60 and 50% of water.

4. Copies of ^{1}H and ^{13}C NMR spectra

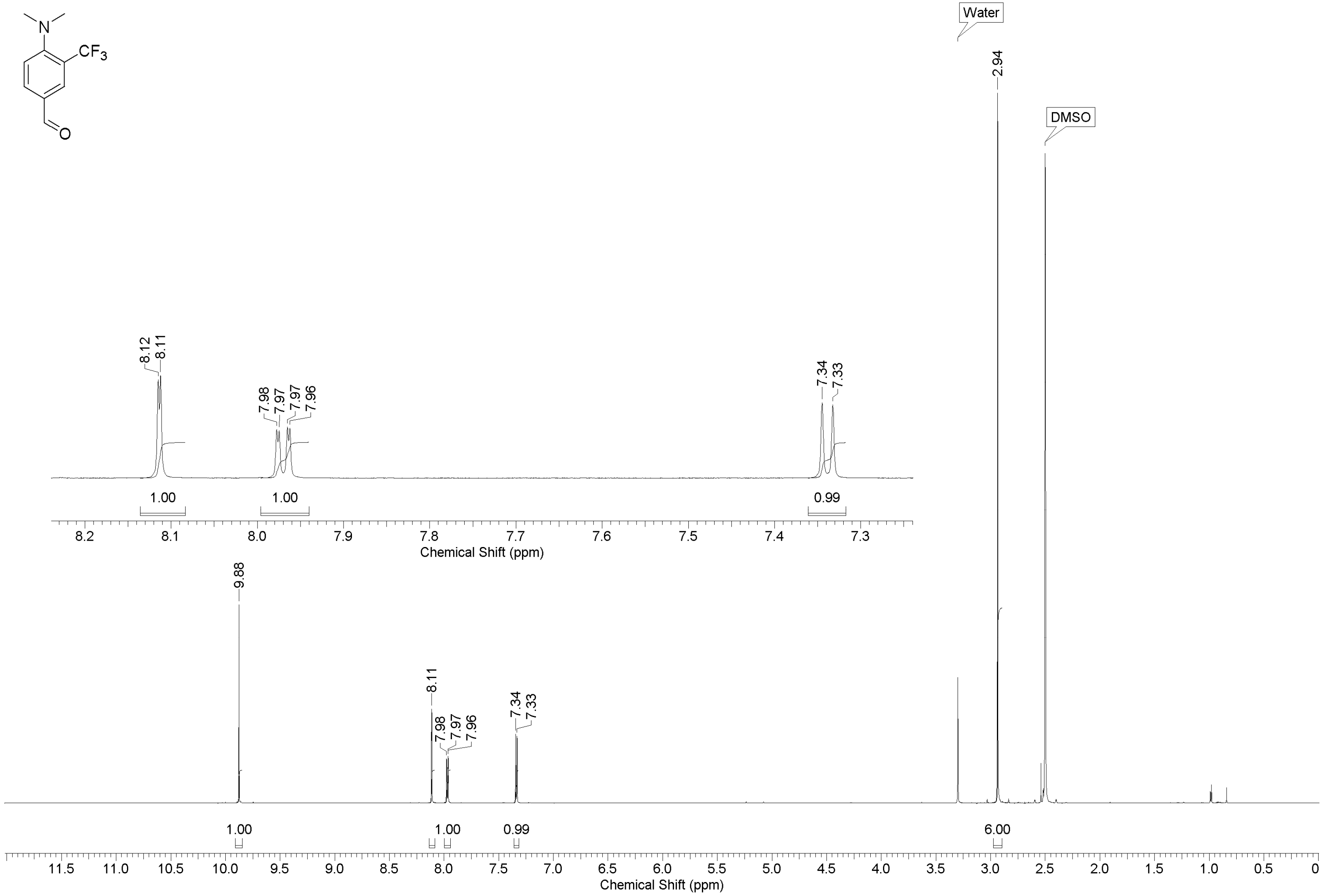

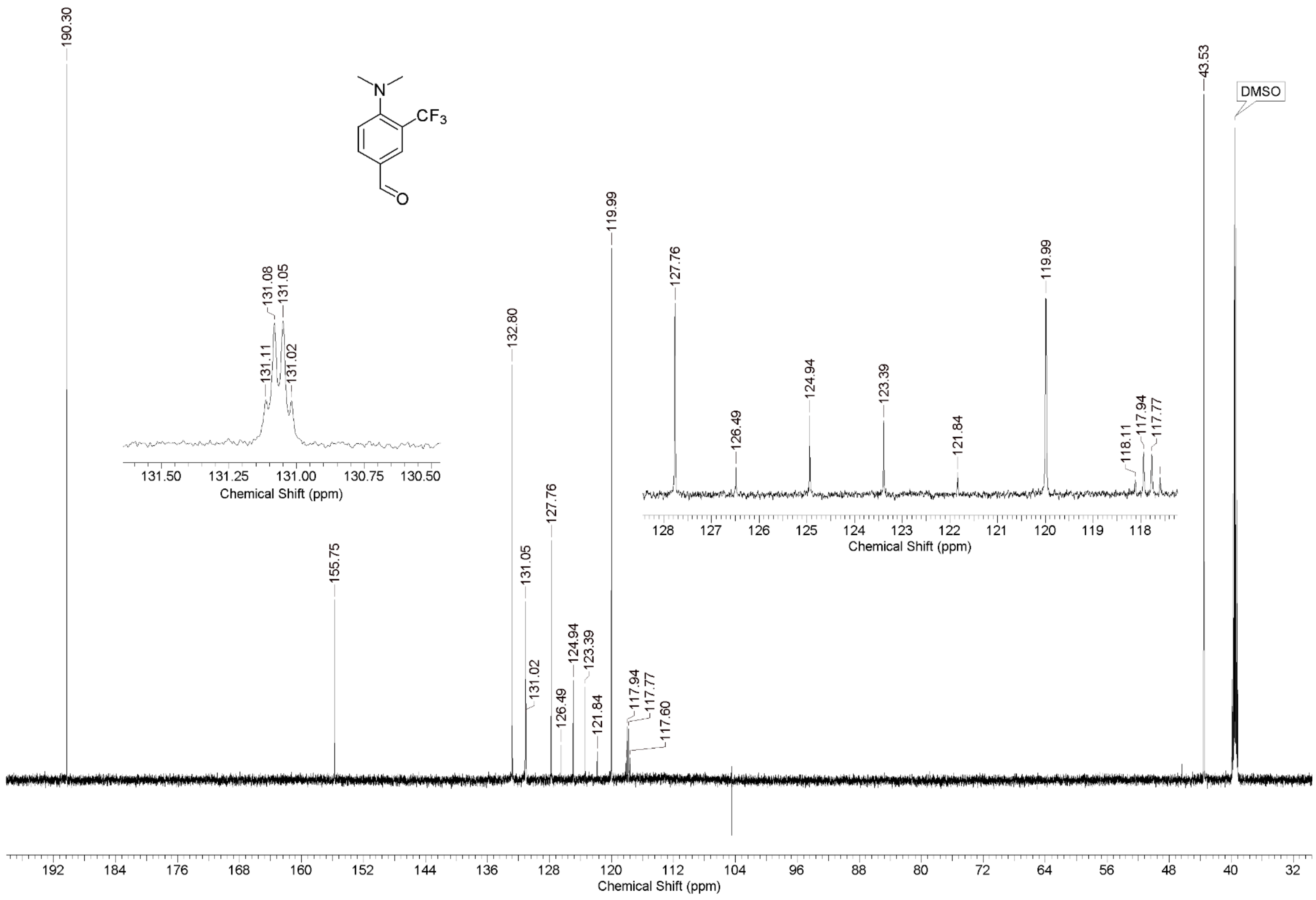
190.30
155.75
132.80
131.05
131.02
127.76
126.49
124.94
123.39
121.84
119.99
117.94
117.77
117.60
43.53
DMSO
CF3
N
O
131.11
131.08
131.05
131.02
131.50 131.25 131.00 130.75 130.50
Chemical Shift (ppm)
127.76
126.49
124.94
123.39
121.84
119.99
118.11
117.94
117.77
128 127 126 125 124 123 122 121 120 119 118
Chemical Shift (ppm)
192 184 176 168 160 152 144 136 128 120 112 104 96 88 80 72 64 56 48 40 32
Chemical Shift (ppm)

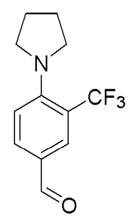
N
CF3
O

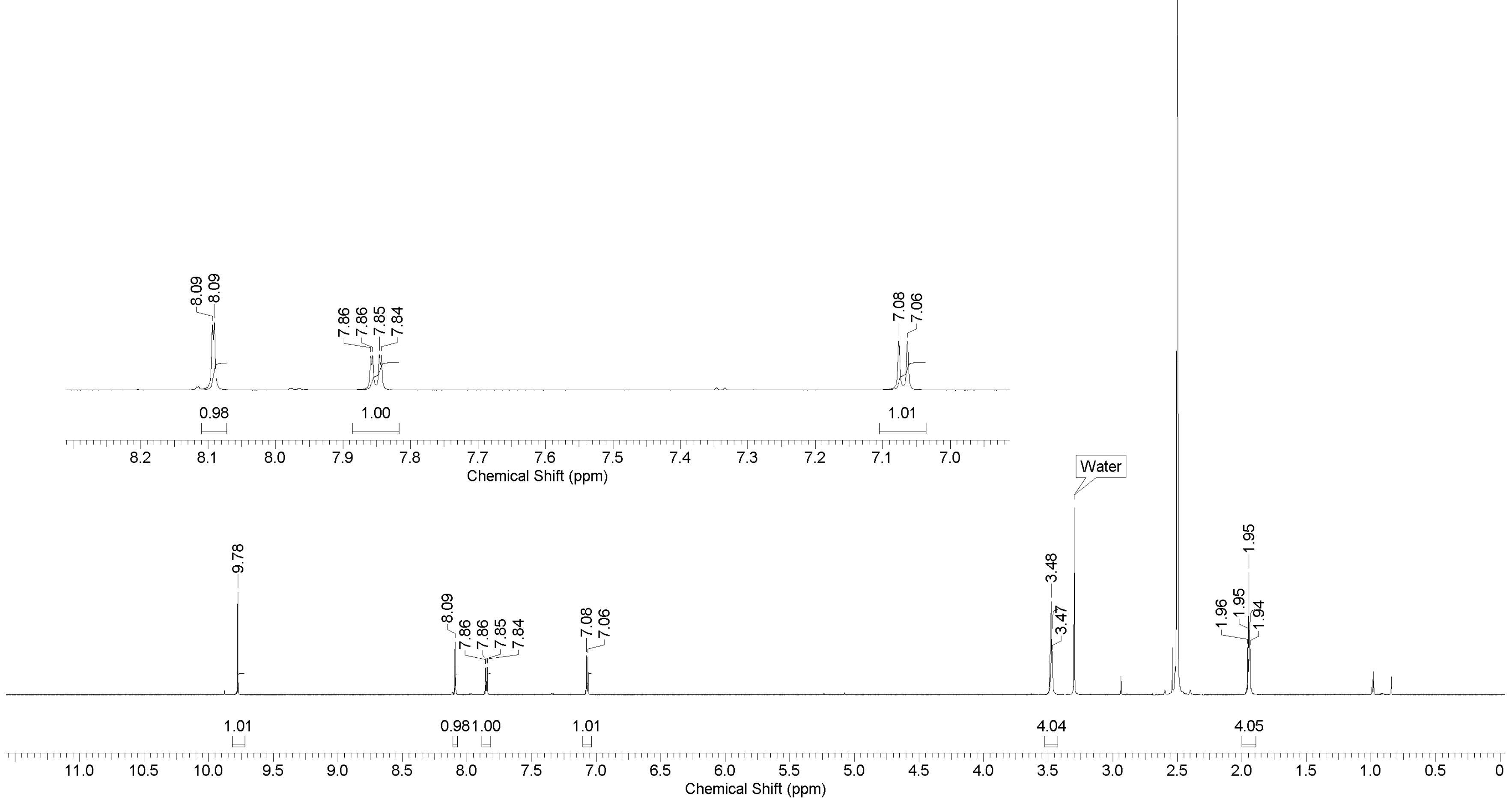
DMSO
Water
9.78
8.09
7.86
7.86
7.85
7.84
7.08
7.06
3.48
3.47
1.96
1.95
1.95
1.94
1.01
0.98
1.00
1.01
4.04
4.05
Chemical Shift (ppm)

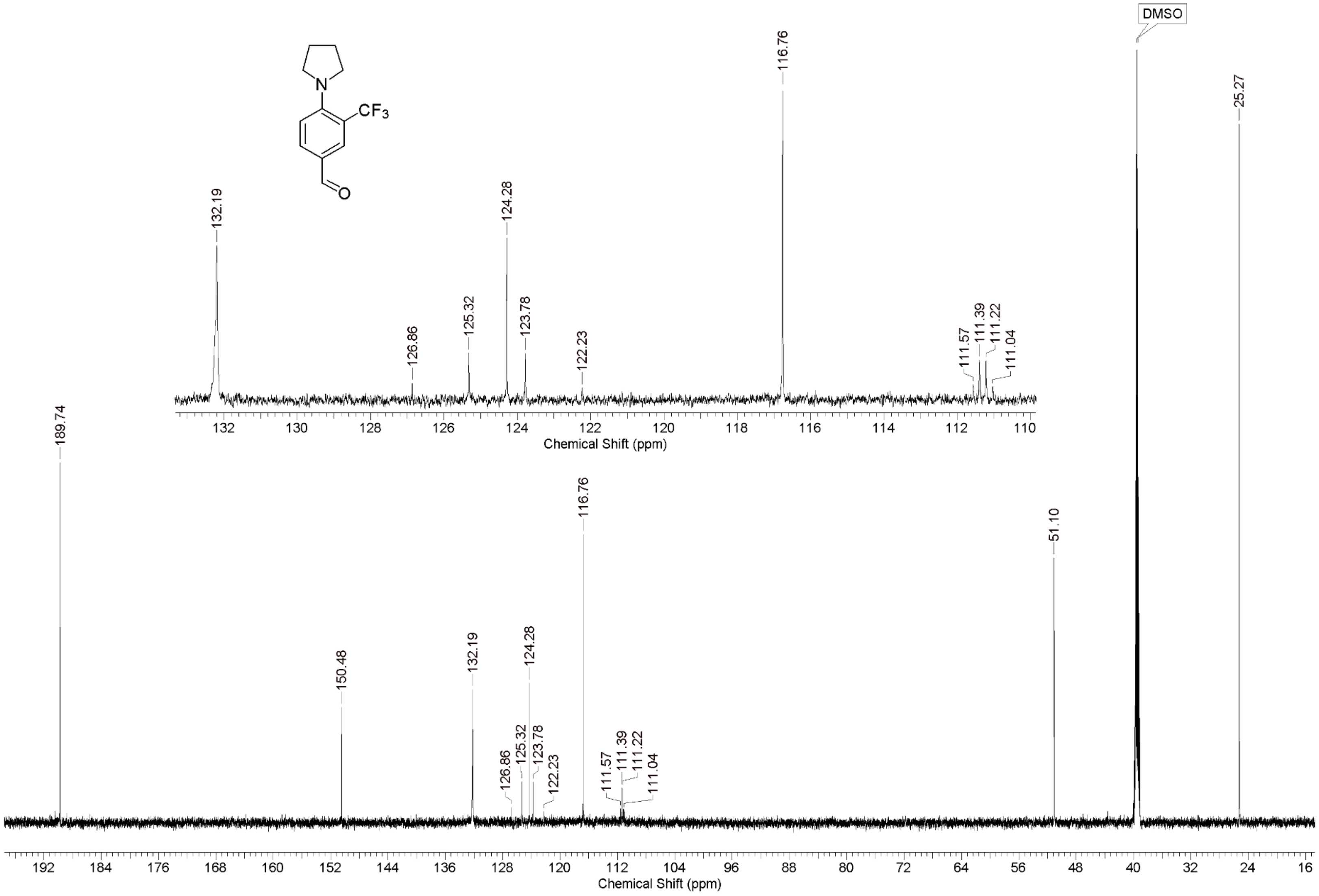
N
CF3
O
DMSO
189.74
150.48
132.19
126.86
125.32
124.28
123.78
122.23
116.76
111.57
111.39
111.22
111.04
51.10
25.27
Chemical Shift (ppm)
Chemical Shift (ppm)

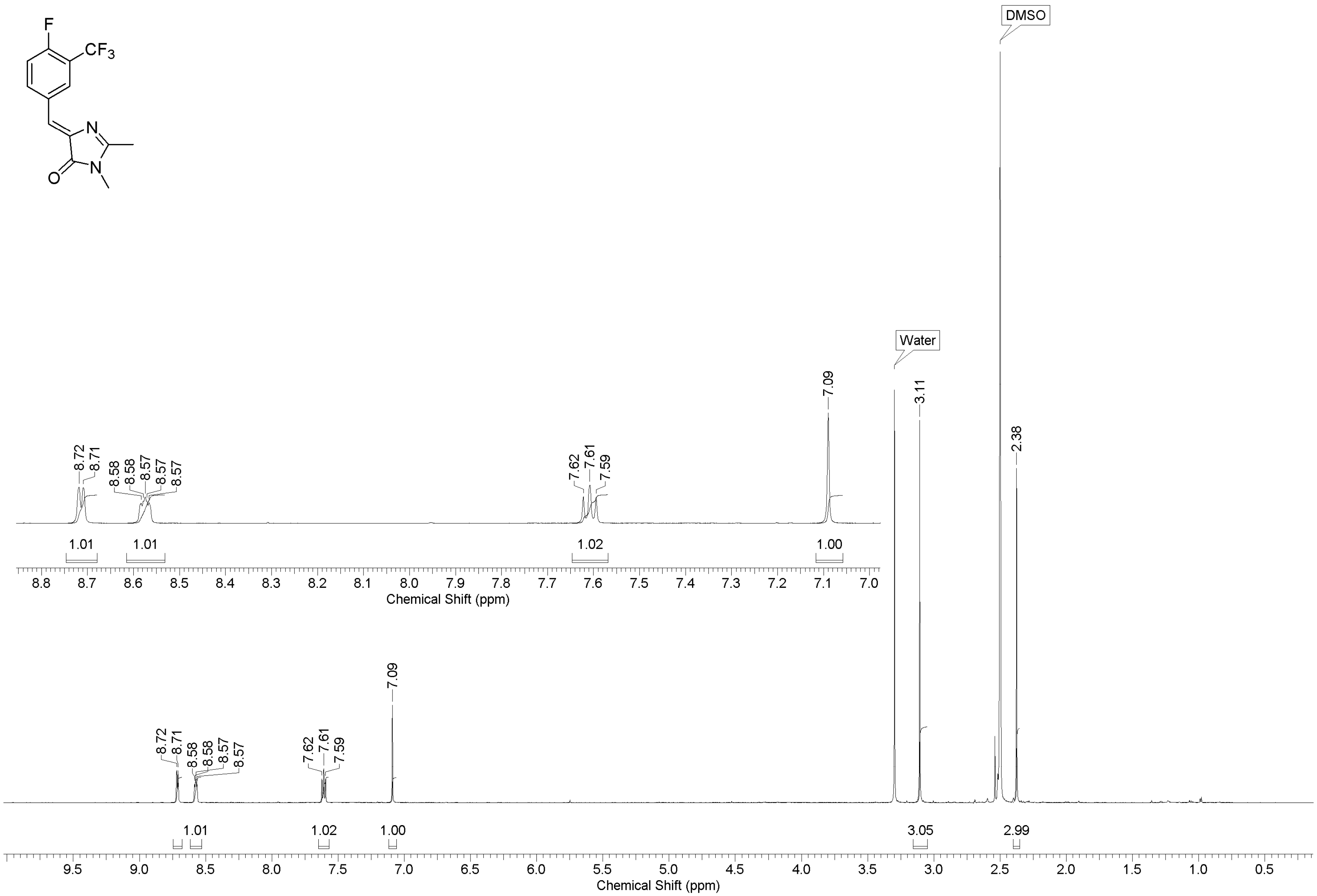
F
CF3
N
N
O
DMSO
Water
7.09
3.11
2.38
8.72
8.71
8.58
8.58
8.57
8.57
8.57
7.62
7.61
7.59
1.01
1.01
1.02
1.00
3.05
2.99
Chemical Shift (ppm)

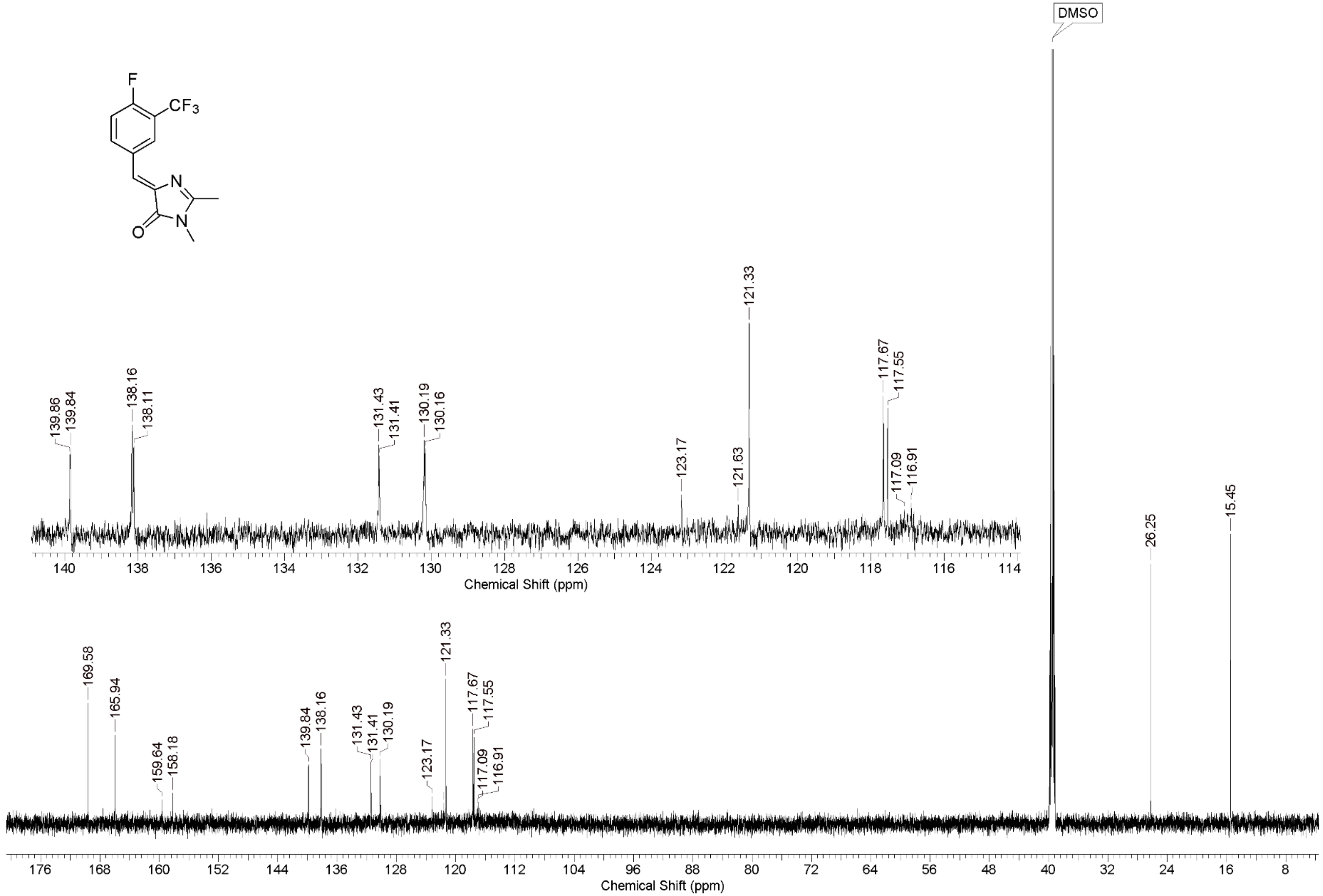
F
CF_3
N
N
O
DMSO
139.86
139.84
138.16
138.11
131.43
131.41
130.19
130.16
123.17
121.63
121.33
117.67
117.55
117.09
116.91
140 138 136 134 132 130 128 126 124 122 120 118 116 114
Chemical Shift (ppm)
169.58
165.94
159.64
158.18
139.84
138.16
131.43
131.41
130.19
123.17
121.33
117.67
117.55
117.09
116.91
26.25
15.45
176 168 160 152 144 136 128 120 112 104 96 88 80 72 64 56 48 40 32 24 16 8
Chemical Shift (ppm)

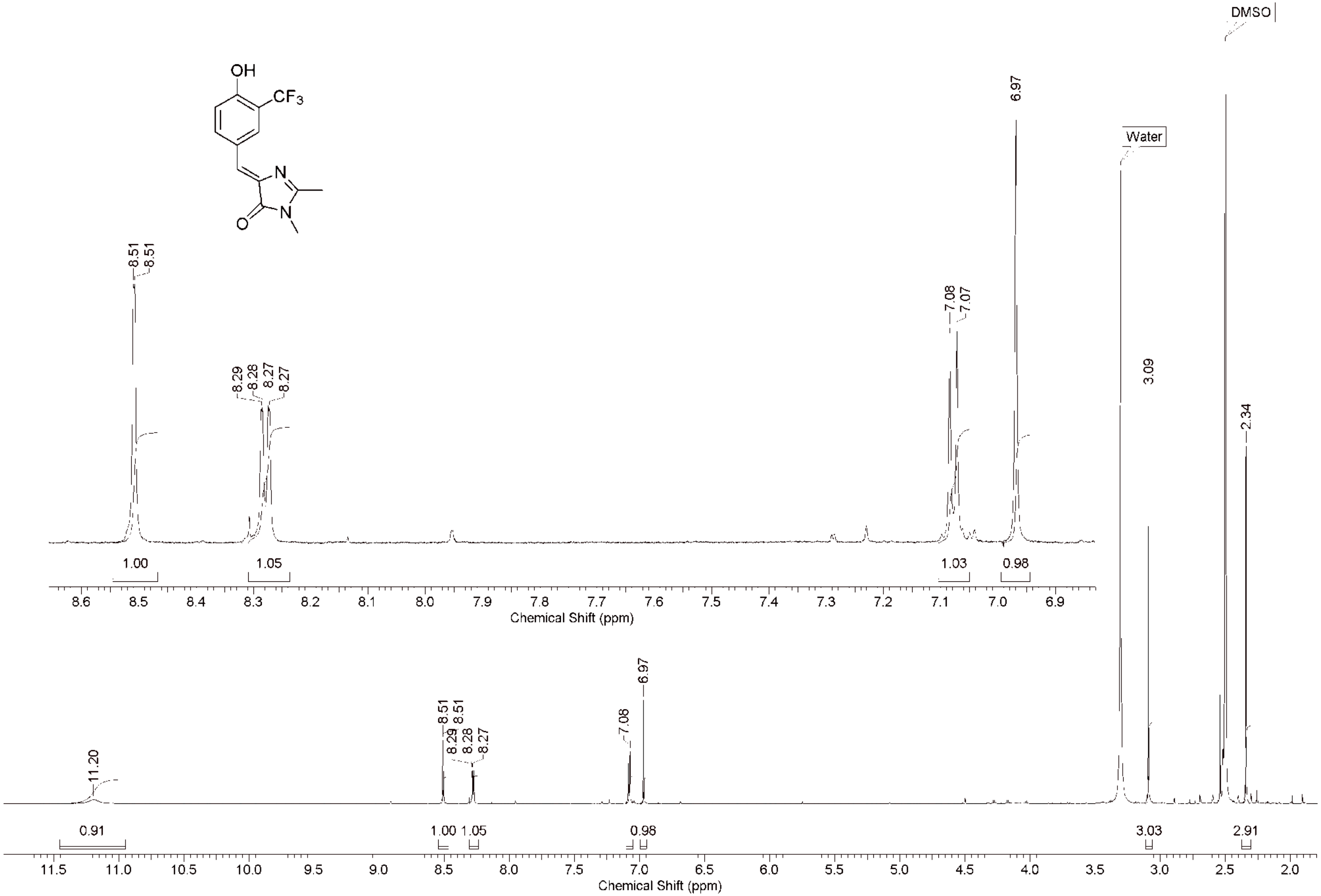
OH
CF3
N
N
O
DMSO
Water
8.51
8.51
8.29
8.28
8.27
8.27
7.08
7.07
6.97
3.09
2.34
11.20
1.00
1.05
1.03
0.98
0.91
3.03
2.91
Chemical Shift (ppm)
Chemical Shift (ppm)

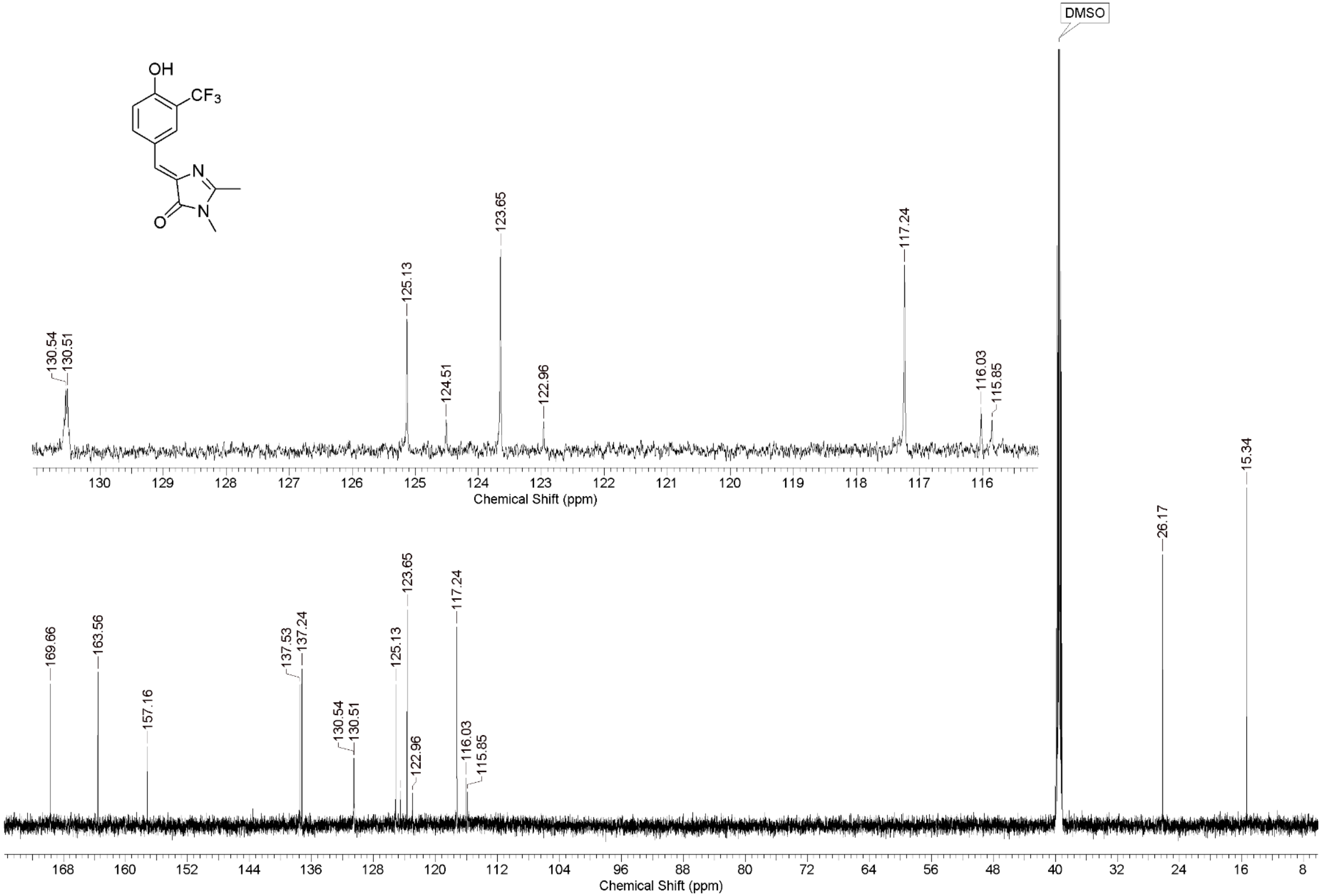
OH
CF3
N
O
DMSO
130.54
130.51
125.13
124.51
123.65
122.96
117.24
116.03
115.85
Chemical Shift (ppm)
169.66
163.56
157.16
137.53
137.24
130.54
130.51
125.13
123.65
122.96
117.24
116.03
115.85
26.17
15.34
Chemical Shift (ppm)

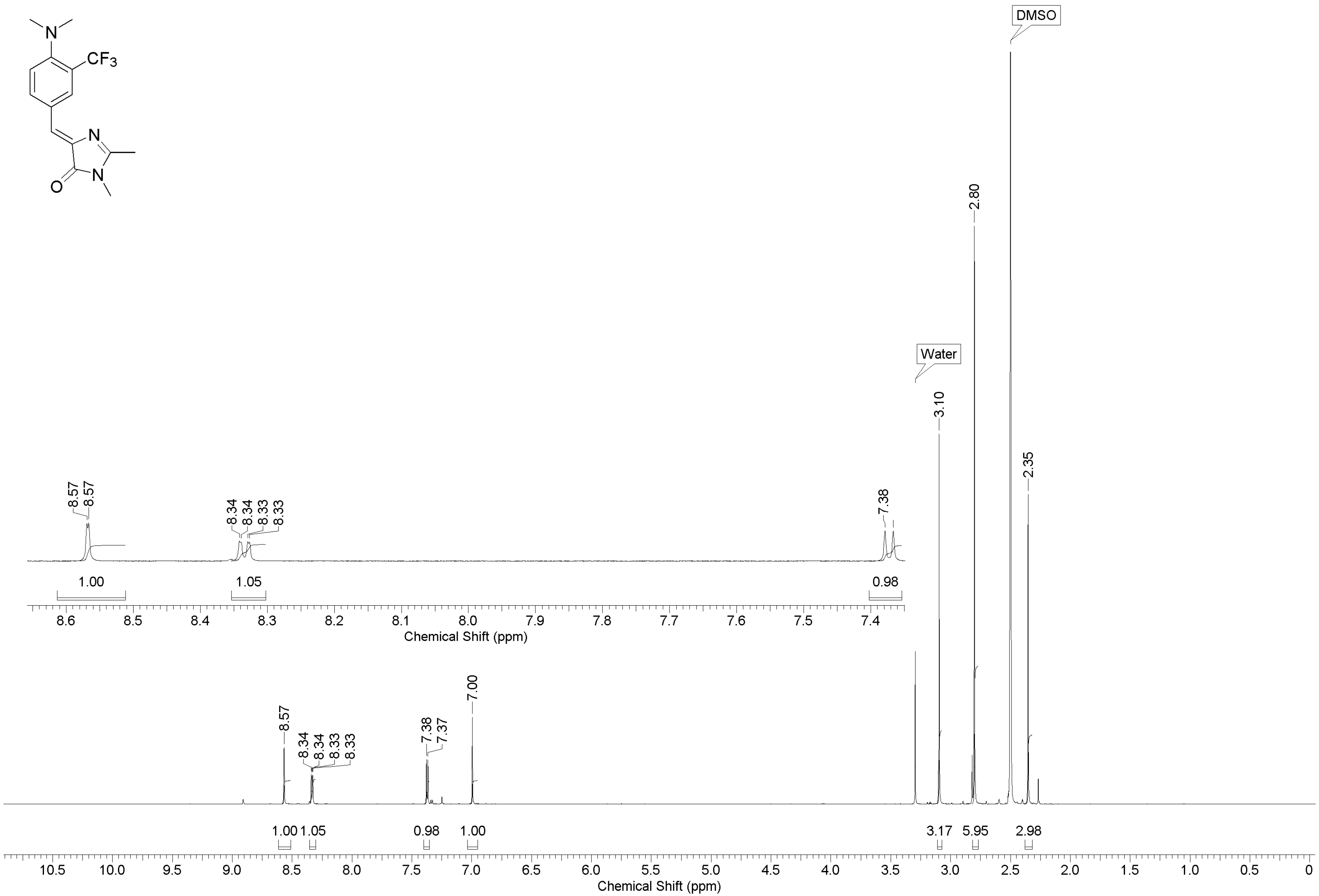

CF3
N
O
DMSO
Water
2.80
3.10
2.35
8.57
8.34
8.33
7.38
7.37
7.00
1.00
1.05
0.98
3.17
5.95
2.98
Chemical Shift (ppm)

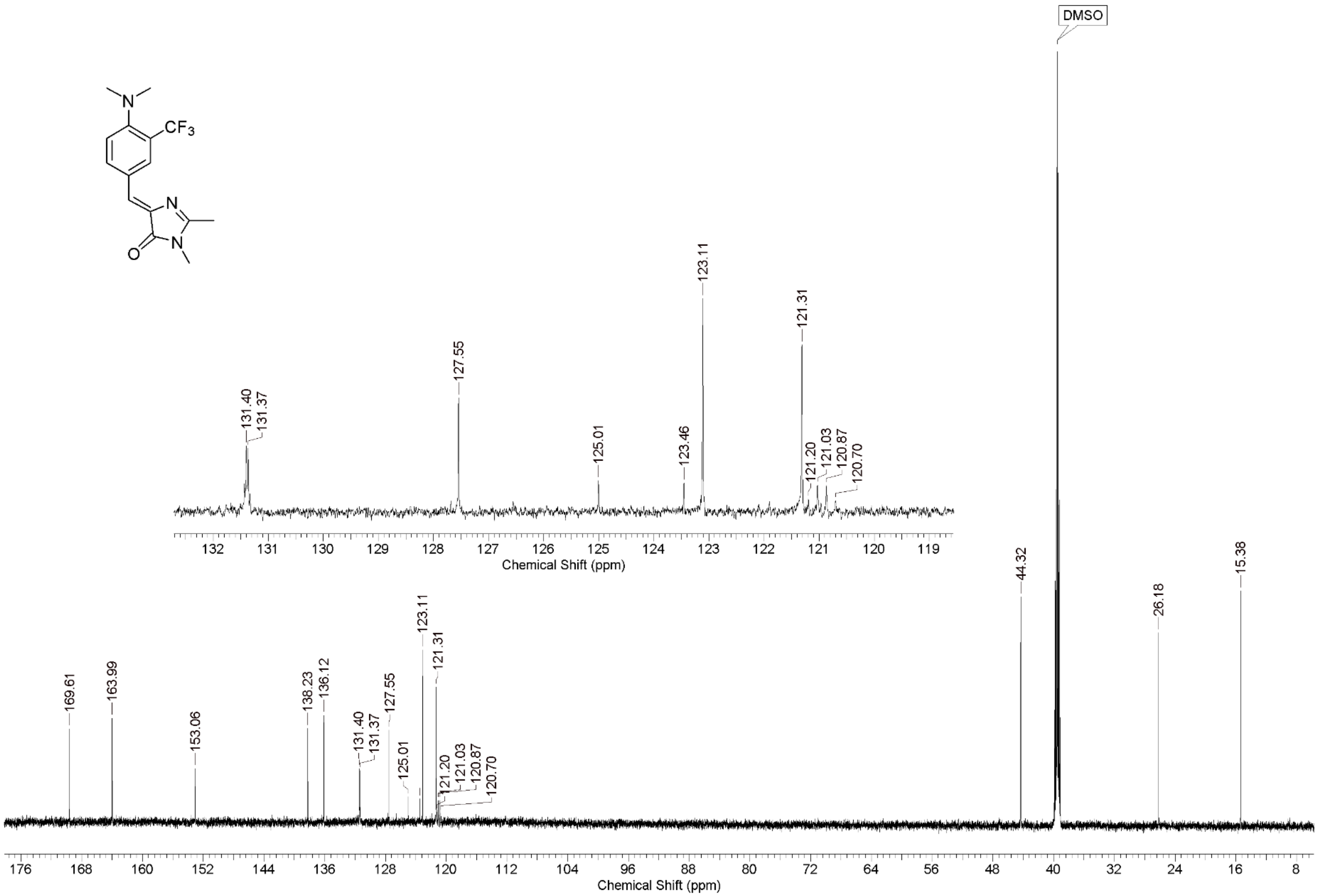
N
CF3
N
N
O
DMSO
131.40
131.37
127.55
125.01
123.46
123.11
121.31
121.20
121.03
120.87
120.70
132 131 130 129 128 127 126 125 124 123 122 121 120 119
Chemical Shift (ppm)
169.61
163.99
153.06
138.23
136.12
131.40
131.37
127.55
125.01
123.11
121.31
121.20
121.03
120.87
120.70
44.32
26.18
15.38
176 168 160 152 144 136 128 120 112 104 96 88 80 72 64 56 48 40 32 24 16 8
Chemical Shift (ppm)

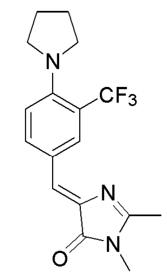

* = E-isomer (at the benzylidene moiety)

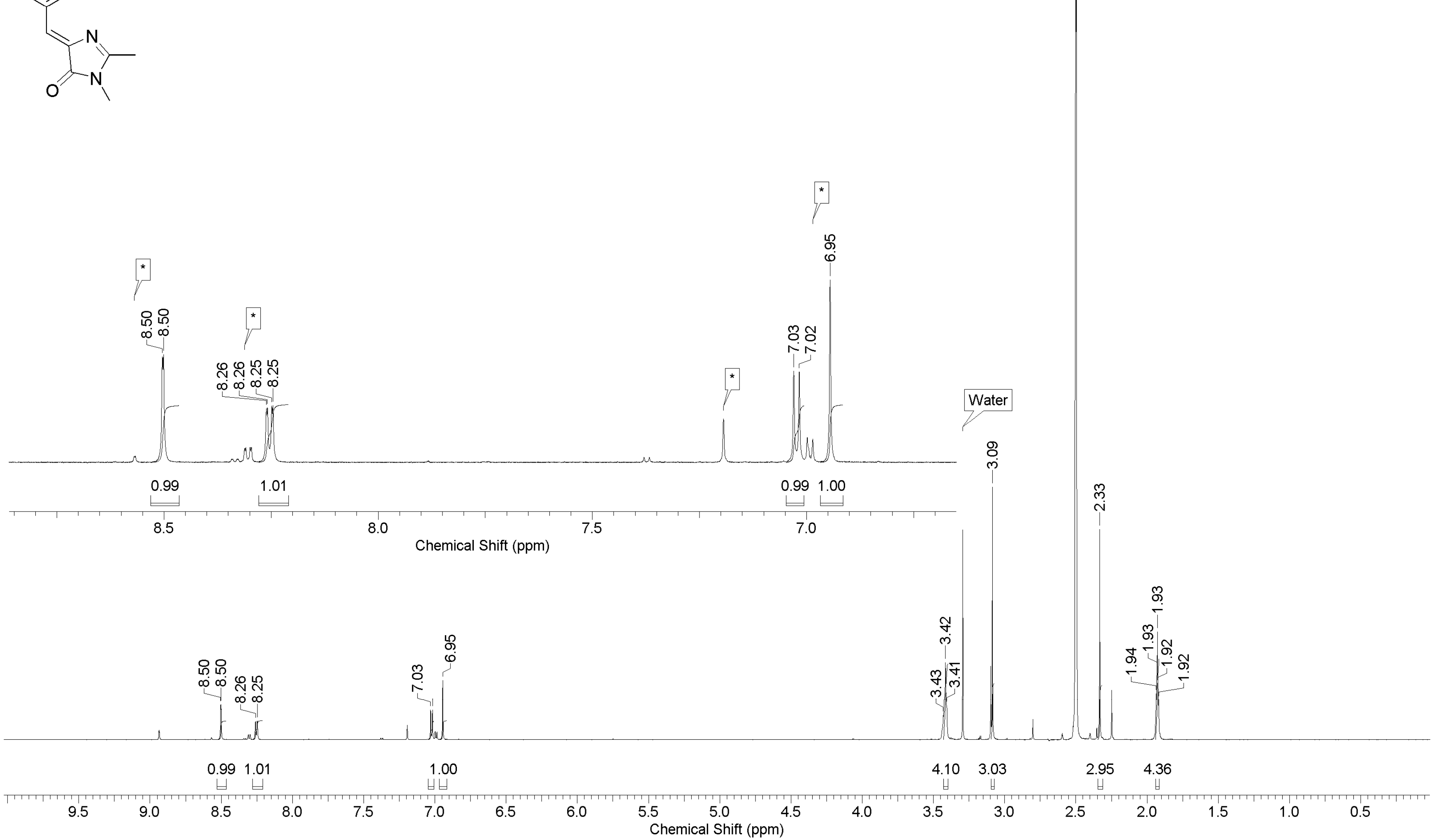

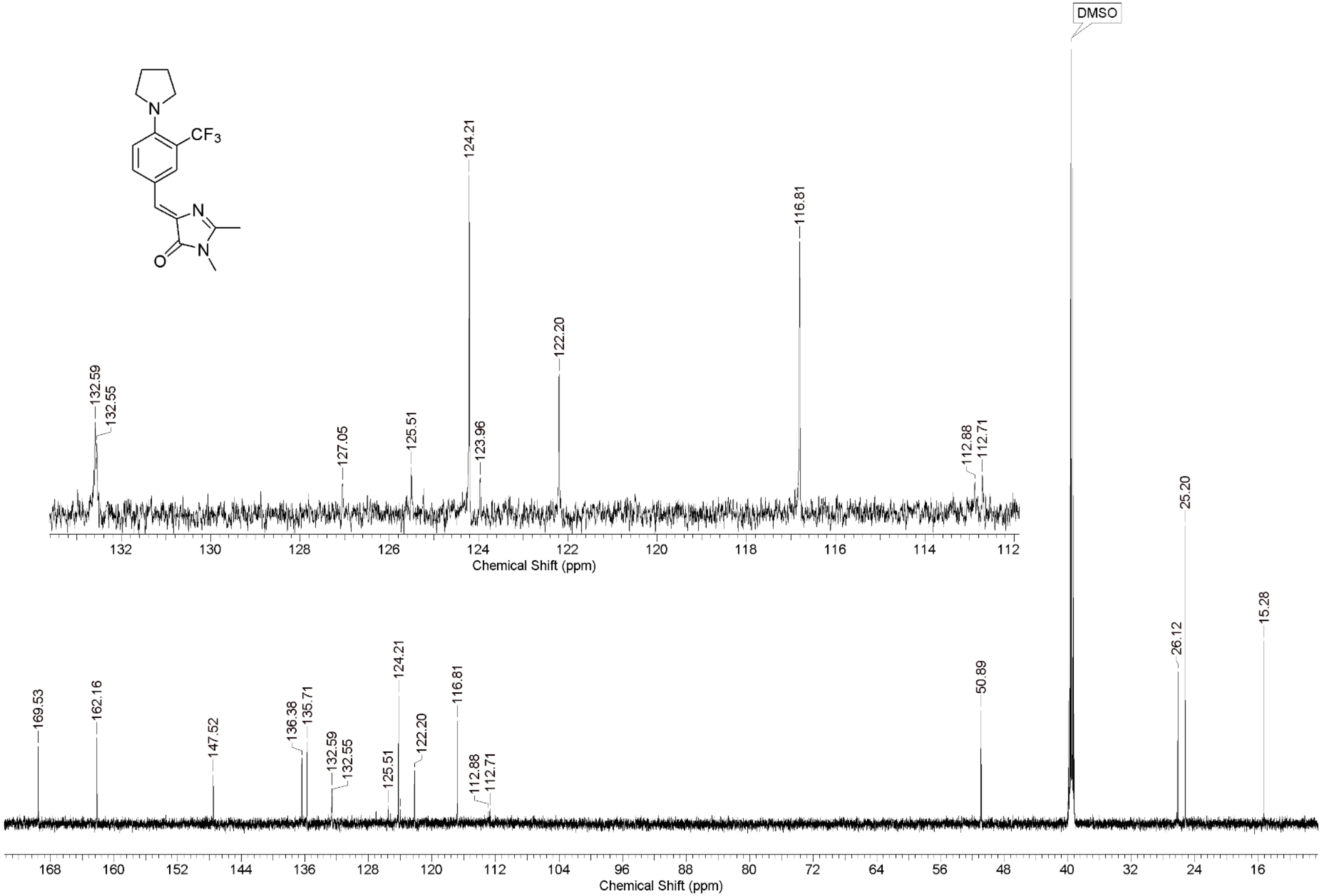

CF3
N
N
O
DMSO
132.59
132.55
127.05
125.51
124.21
123.96
122.20
116.81
112.88
112.71
Chemical Shift (ppm)
169.53
162.16
147.52
136.38
135.71
132.59
132.55
125.51
124.21
122.20
116.81
112.88
112.71
50.89
26.12
25.20
15.28
Chemical Shift (ppm)

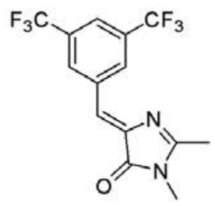

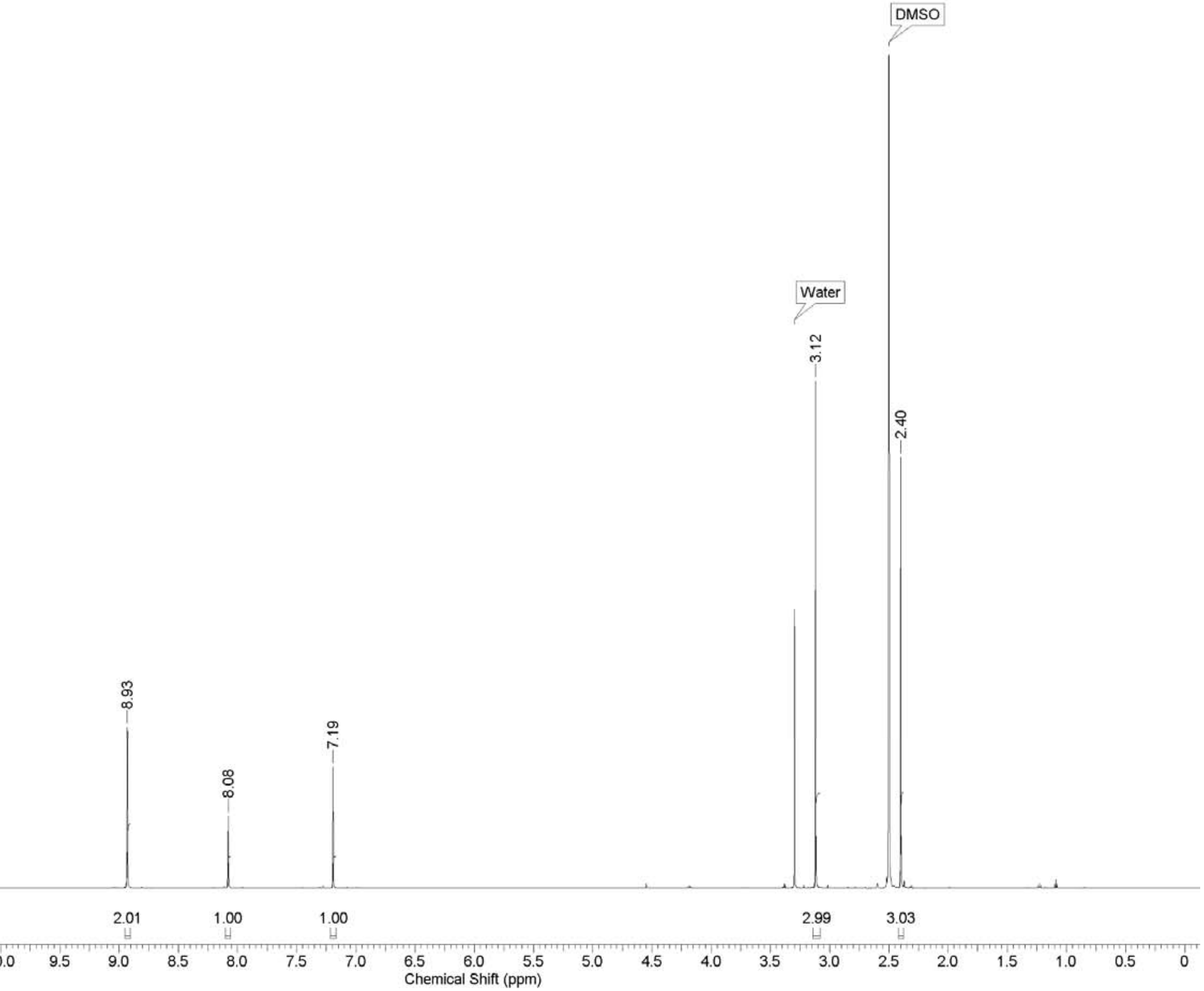

DMSO
Water
8.93
8.08
7.19
3.12
2.40
2.01
1.00
1.00
2.99
3.03
12.0
11.5
11.0
10.5
10.0
9.5
9.0
8.5
8.0
7.5
7.0
6.5
6.0
5.5
5.0
4.5
4.0
3.5
3.0
2.5
2.0
1.5
1.0
0.5
0
Chemical Shift (ppm)

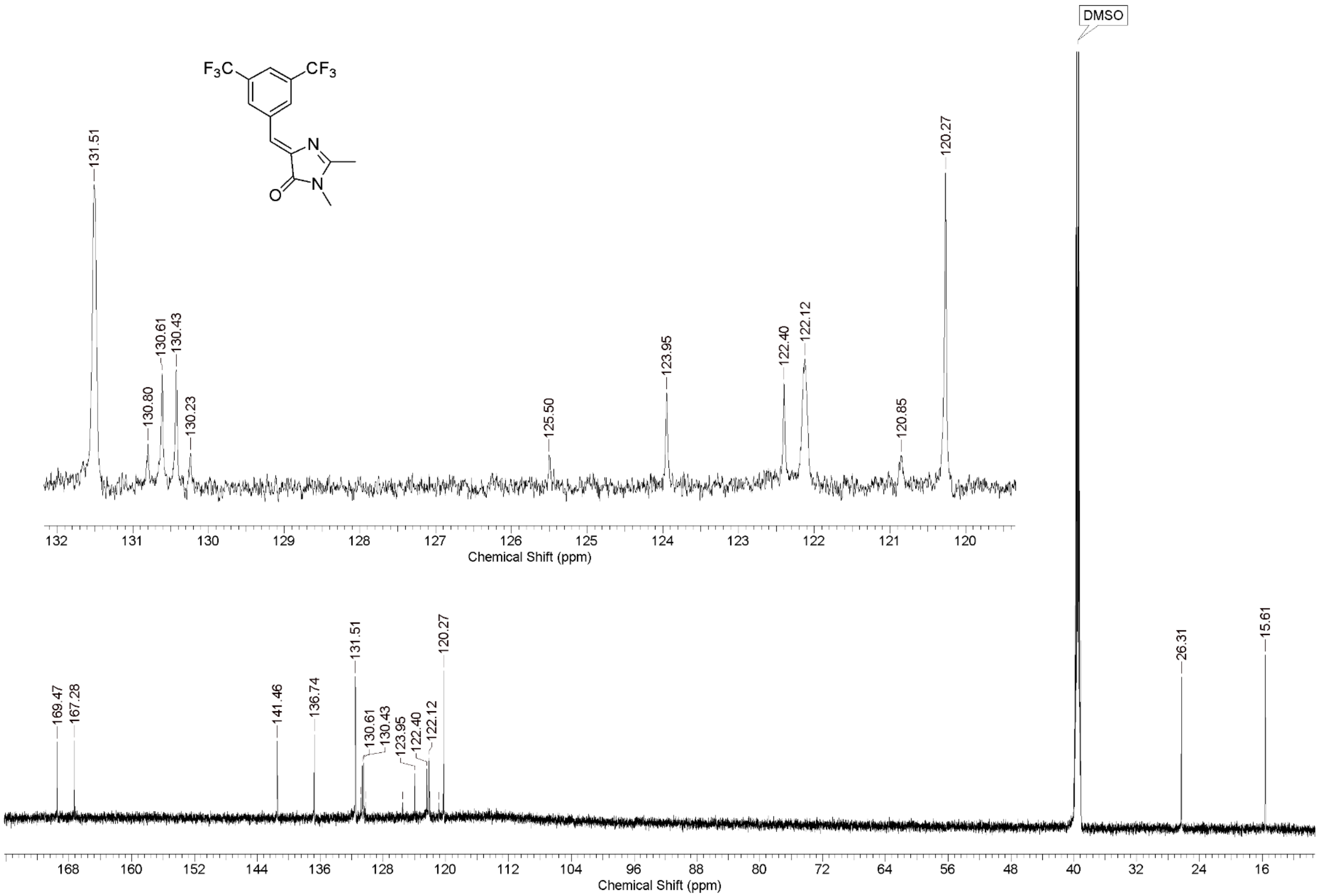

DMSO
F_3C
CF_3
N
N
O
131.51
130.80
130.61
130.43
130.23
125.50
123.95
122.40
122.12
120.85
120.27
132 131 130 129 128 127 126 125 124 123 122 121 120
Chemical Shift (ppm)
169.47
167.28
141.46
136.74
131.51
130.61
130.43
123.95
122.40
122.12
120.27
26.31
15.61
168 160 152 144 136 128 120 112 104 96 88 80 72 64 56 48 40 32 24 16
Chemical Shift (ppm)

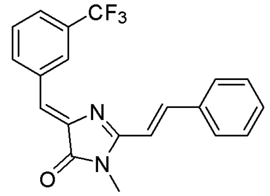

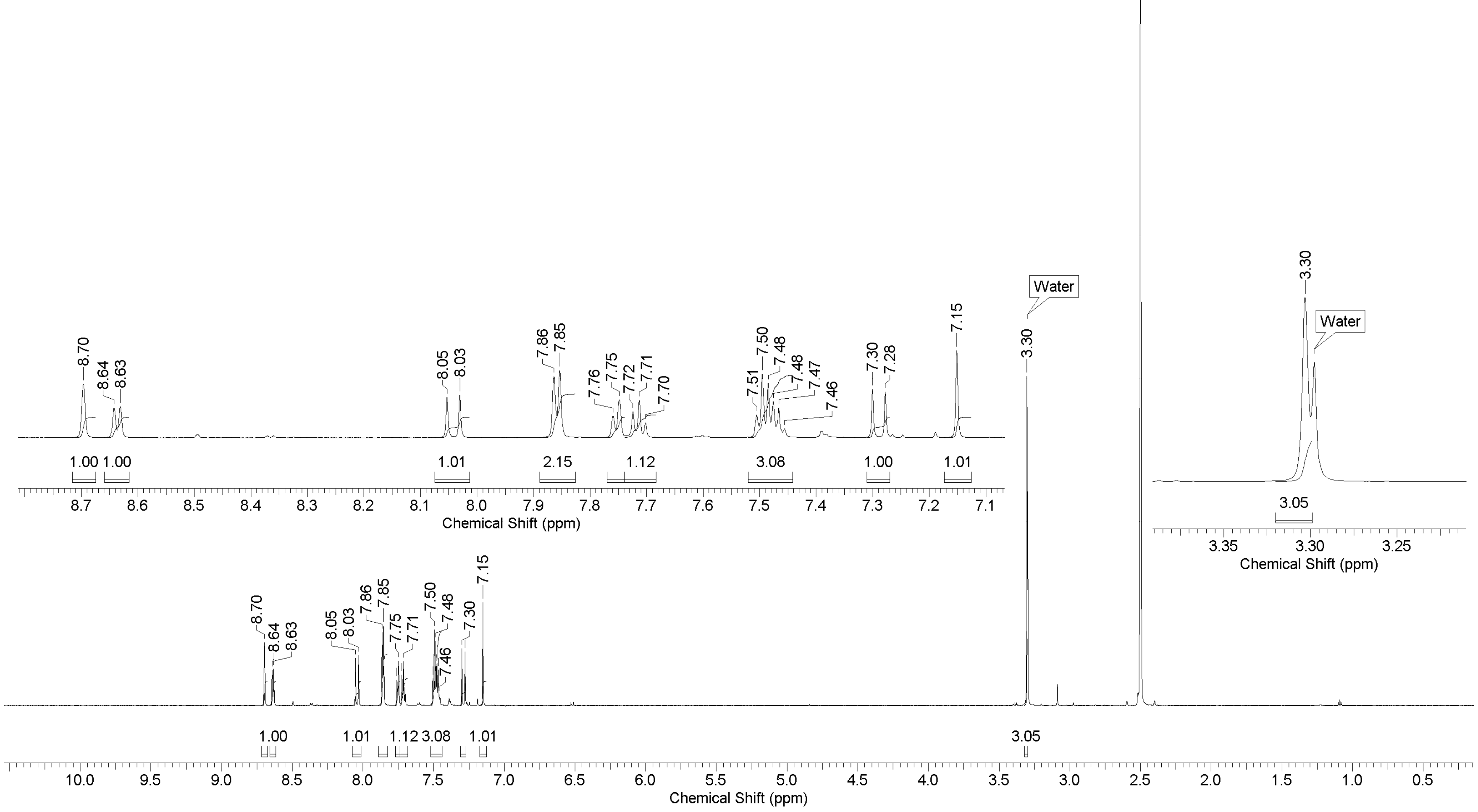

DMSO
Water
3.30
8.70
8.64
8.63
8.05
8.03
7.86
7.85
7.76
7.75
7.72
7.71
7.70
7.51
7.50
7.48
7.48
7.47
7.46
7.30
7.28
7.15
1.00 1.00
1.01
2.15
1.12
3.08
1.00
1.01
Chemical Shift (ppm)
3.05
Chemical Shift (ppm)
Chemical Shift (ppm)

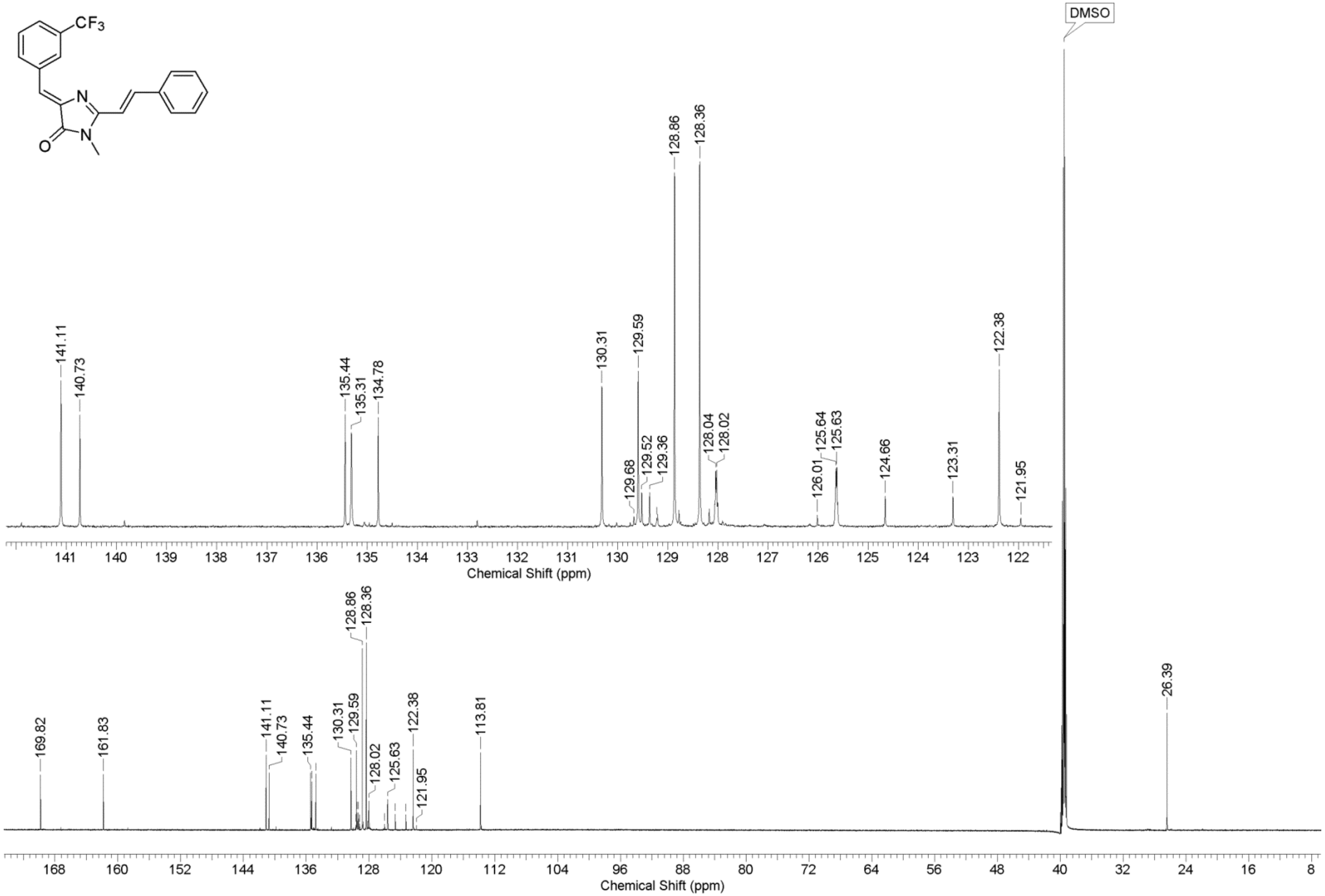
CF3
N
O
N
DMSO
141.11
140.73
135.44
135.31
134.78
130.31
129.68
129.59
129.52
129.36
128.86
128.36
128.04
128.02
126.01
125.64
125.63
124.66
123.31
122.38
121.95
Chemical Shift (ppm)
169.82
161.83
113.81
26.39
Chemical Shift (ppm)

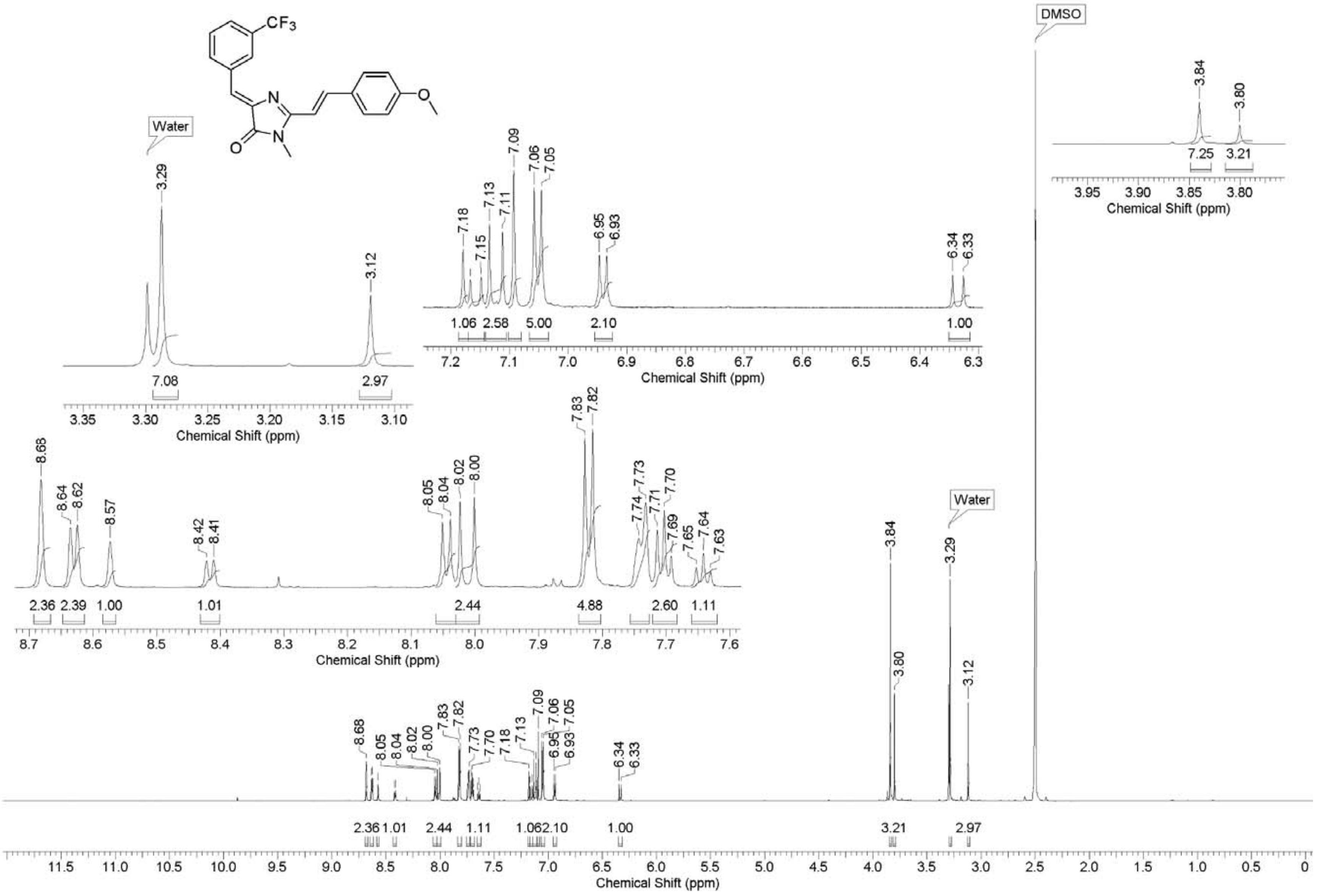
CF3
N
O
N
O
Water
DMSO
Water
Chemical Shift (ppm)
Chemical Shift (ppm)
Chemical Shift (ppm)
Chemical Shift (ppm)
Chemical Shift (ppm)

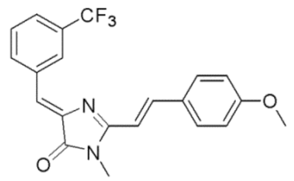

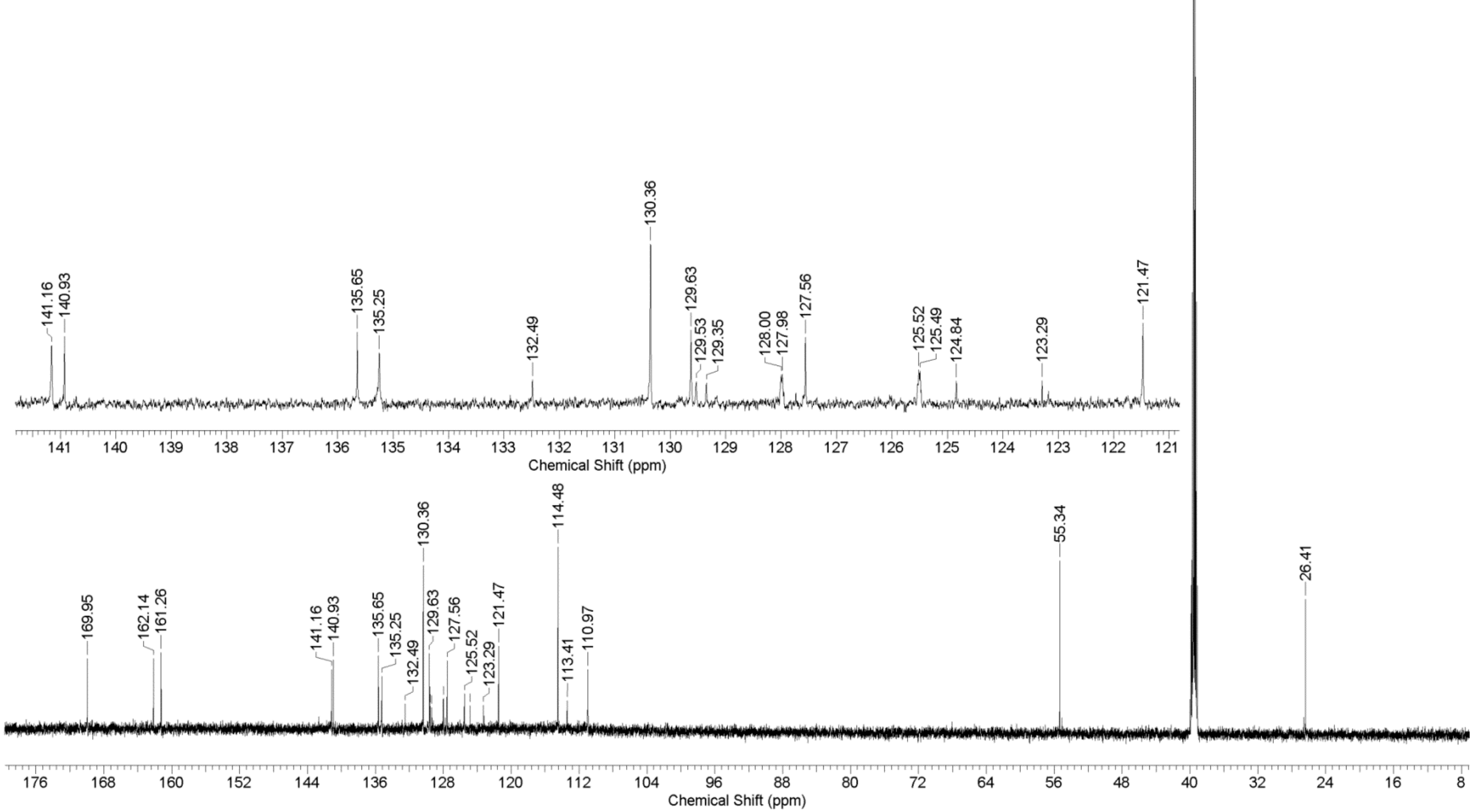
DMSO
141.16
140.93
135.65
135.25
132.49
130.36
129.63
129.53
129.35
128.00
127.98
127.56
125.52
125.49
124.84
123.29
121.47
Chemical Shift (ppm)
169.95
162.14
161.26
114.48
113.41
110.97
55.34
26.41
Chemical Shift (ppm)

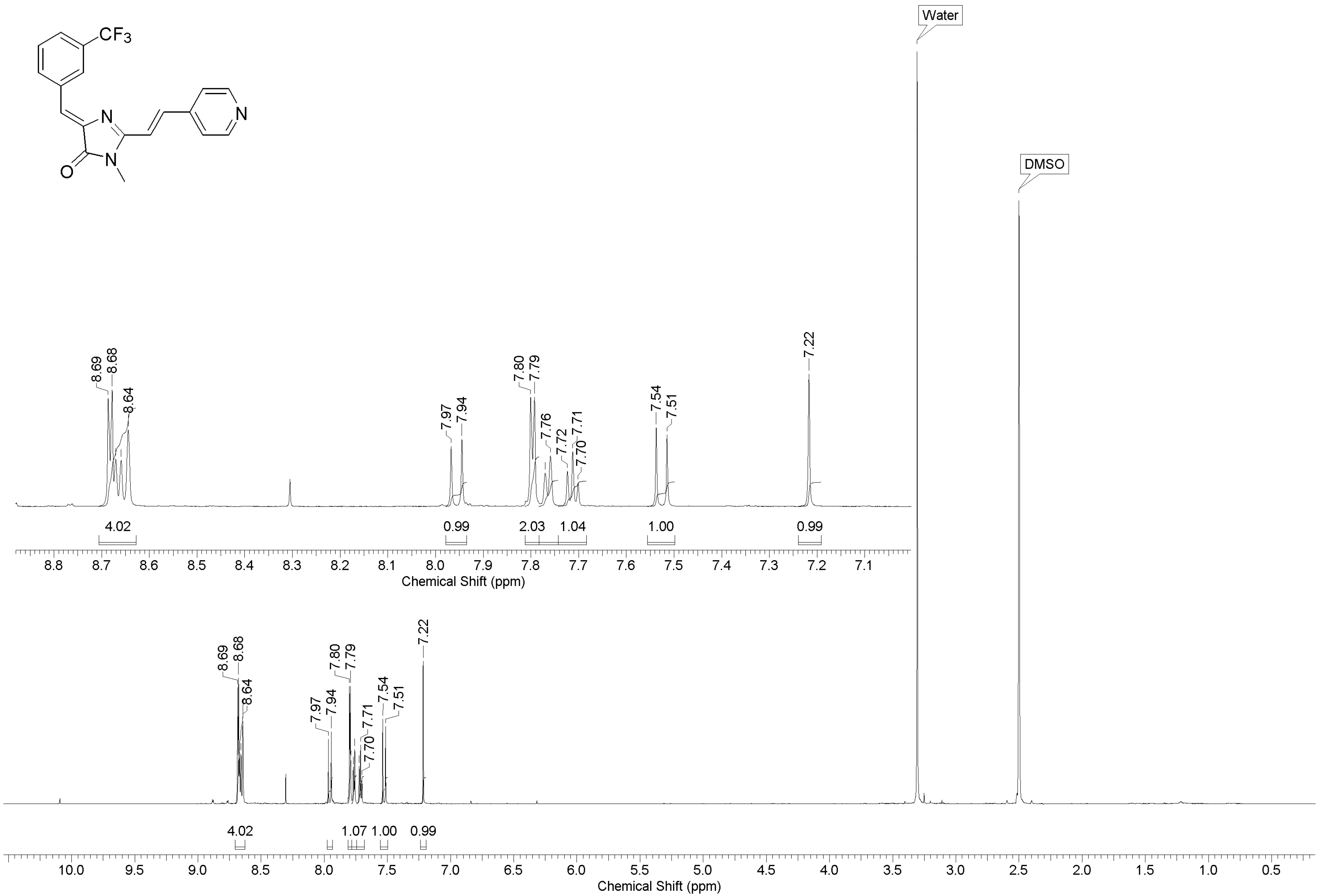
CF3
N
N
O
N
Water
DMSO
8.69
8.68
8.64
7.97
7.94
7.80
7.79
7.76
7.72
7.71
7.70
7.54
7.51
7.22
4.02
0.99
2.03
1.04
1.00
0.99
Chemical Shift (ppm)
4.02
1.07
1.00
0.99
Chemical Shift (ppm)

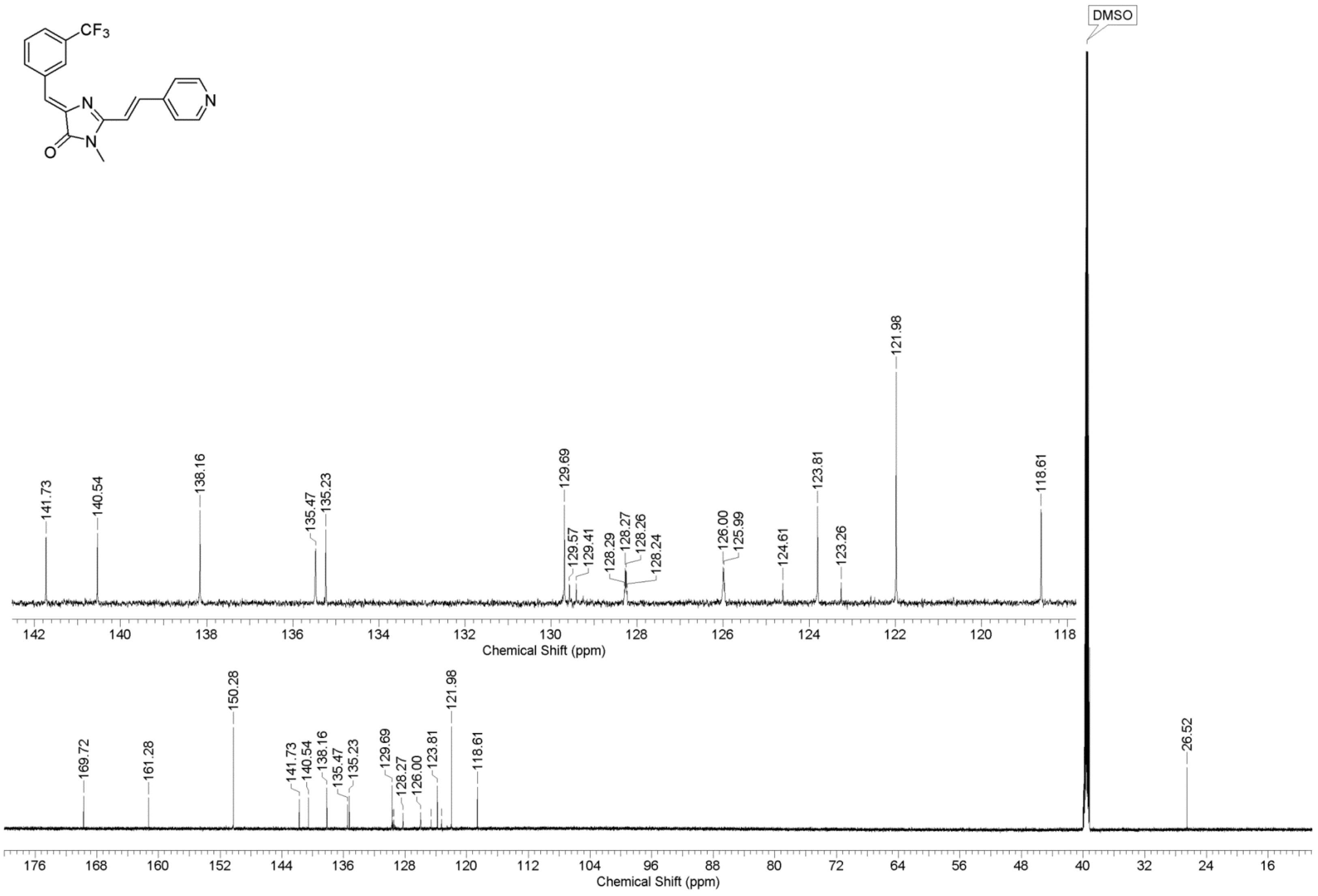
CF3
N
N
O
DMSO
141.73
140.54
138.16
135.47
135.23
129.69
129.57
129.41
128.29
128.27
128.26
128.24
126.00
125.99
124.61
123.81
123.26
121.98
118.61
Chemical Shift (ppm)
169.72
161.28
150.28
141.73
140.54
138.16
135.47
135.23
129.69
128.27
126.00
123.81
121.98
118.61
26.52
Chemical Shift (ppm)

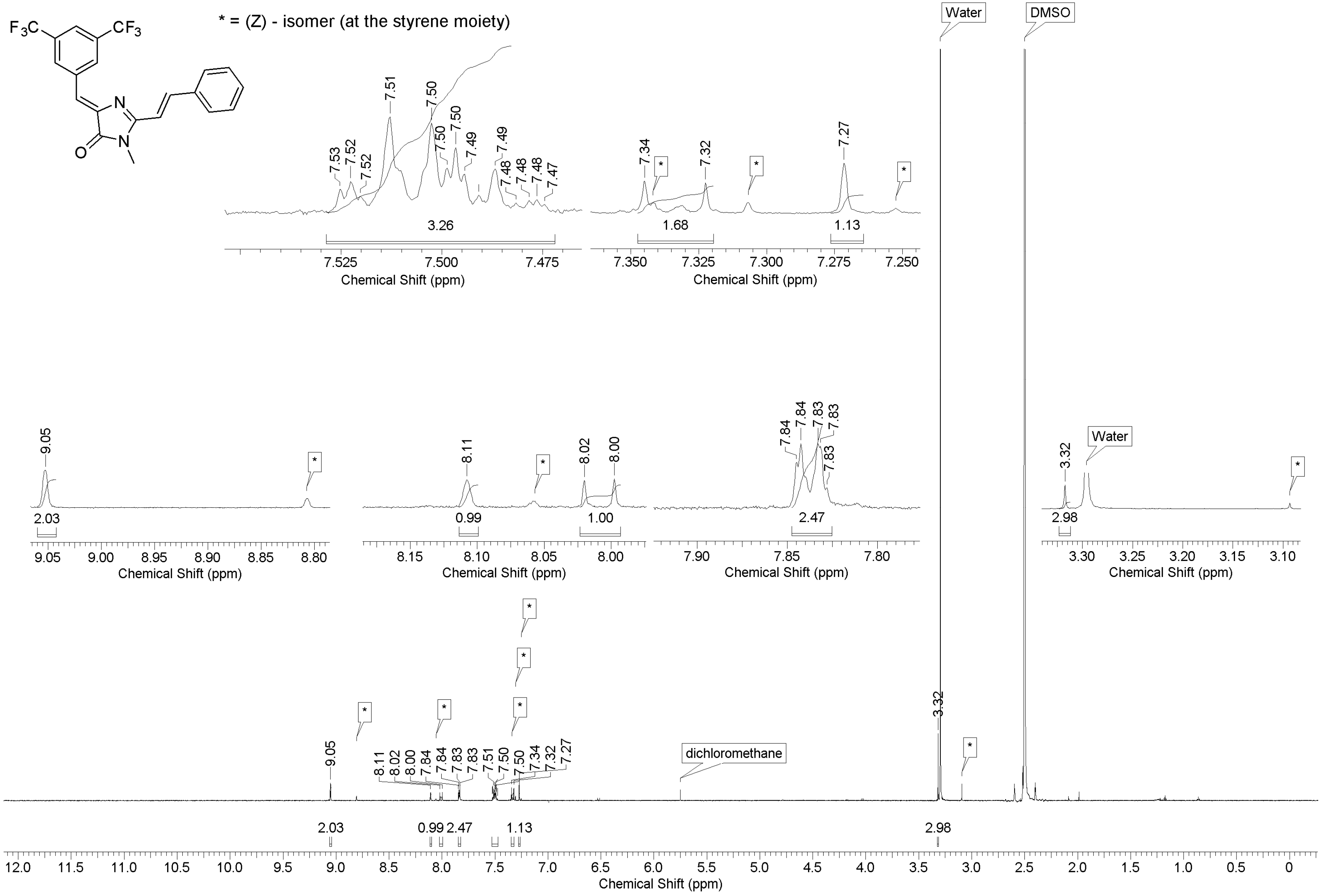

F3C
CF3
N
N
O
* = (Z) - isomer (at the styrene moiety)
Water
DMSO
dichloromethane
3.26
1.68
1.13
2.03
0.99
1.00
2.47
2.98
Chemical Shift (ppm)

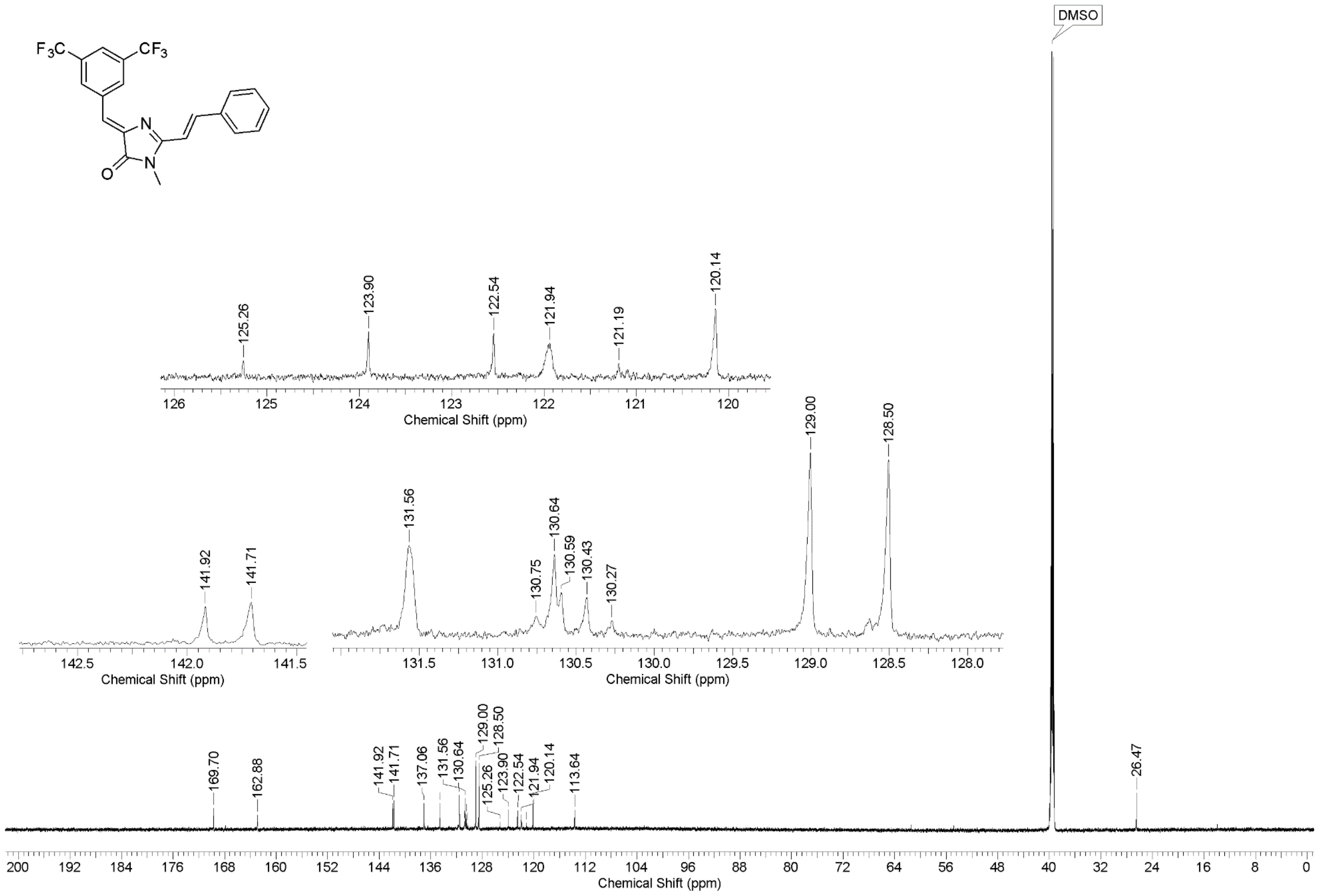

F3C
CF3
N
N
O
DMSO
125.26
123.90
122.54
121.94
121.19
120.14
Chemical Shift (ppm)
141.92
141.71
131.56
130.75
130.64
130.59
130.43
130.27
129.00
128.50
169.70
162.88
137.06
113.64
26.47

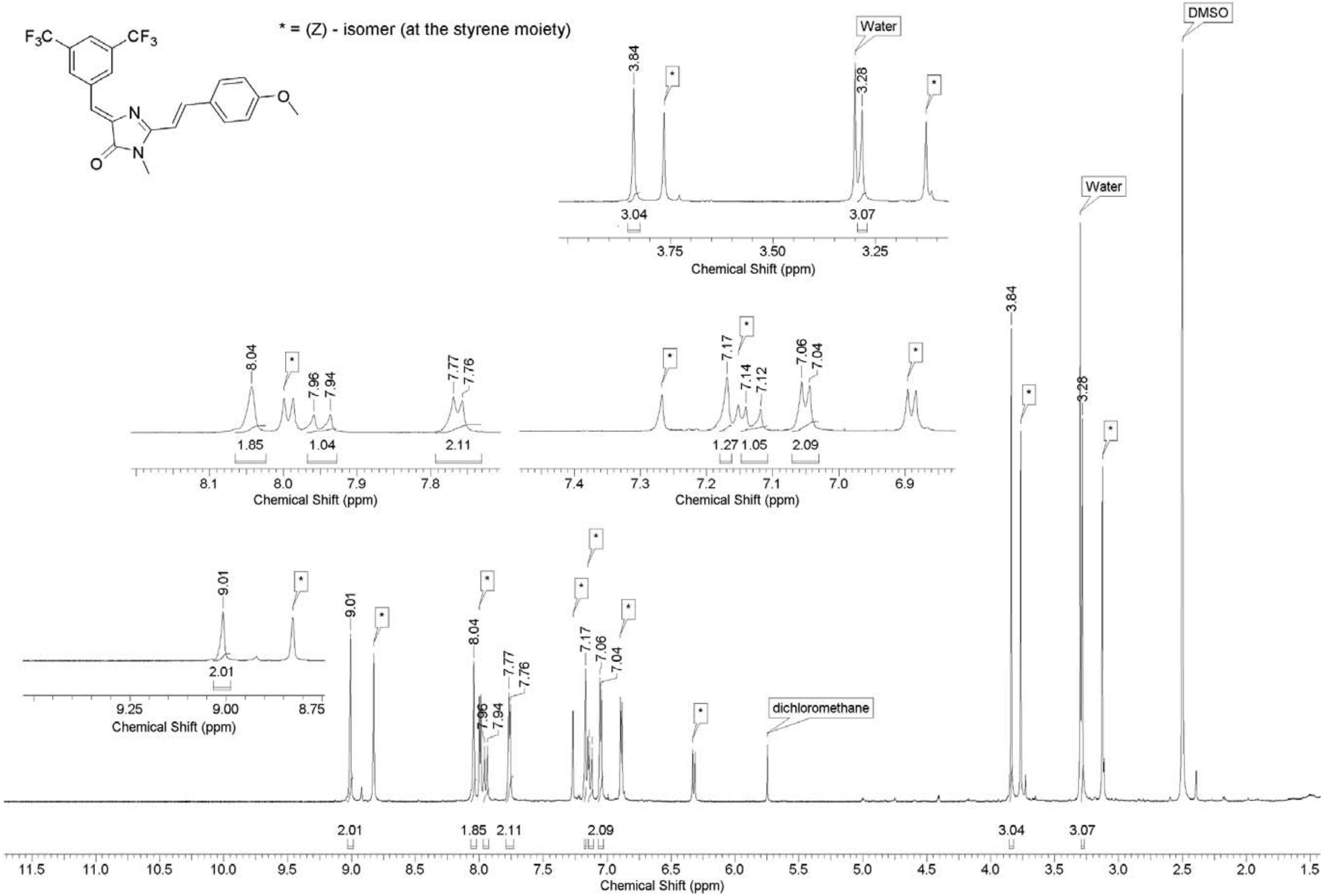

F_3C
CF_3
N
O
N
* = (Z) - isomer (at the styrene moiety)
DMSO
Water
Water
dichloromethane
9.01
8.04
7.96
7.94
7.77
7.76
7.17
7.14
7.12
7.06
7.04
3.84
3.28
2.01
1.85
1.04
2.11
1.27
1.05
2.09
3.04
3.07
Chemical Shift (ppm)

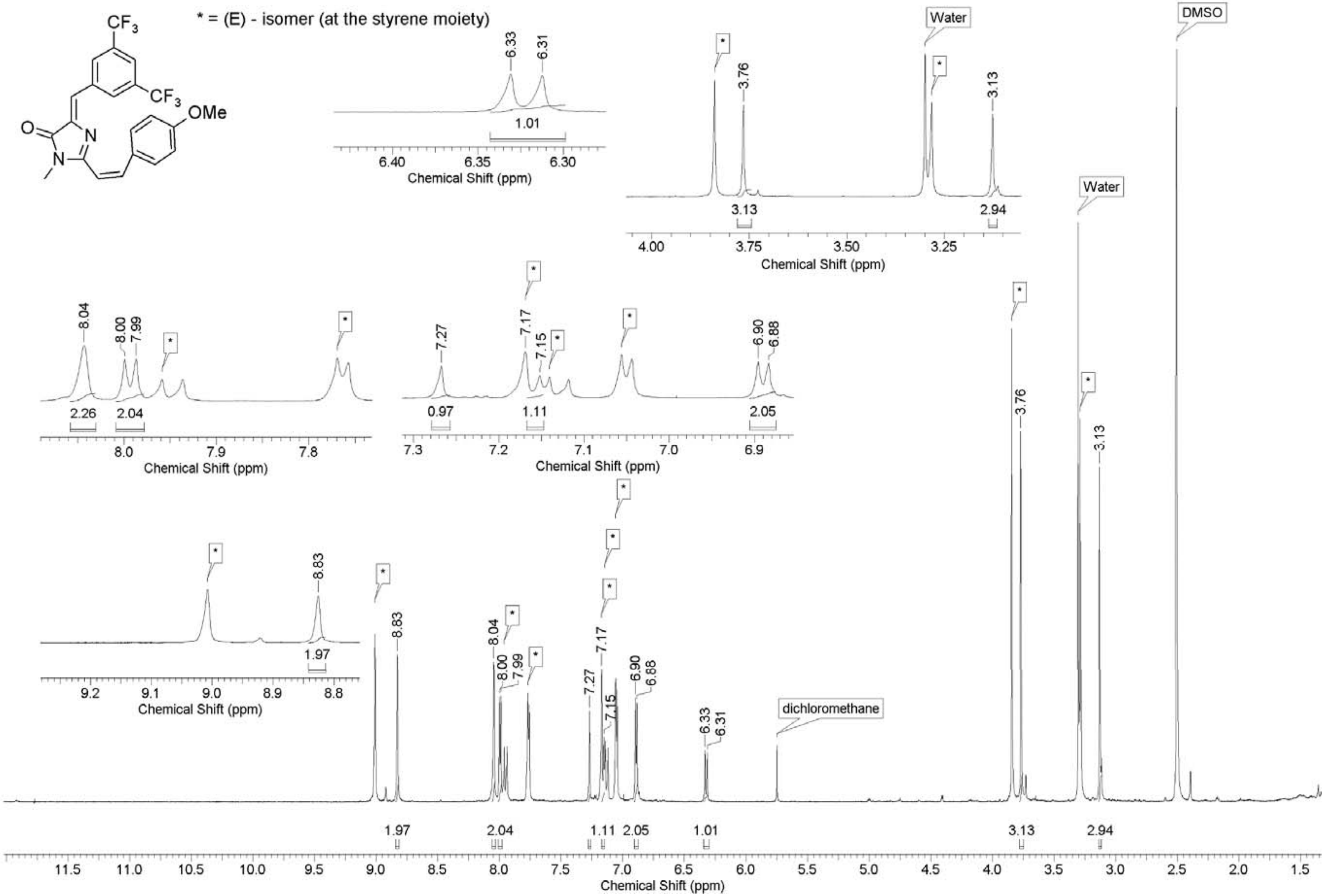
* = (E) - isomer (at the styrene moiety)
CF3
CF3
OMe
O
N
N
6.33
6.31
1.01
Chemical Shift (ppm)
Water
DMSO
3.76
3.13
3.13
2.94
8.04
8.00
7.99
2.26
2.04
7.27
7.17
7.15
6.90
6.88
0.97
1.11
2.05
8.83
1.97
dichloromethane
Chemical Shift (ppm)

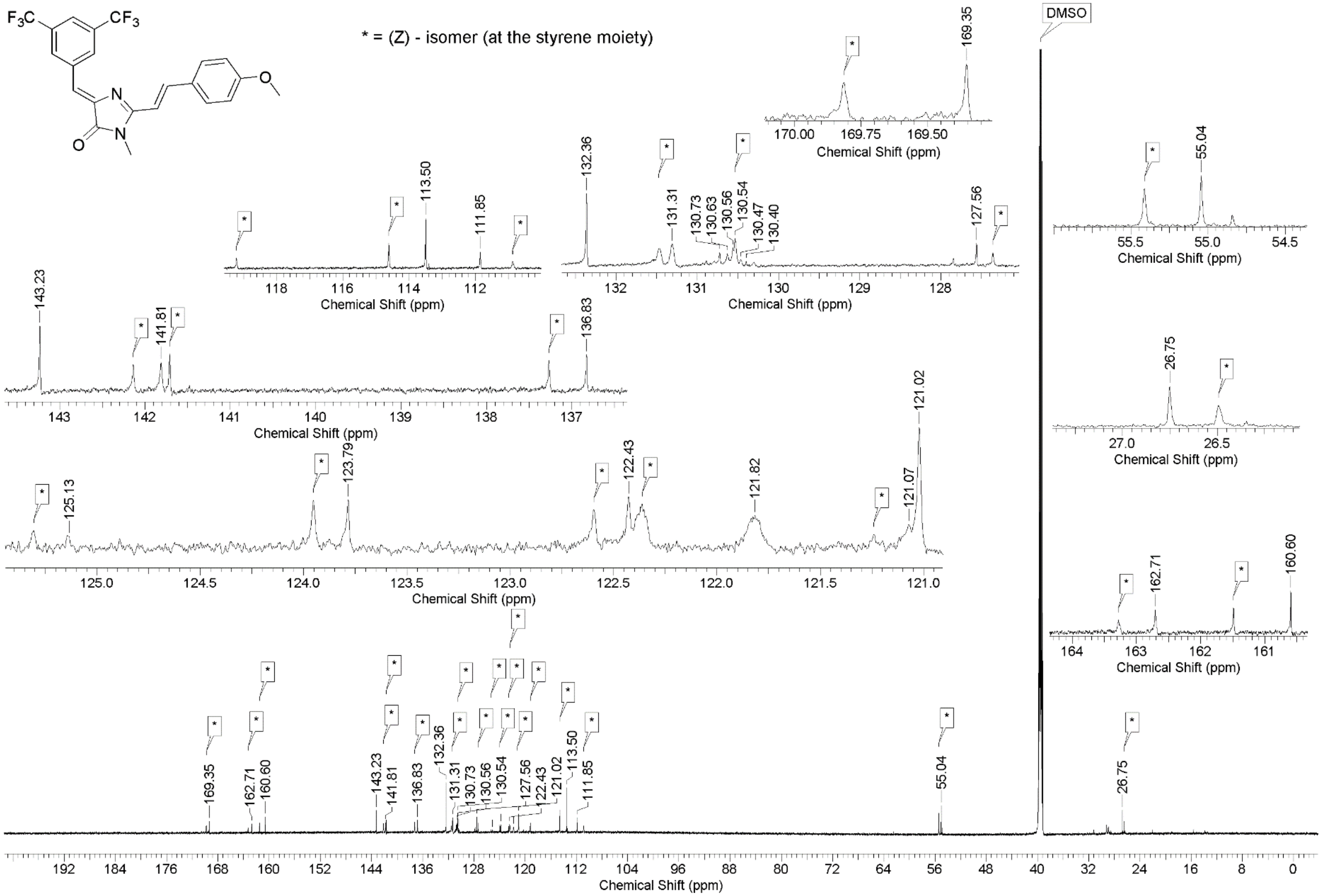

F3C
CF3
N
N
O
O
* = (Z) - isomer (at the styrene moiety)
DMSO
169.35
170.00 169.75 169.50
Chemical Shift (ppm)
55.04
55.5 55.0 54.5
Chemical Shift (ppm)
113.50
111.85
118 116 114 112
Chemical Shift (ppm)
132.36
131.31
130.73
130.63
130.56
130.54
130.47
130.40
127.56
132 131 130 129 128
Chemical Shift (ppm)
143.23
141.81
136.83
143 142 141 140 139 138 137
Chemical Shift (ppm)
26.75
27.0 26.5
Chemical Shift (ppm)
125.13
123.79
122.43
121.82
121.07
121.02
125.0 124.5 124.0 123.5 123.0 122.5 122.0 121.5 121.0
Chemical Shift (ppm)
162.71
160.60
164 163 162 161
Chemical Shift (ppm)
169.35
162.71
160.60
143.23
141.81
136.83
132.36
131.31
130.73
130.56
130.54
127.56
122.43
121.02
113.50
111.85
55.04
26.75
192 184 176 168 160 152 144 136 128 120 112 104 96 88 80 72 64 56 48 40 32 24 16 8 0
Chemical Shift (ppm)

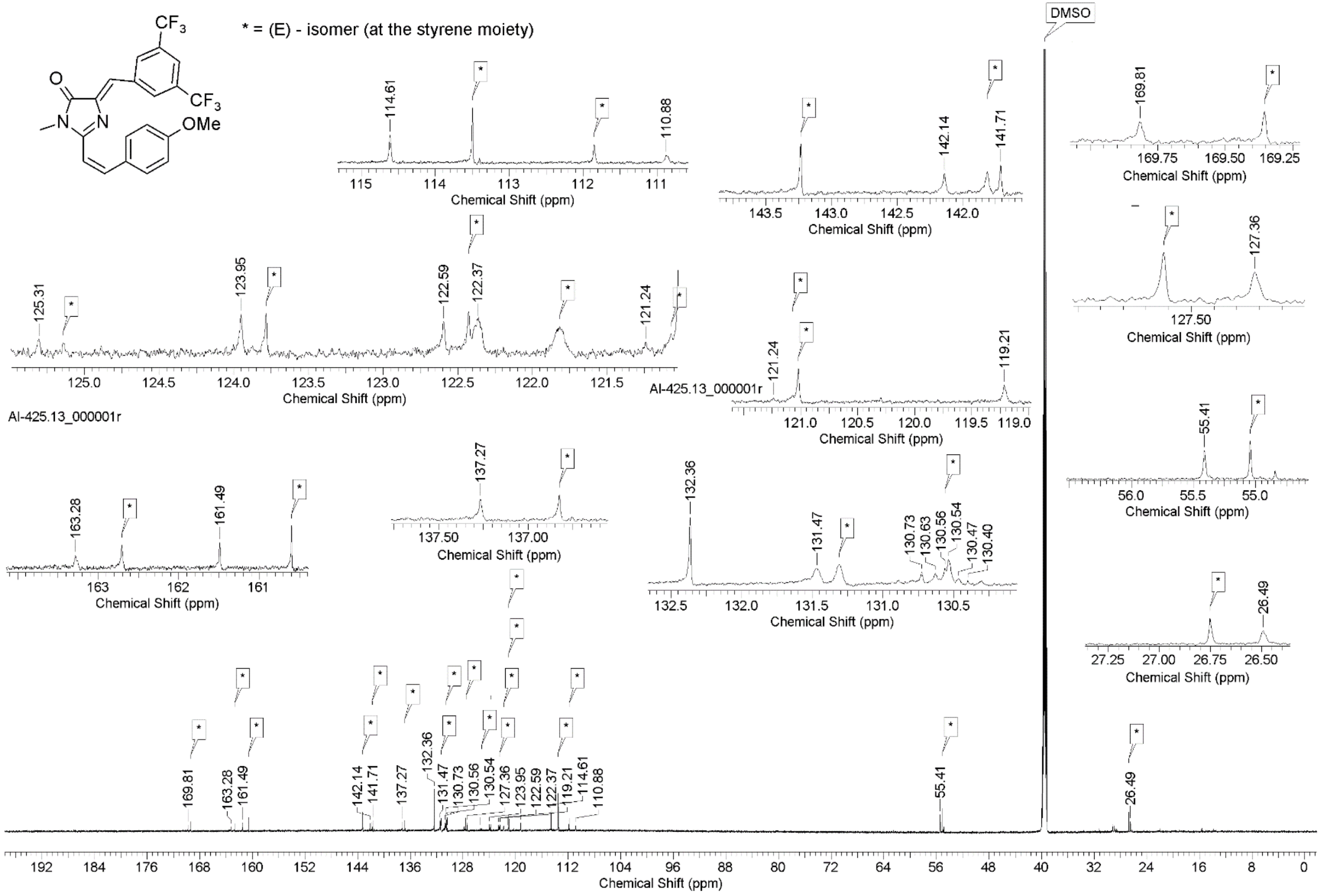

* = (E) - isomer (at the styrene moiety)
DMSO
AI-425.13_000001r
Chemical Shift (ppm)
169.81
163.28
161.49
142.14
141.71
137.27
132.36
131.47
130.73
130.56
130.54
127.36
123.95
122.59
122.37
119.21
114.61
110.88
55.41
26.49

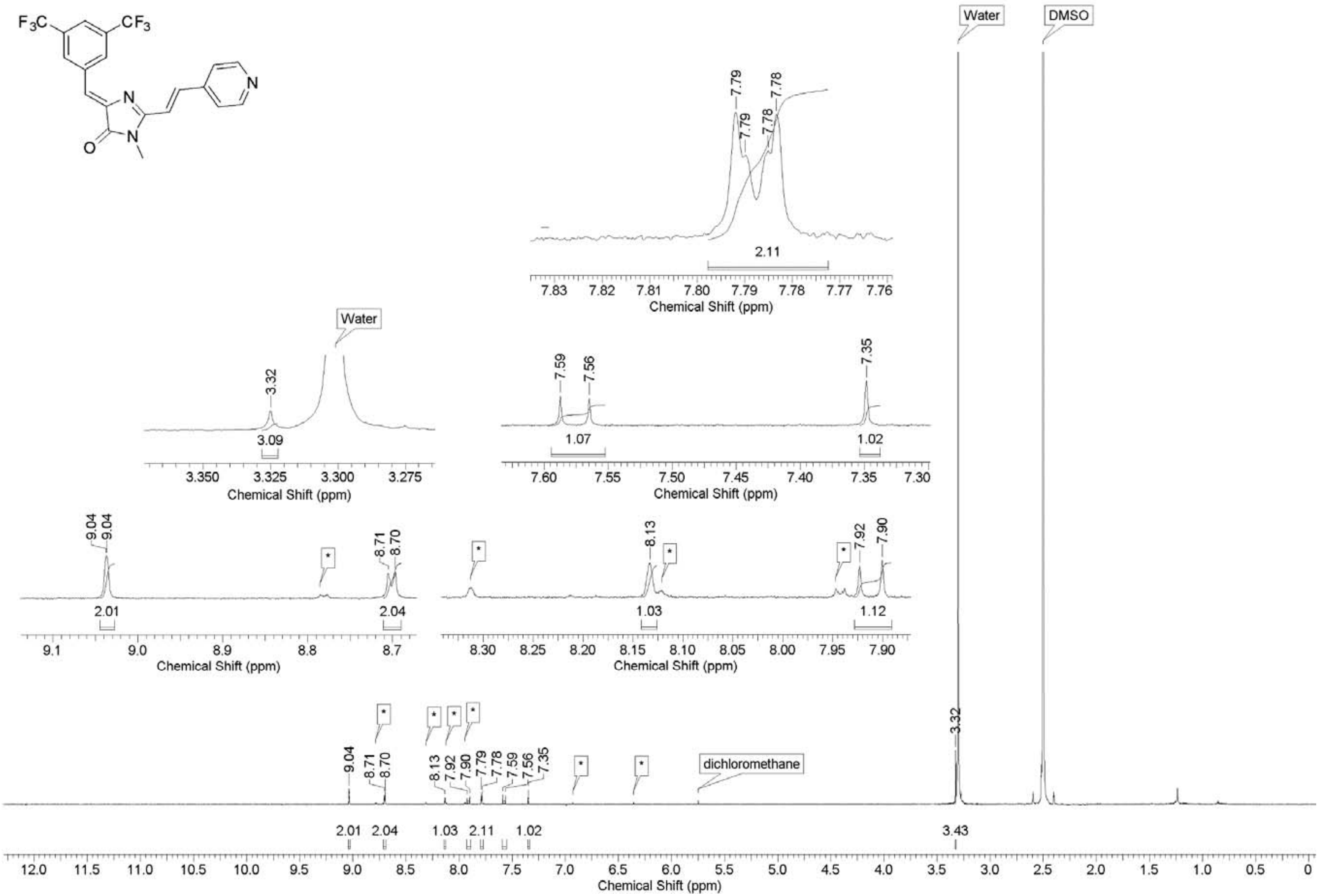

F3C
CF3
N
N
O
Water
DMSO
2.11
7.83 7.82 7.81 7.80 7.79 7.78 7.77 7.76
Chemical Shift (ppm)
3.09
3.350 3.325 3.300 3.275
Chemical Shift (ppm)
1.07
1.02
7.60 7.55 7.50 7.45 7.40 7.35 7.30
Chemical Shift (ppm)
2.01
2.04
9.1 9.0 8.9 8.8 8.7
Chemical Shift (ppm)
1.03
1.12
8.30 8.25 8.20 8.15 8.10 8.05 8.00 7.95 7.90
Chemical Shift (ppm)
dichloromethane
2.01 2.04 1.03 2.11 1.02 3.43
12.0 11.5 11.0 10.5 10.0 9.5 9.0 8.5 8.0 7.5 7.0 6.5 6.0 5.5 5.0 4.5 4.0 3.5 3.0 2.5 2.0 1.5 1.0 0.5 0
Chemical Shift (ppm)

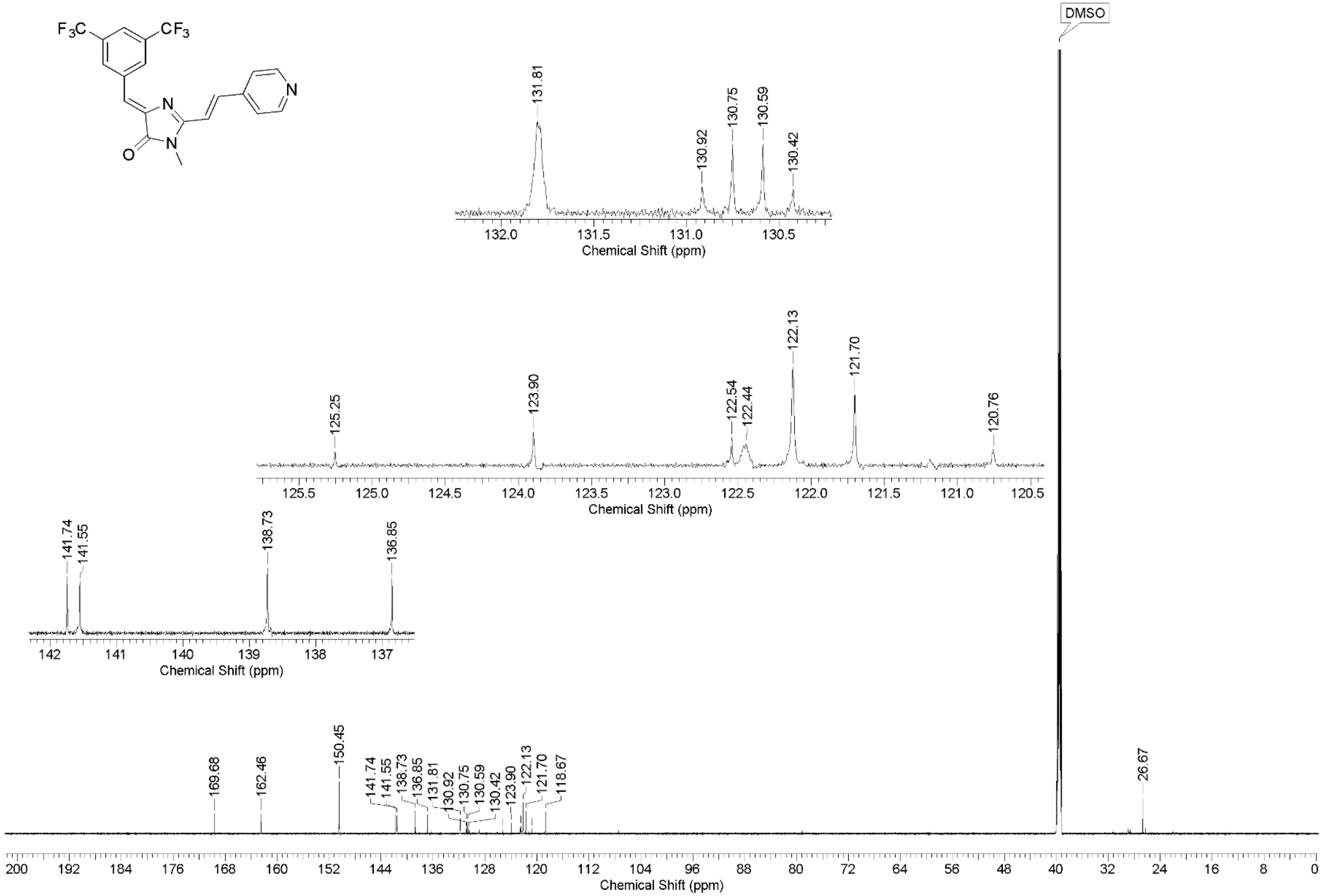
DMSO
131.81
130.92
130.75
130.59
130.42
132.0
131.5
131.0
130.5
Chemical Shift (ppm)
125.25
123.90
122.54
122.44
122.13
121.70
120.76
125.5
125.0
124.5
124.0
123.5
123.0
122.5
122.0
121.5
121.0
120.5
Chemical Shift (ppm)
141.74
141.55
138.73
136.85
142
141
140
139
138
137
Chemical Shift (ppm)
169.68
162.46
150.45
141.74
141.55
138.73
136.85
131.81
130.92
130.75
130.59
130.42
123.90
122.13
121.70
118.67
26.67
200
192
184
176
168
160
152
144
136
128
120
112
104
96
88
80
72
64
56
48
40
32
24
16
8
0
Chemical Shift (ppm)

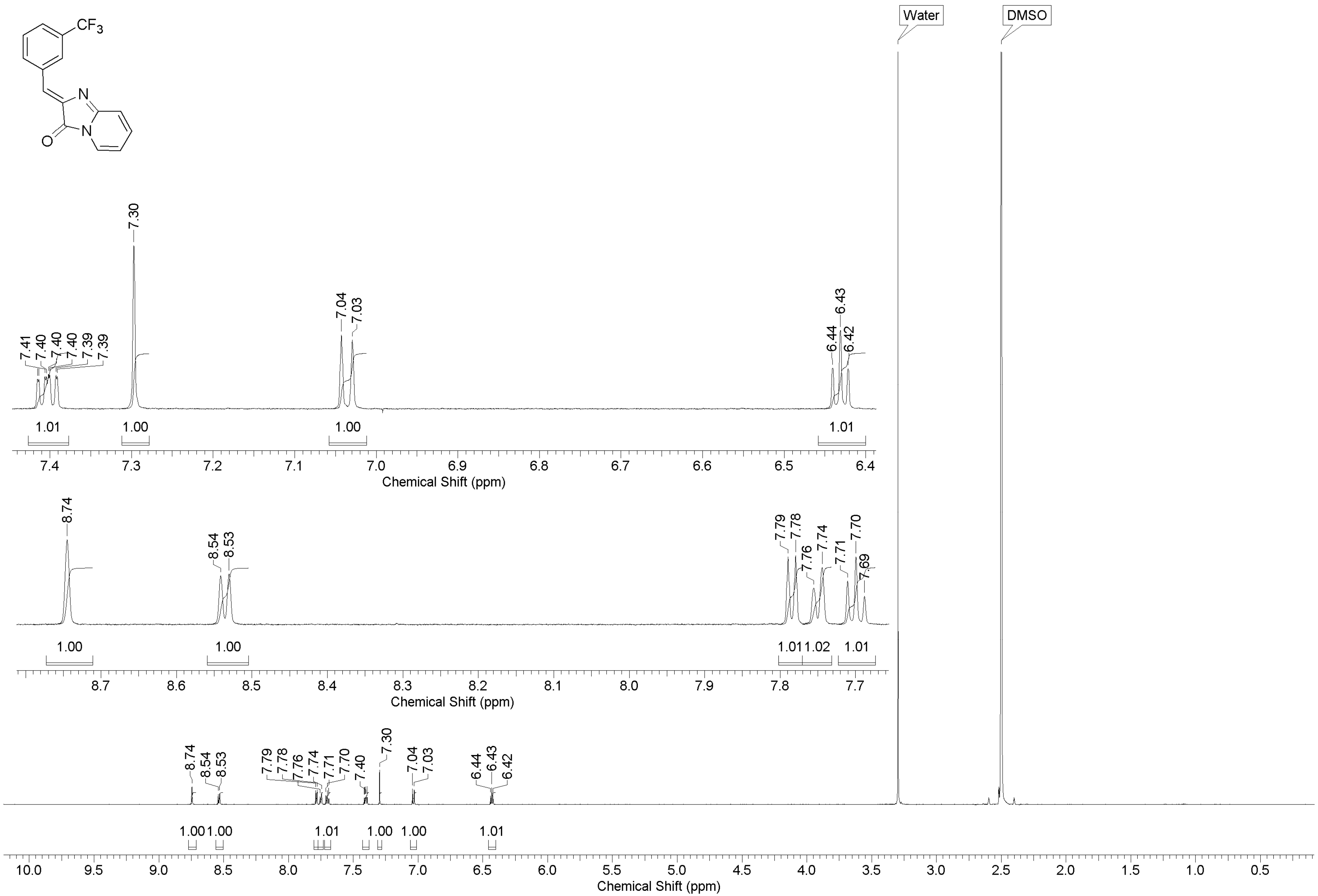
CF3
N
N
O
Water
DMSO
7.41 7.40 7.40 7.40 7.39 7.39
7.30
7.04 7.03
6.44 6.43 6.42
1.01 1.00 1.00 1.01
7.4 7.3 7.2 7.1 7.0 6.9 6.8 6.7 6.6 6.5 6.4
Chemical Shift (ppm)
8.74
8.54 8.53
7.79 7.78 7.76 7.74 7.71 7.70 7.69
1.00 1.00 1.01 1.02 1.01
8.7 8.6 8.5 8.4 8.3 8.2 8.1 8.0 7.9 7.8 7.7
Chemical Shift (ppm)
8.74 8.54 8.53 7.79 7.78 7.76 7.74 7.71 7.70 7.40 7.30 7.04 7.03 6.44 6.43 6.42
1.00 1.00 1.01 1.00 1.00 1.01
10.0 9.5 9.0 8.5 8.0 7.5 7.0 6.5 6.0 5.5 5.0 4.5 4.0 3.5 3.0 2.5 2.0 1.5 1.0 0.5
Chemical Shift (ppm)

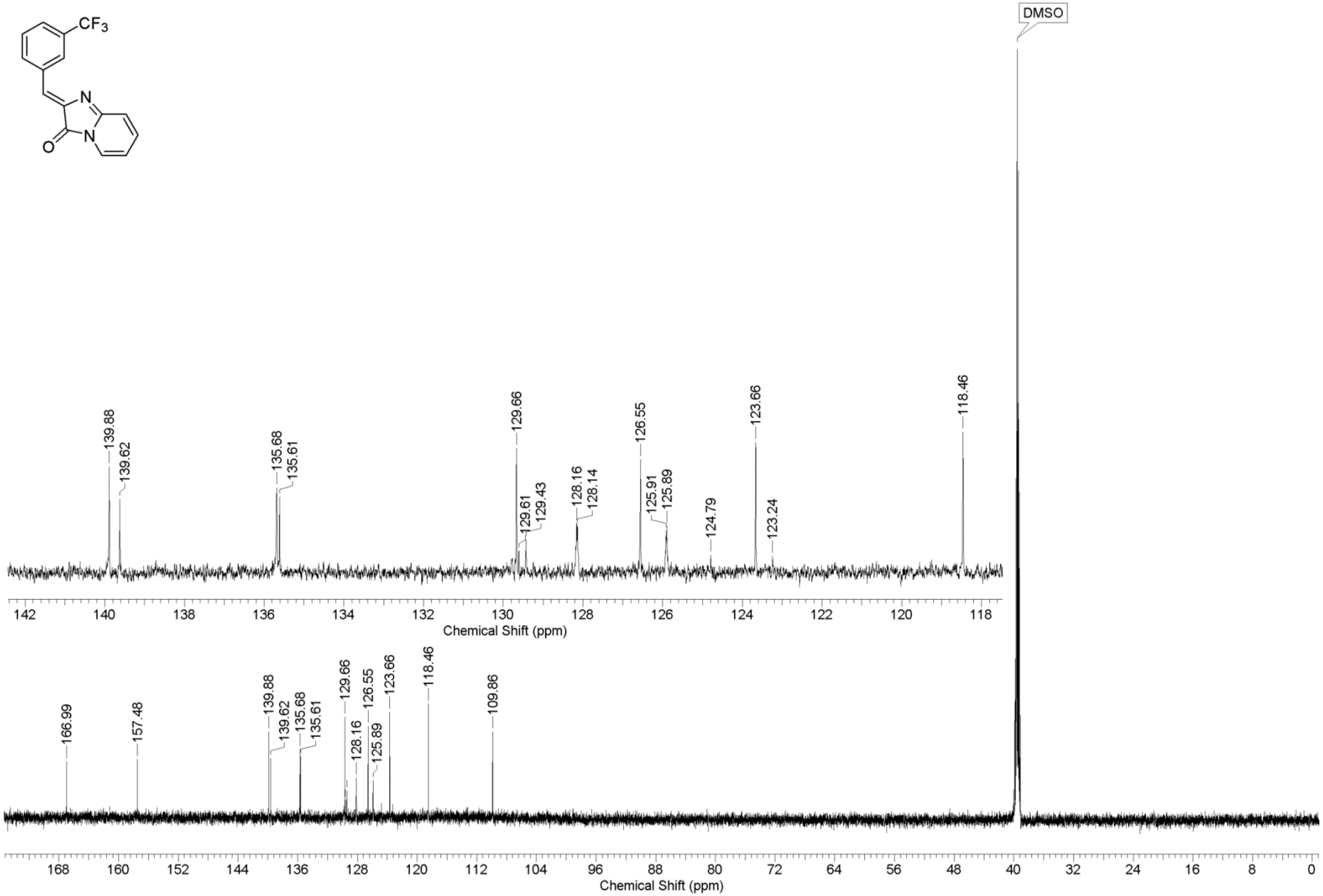
CF3
N
N
O
DMSO
139.88
139.62
135.68
135.61
129.66
129.61
129.43
128.16
128.14
126.55
125.91
125.89
124.79
123.66
123.24
118.46
Chemical Shift (ppm)
166.99
157.48
139.88
139.62
135.68
135.61
129.66
128.16
126.55
125.89
123.66
118.46
109.86
Chemical Shift (ppm)
